# Design and Synthesis of Pyridine-Based Pyrrolo[2,3-*d*]pyrimidine Analogs as CSF1R Inhibitors: Molecular Hybridization and Scaffold Hopping Approach

**DOI:** 10.3390/ph18060814

**Published:** 2025-05-28

**Authors:** Srinivasulu Cherukupalli, Carsten Degenhart, Peter Habenberger, Anke Unger, Jan Eickhoff, Bård Helge Hoff, Eirik Sundby

**Affiliations:** 1 Department of Chemistry, Norwegian University of Science and Technology (NTNU), Høgskoleringen 5, NO-7491 Trondheim, Norway; srinivasulu.cherukupalli@ntnu.no; 2 Lead Discovery Center GmbH (LDC), Otto-Hahn-Strasse 15, 44227 Dortmund, Germanyeickhoff@lead-discovery.de (J.E.); 3 Department of Material Science, Norwegian University of Science and Technology (NTNU), NO-7491 Trondheim, Norway

**Keywords:** CSF1R inhibitors, pyridine, pyrrolo[2,3-*d*]pyrimidine, scaffold hopping, molecular hybridization, Pexidartinib

## Abstract

**Background/Objectives**: Colony stimulating factor 1 receptor kinase (CSF1R) is a well-validated molecular target in drug discovery for various reasons. Based on the structure of an early lead molecule identified in our lab and the marketed drug Pexidartinib (PLX3397), we merged fragments of Pexidartinib with our pyrrolo[2,3-*d*]pyrimidine nucleus, and the idea was supported by initial molecular docking studies. Thus, several new compounds were synthesized with Pexidartinib fragments on C4, C5, and C6 on the pyrrolopyrimidine scaffold using molecular hybridization. **Methods**: Nine final products were synthesized using a combination of Buchwald-Hartwig and Suzuki-Miyaura cross-coupling reactions in three to four steps and in good yields. The analogues were subsequently profiled as CSF1R inhibitors in enzymatic and cellular assays, and ADME properties were evaluated for some derivatives. **Results**: *N*-Methyl-*N*-(3-methylbenzyl)-6-(6-((pyridin-3-ylmethyl)amino)pyridin-3-yl)-*7H*-pyrrolo[2,3-*d*]pyrimidin-4-amine (**12b**) emerged as the most potent CSF1R inhibitor, showing low-nanomolar enzymatic activity, cellular efficacy, and favorable ADME properties, highlighting its promise as a lead compound for further development. **Conclusions**: These findings suggest that combining structural elements from previously reported CSF1R inhibitors such as Pexidartinib could guide the development of improved drug candidates targeting this kinase.

## 1. Introduction

The complex network of signaling pathways in the human body plays an immense role in maintaining immune responses, cellular functions, and differentiation. Amid these, the colony-stimulating factor 1 (CSF-1) and the corresponding transmembrane receptor (CSF1R) play a significant role in the pathogenesis and progression of various diseases, including osteoporosis, cancer, and autoimmune disorders [1,2]. The binding of CSF-1 to its receptor (CSF1R) induces receptor dimerization, followed by intracellular autophosphorylation of specific tyrosine residues within the cytoplasmic domain. This post-translational modification initiates the activation of downstream intracellular signaling cascades, causing migration, cell proliferation, and differentiation of cells of the myeloid lineage, exerting substantial influence on numerous physiological processes [2,3,4,5]. Thus, significant research efforts over the past decade have been directed toward the development of CSF1R inhibitors [6]. CSF1R belongs to the CSF-1/PDGF receptor family that comprises numerous valuable members, including platelet-derived growth factor receptor (PDGFR) *α* and *β*, FMS-like tyrosine kinase 3 (FLT-3), and stem cell factor receptor (KIT).

**Figure 1:**
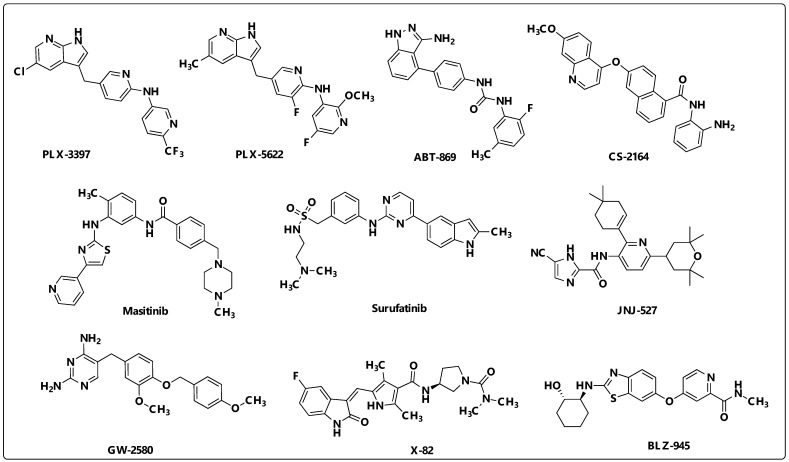
Chemical structures of CSF1R inhibitors approved for market and in clinical trials.

To date, only three small molecule CSF1R inhibitors (Pexidartinib [7], Vimseltinib [8] and Surufatinib [9]) have been approved (the latter only in China) for the treatment of tenosynovial giant cell tumors and non-pancreatic neuroendocrine tumors, respectively. However, there are several CSF-1R inhibitors under investigation as therapeutic agents for various disease targets, and representative chemical structures from the literature are shown in Figure 1 [10,11,12,13,14,15]. Recent studies suggest that CSF1R inhibitors may reduce amyloid plaque formation in Alzheimer’s disease models. For instance, Pexidartinib has shown efficacy in reducing amyloid deposition in vivo, indicating potential therapeutic utility [16]. Similarly, GW2580 demonstrated comparable effects in preclinical models [17]. Furthermore, the CSF1R inhibitor JNJ-527 has completed Phase 1 clinical trials for Alzheimer’s disease, although subsequent trial details remain unpublished [18]. Together, these findings indicate that selective CSF1R inhibition could offer therapeutic benefits through modulation of neuroinflammation and amyloid pathology in Alzheimer’s disease. In addition, several recently discovered CSF1R kinase inhibitors, such as PLX5622, BLZ945, ABT869, CS2164, and X82 (Figure 1), are currently in preclinical stages and exhibit varying selectivity profiles. However, a significant limitation of most existing CSF1R inhibitors is their lack of specificity, as they often target other kinases in addition to CSF1R. This off-target activity complicates the interpretation of their therapeutic effects and makes it challenging to directly attribute their efficacy solely to CSF1R inhibition [10,19,20,21,22]. Given these challenges, there remains an urgent need to discover novel CSF1R inhibitors featuring unique chemical scaffolds and enhanced selectivity, offering significant potential for the development of precise and effective therapeutic agents targeting CSF1R-mediated pathways across diverse disease states.

Heterocycles have achieved a significant mark in developing potent CSF1R inhibitors, and are obtained by various synthetic approaches. Significant research on pyrrolo[2,3-*d*]pyrimidine scaffold has been conducted in our lab to generate novel and potent CSF1R inhibitors, and we have reported several leads with excellent inhibitory activity and selectivity profiles, but with other minor shortcomings [23,24,25,26].

Molecular hybridization involves the rational combination of pharmacophoric elements from two or more bioactive molecules to create a hybrid compound with enhanced biological activity [27]. This approach has been particularly successful in kinase inhibitor development, where the integration of structural features from distinct inhibitors has yielded compounds with dual or multitarget activity [28]. In contrast, scaffold hopping, primarily employed in in silico compound screening, aims to replace the core structure of a known kinase inhibitor with a different scaffold while retaining or improving its binding affinity and selectivity [29].

**Figure 2:**
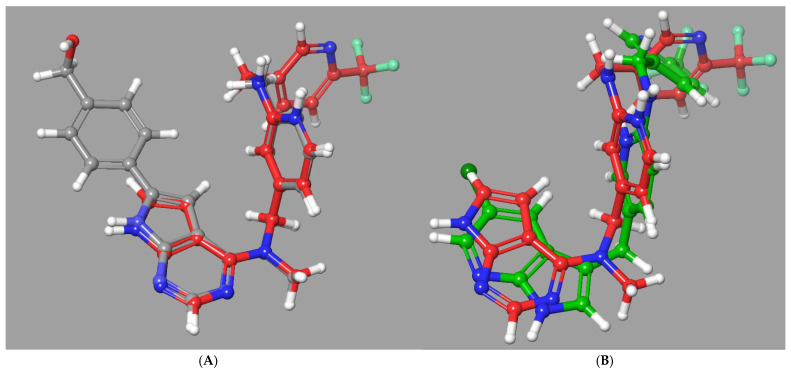
(**A**) The docked conformation of the proposed hybrid molecule (ligand colored in red) exhibits substantial overlap with the ligand (ligand colored in gray) from the published crystal structure (PDB ID: 8CGC). The protein has been omitted for clarity. (**B**) The docking pose of the proposed hybrid molecule (ligand colored in red) shows a reasonable structural overlap with the corresponding docked pose of Pexidartinib (ligand colored in green).

CSF1R is a validated target for computational docking [30], and initial in silico docking studies using Glide (Schrödinger Release 2025-2) [31] suggested that incorporating the dipyridine moiety from pexidartinib at position six of the pyrrolopyrimidine scaffold (compound **4c**) results in a favorable alignment of the core with our previously reported crystal structure (Figure 2A). Furthermore, docking Pexidartinib into the same protein structure (PDB: 8CGC) [23] demonstrates a reasonably good spatial overlap between the pyrrolopyridine core of Pexidartinib and the pyrrolopyrimidine core in the suggested hybrid derivative (Figure 2B).

**Figure 3:**
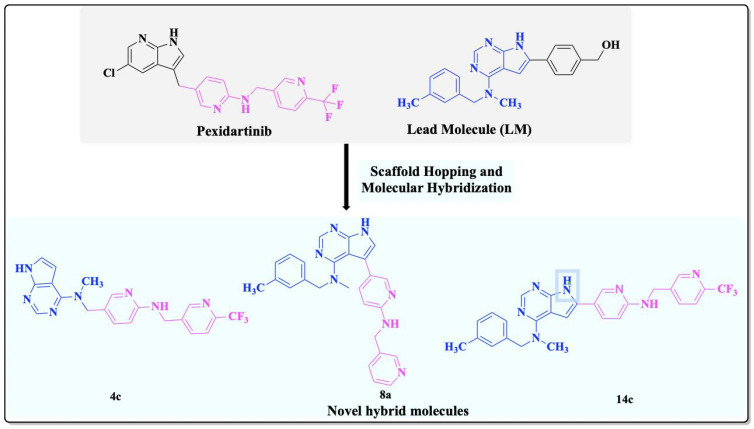
Design strategy towards novel hybrids as CSF1R inhibitors.

With this in mind, we aimed to optimize our existing lead compounds by merging the pyrrolopyrimidine core from our previously developed inhibitors with structural features inspired by the commercial drug Pexidartinib, as illustrated in Figure 3. We aimed for the synthesis of nine analogues as potential new CSF1R inhibitors, incorporating structural elements from Pexidartinib in positions four, five, and six on the pyrrolopyrimidine scaffold. These analogues were subsequently profiled as CSF1R inhibitors in enzymatic and cellular assays, and the results are reported herein.

## 2. Results and Discussion

### 2.1. Chemistry

The chemistry for the target compounds was entirely dependent on the substituent type and positioning of substituents on the pyrrolo[2,3-*d*]pyrimidine nucleus.

**Scheme 1:**
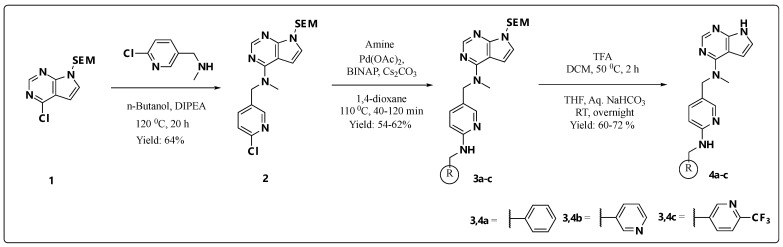
Design and synthesis of C-4 substituted pyrrolo[2,3-*d*]pyrimidines as CSF1R inhibitors.

Scheme 1 illustrates the synthesis of C-4 substituted pyrrolo[2,3-*d*]pyrimidines **4a**–**c** via a scaffold-hopping strategy, incorporating the dipyridine moiety from Pexidartinib with three different aryl units. The two-step synthesis of the SEM-protected pyrrolopyrimidine (**1**) has been previously reported by us [22]. Amination of compound **1** with 1-(6-chloropyridin-3-yl)-*N*-methylmethanamine in the presence of DIPEA as a base under heating conditions in *n*-BuOH yielded key intermediate **2** in 64% yield. Subsequently, the SEM-protected precursors **3a**–**c** were synthesized via the Buchwald–Hartwig cross-coupling between key intermediate **2** and the corresponding amine, using Pd(OAc)_2_ as a catalyst, BINAP as a ligand, and Cs_2_CO_3_ in dioxane at 110 °C. Finally, SEM deprotection of **3a**–**c** using trifluoroacetic acid (TFA), followed by neutralization with aqueous NaHCO_3_, afforded the desired final hybrids **4a**–**c** in good yields.

**Scheme 2:**
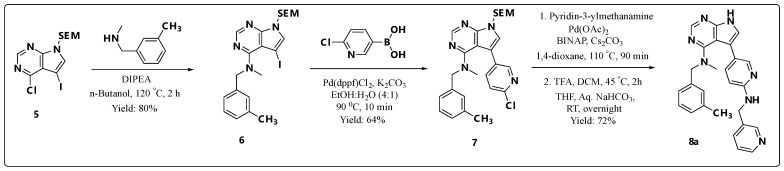
Synthesis of C-5 substituted pyrrolo[2,3-*d*]pyrimidine.

We further aimed to extend the inhibitor collection by incorporating the Pexidartinib flanking group featuring pyridine termini at the C-5 position of the pyrrolo[2,3-*d*]pyrimidine scaffold while preserving the C-4 substitution present in the lead molecule (LM), as shown in Scheme 2. The two-step synthesis of the SEM-protected iodinated pyrrolopyrimidine **5** has been previously reported by our group [32]. Amination at the C-4 position of **5** with *N*-methyl-1-(*meta*-tolyl)methanamine, using DIPEA as a base under thermal conditions (120 °C for 2 h), furnished compound **6** at an 80% yield. The chloropyridine moiety was introduced at the C-5 position of the core scaffold via a Suzuki–Miyaura cross-coupling, employing Pd(dppf)_2_Cl_2_ as a catalyst inEtOH/H_2_O (vol./vol 4:1), affording key intermediate **7** in 64% yield. Subsequent Buchwald–Hartwig amination of **7** with 3-picolylamine at 110 °C for 90 min., as outlined in Scheme 2, furnished the SEM-protected precursor. Deprotection of the SEM group gave the final hybrid **8a** with a 72% yield in the final step.

**Scheme 3:**
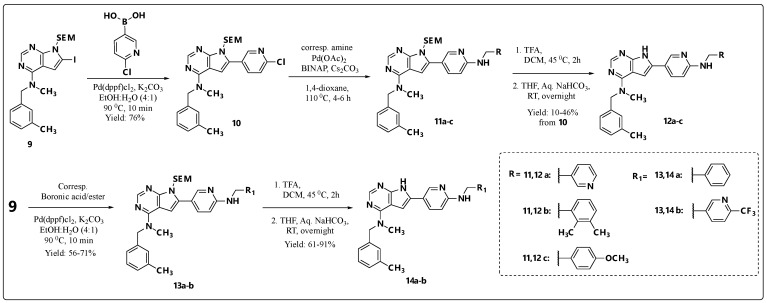
Synthesis of C-6 substituted pyrrolo[2,3-*d*]pyrimidines as CSF1R inhibitors.

Finally, the C-6 substituted pyrrolo[2,3-*d*]pyrimidines **12b**,**d**,**e** and **14a**,**c** (Scheme 3) were synthesized, incorporating a 3-pyridyl unit flanked by five different arylamines in the *para* position. The synthesis of SEM-protected iodinated intermediate **9** has previously been described [23]. Two synthetic routes were evaluated (Scheme 3). Initially, a two-step procedure was employed, wherein the 6-chloro-3-pyridyl moiety was introduced via a Suzuki–Miyaura cross-coupling catalyzed by Pd(dppf)_2_Cl_2_ in a EtOH/H_2_O (4:1) solvent system at 90 °C for 10 min, affording the key C-6 substituted intermediate **10**. This intermediate then underwent Buchwald–Hartwig amination with three amines to generate SEM-protected intermediates **11b**, **11d**, and **11e**, which were used directly in subsequent steps without further purification. Final SEM deprotection of intermediates **11b**, **11d**, and **11e** was achieved using TFA, followed by neutralization with aqueous NaHCO_3_, yielding the C-6 substituted final products **12b**, **12d**, and **12e**. For the remaining derivatives, the corresponding boronic esters were commercially available. Accordingly, the pyridyl flanking group was introduced in a single step via a Suzuki–Miyaura cross-coupling reaction, as previously described. Subsequent SEM deprotection of derivatives **13a** and **13c**, following the same procedure, afforded the corresponding analogs **14a** and **14c**.

### 2.2. Enzymatic and Cellular Assays

**Figure 4:**
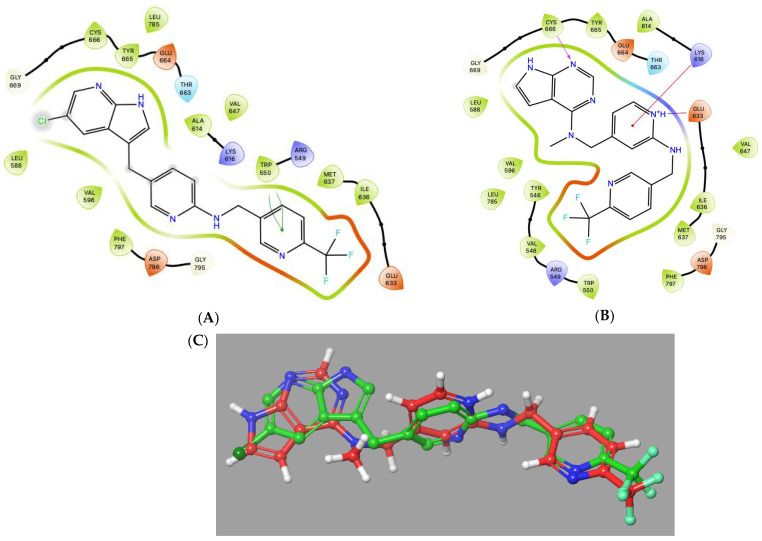
Ligand interaction diagrams for (**A**) the crystal structure of Pexidartinib (PDB ID: 4R7H) and (**B**) the docked conformation of compound **4c** at the same binding site. (**C**) Superimposition of Pexidartinib (green) from the 4R7H crystal structure with the docked pose of **4c** (red). For clarity, the protein has been omitted from the visualization.

The inhibitor candidates were first assayed for their CSF1R enzymatic inhibitory properties, and as can be observed in Table 1, compounds with substitution on position 4 and 5 of the pyrrolopyrimidine scaffold were surprisingly completely inactive towards this kinase. However, substitutions at position 6 of the scaffold afforded compounds with potent inhibitory activity against the CSF1R kinase, exhibiting IC_50_ values in the low nanomolar range, comparable to the reference inhibitor Pexidartinib. The reason for the lack of activity observed for compounds **4a**–**c**, despite initially promising in silico docking results, remains unclear. It may be attributed to the crystal structure used for docking studies, representing a conformation that is not prevalent in the cellular environment. We therefore repeated the docking studies using the CSF1R crystal structure co-crystallized with Pexidartinib (PDB: 4R7H) [7], but failed to reproduce the initial docking outcomes using PDB: 8CGC. In contrast, docking studies of the active derivatives into the CSF1R crystal structure (PDB: 4R7H) revealed ligand–protein interactions similar to those observed for Pexidartinib, as exemplified in Figure 4.

KIT and CSF1R are structurally related receptor tyrosine kinases with conserved ATP-binding pockets. Therefore, inhibitors targeting CSF1R frequently exhibit off-target activity against KIT, as can be seen for Pexdartinib in Table 1. However, the majority of the developed inhibitors demonstrate negligible or no activity toward KIT in this assay.

Given the promising enzymatic activity of the synthesized derivatives, cellular assays were performed using Ba/F3 cells engineered to depend on constitutively active CSF1R. Compound **4a** showed relatively high cellular activity; however, a comparable effect was observed in the presence of IL-3, indicating that the activity was primarily due to cytotoxicity. In contrast, compound **12b**, despite its low-nanomolar enzymatic potency, demonstrated only modest cellular activity. Compound **14c**, with an enzymatic IC_50_ of 7.18 nM, was completely inactive in cells, likely due to poor solubility and limited permeability (Table 2). We have also previously observed a low correlation between enzymatic and cellular activities [23,24,25,26], which may arise from the enzymatic protein construct not accurately mimicking the active conformation of CSF1R in a cellular context.

### 2.3. ADME Profiling of Selected Compounds

Six selected compounds were assessed for metabolic stability (clearance) in mouse liver microsomes (MLM) and human liver microsomes (HLM), and apparent permeability (P_app_) using a MDCK permeability assay. The results are compared with those of PLX3397 in Table 2. Despite exhibiting favorable solubility, compounds **4a**–**4c** and **8a** are of limited interest for further development due to their low biological activity. Notably, the unsubstituted pyridine scaffold appears to represent a metabolic soft spot in compounds **4b** and **8a**, as indicated by their instability in human and mouse liver microsomes (HLM and MLM, respectively). Among the synthesized compounds with some cellular activity, **12b** exhibited the highest metabolic stability in both mouse and human liver microsomes (MLM: 128.4 µL/min/mg; HLM: 115.5 µL/min/mg). Although currently limited by solubility and permeability, its metabolic profile provides a starting point for targeted structural modifications aimed at overcoming these challenges. Improving its physicochemical properties may significantly enhance the pharmacokinetic potential of analogues as viable candidates.

### 2.4. Kinase Selectivity

**Figure 5:**
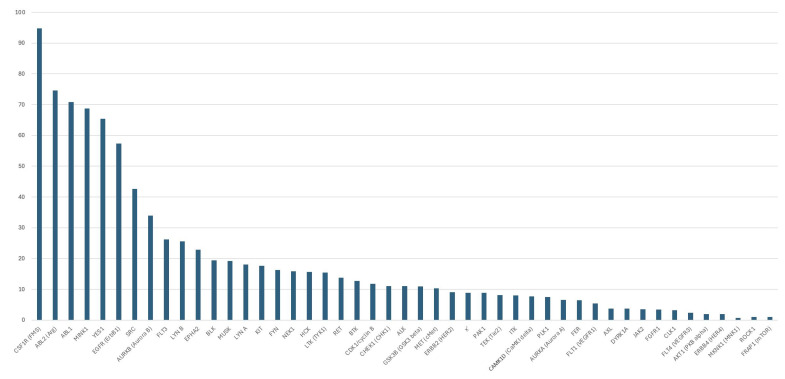
Kinase inhibition profile of **14c** at 1 μM test concentration.

To further evaluate potential off-target activity, compound **14c** was screened against a panel of 50 kinases at a concentration of 1 μM. The results demonstrated high selectivity of compound **14c** towards CSF1R (Figure 5). Most notably, inhibition of the closely related kinases FLT3, KIT, and PDGFRβ was minimal, with inhibition levels of 26%, 18%, and 8%, respectively. Inhibitor selectivity can be quantified using the selectivity score (S-score), which assumes that low inhibitory activity is clinically irrelevant [33]. The S-score is calculated by dividing the number of kinases inhibited above a defined threshold by the total number of kinases tested. A non-selective inhibitor approaches an S-score of 1, whereas a highly selective inhibitor approaches 0. Using a 50% inhibition threshold, compound **14c** has an S-score of 0.12. Further details on the selectivity data can be found in SI (Table S1).

## 3. Materials and Methods

### 3.1. Chemicals and Data Analysis

Most of the chemicals, reagents, starting materials and solvents were purchased from Merck (Darmstadt, Germany), Apollo Scientific (Stockport, UK) and TCI Chemicals (Tokyo, Japan). These were used without further purification. Solvents were dried over molecular sieves for 24 h, or dry solvents were collected from a Braun MB SPS-800 solvent purification system (Braun, Frankfurt am Main, Germany). Reactions sensitive to moisture or oxygen were conducted under a N_2_ atmosphere using oven-dried glassware. Thin-layer chromatography (TLC) with silica-gel on aluminum plates, F254, Merck, was used to monitor the chemical reactions. Purification of compounds by flash column chromatography was performed on pre-packaged silica-gel cartridges obtained from Interchim (PuriFlash cartridges) or with silica-gel (40–63 mesh, 60 Å) using standard glassware. NMR spectra were recorded using the Bruker Avance III HD 400 or 600 MHz instrument in either CDCl_3_ containing tetramethyl silane or DMSO-*d*_6_ as solvents. ^1^H and ^13^C chemical shifts are reported in parts per million (ppm) using tetramethylsilane (0.00 ppm) or solvent (DMSO-*d*_6_, 2.50/39.52 ppm) as reference, and coupling constants (*J*) are measured in hertz (Hz). Accurate mass determination was performed on a Synapt G2-Q-TOF instrument from Waters TM in either a positive or negative mode. The samples were ionized with an ASAP (APCI) or ESI probe. Exact mass calculations and spectra processing were done using Waters TM Software Masslynx V4.1 SCN871. The purity of the final inhibitors was assessed on HPLC. The HPLC method was as follows: Waters Acquity UPLC system, a Waters Acquity BEH C18 (length 50 mm, ID 2.1 mm, particle size 1.7 μm) column, running at a flow rate of 0.5 mL/min. A gradient elution using MeCN/H_2_O as mobile phase was performed as follows: 5% MeCN for 0.5 min, then a linear gradient up to 95% MeCN over 7.5 min, and finally 95% MeCN for 1.5 min. The column was kept at a temperature of 60 °C. For each run, 5 µL of a 200 µM solution of the analyte dissolved in MeOH/H_2_O (75:25 vol%) was injected. Waters MassLynx 4.1 or Agilent Chemstation was used as software.

### 3.2. Synthesis of Building Blocks

#### 3.2.1. *N*-((6-Chloropyridin-3-yl)methyl)-*N*-methyl-7-((2-(trimethylsilyl)ethoxy)methyl)-7*H*-pyrrolo[2,3-*d*]pyrimidin-4-amine (**2**)

Dry *n*-BuOH (5 mL) was added to a round-bottom flask (RB), followed by the addition of 4-chloro-7-((2-(trimethylsilyl)ethoxy)methyl)-7*H*-pyrrolo[2,3-*d*]pyrimidine (150 mg, 0.63 mmol) (**1**) and DIPEA (0.32 mL, 1.89 mmol). The reaction mixture was stirred for 10 min under an inert atmosphere before the slow addition of 1-(6-chloropyridin-3-yl)-*N*-methylmethanamine (118 mg, 0.76 mmol) dissolved in *n*-BuOH (10 mL). The mixture was then heated at 120 °C for 20 h. Upon completion, the solvent was evaporated using a rotary evaporator, and the residue was partitioned between water (25 mL) and EtOAc (25 mL). The organic layer was separated, dried over anhydrous Na_2_SO_4_, and concentrated under reduced pressure. Purification by silica-gel flash column chromatography (EtOAc/*n*-pentane, 3:2, Rf = 0.56) gave 136 mg (0.336 mmol, 64%) of a brown viscous liquid. ^1^H NMR (400 MHz, DMSO-*d*_6_) δ: 8.36 (d, *J* = 2.5 Hz, 1H), 8.20 (s, 1H), 7.72 (dd, J = 8.2, 2.5 Hz, 1H), 7.45 (d, *J* = 8.2 Hz, 1H), 7.33 (d, *J* = 3.7 Hz, 1H), 6.66 (d, *J* = 3.7 Hz, 1H), 5.51 (s, 2H), 5.01 (s, 2H), 3.49 (t, *J* = 7.9 Hz, 2H), 3.37 (s, 3H), 0.81 (t, *J* = 8.1 Hz, 2H), −0.09 (s, 9H); ^13^C NMR (101 MHz, DMSO-*d*_6_) δ: 156.5, 151.2, 151.0, 148.89, 148.86, 138.6, 133.6, 124.5, 124.1, 102.1, 102.0, 72.2, 65.3, 49.8, 37.4, 17.1, −1.4 (3C). HRMS (ES+, *m*/*z*): found 404.1674, calculated for C_19_H_27_ClN_5_OSi, [M + H]^+^, 404.1673.

#### 3.2.2. General Procedure A: Synthesis of **3a**–**c** and **11a–c** via Buchwald–Hartwig Amination Reaction

1,4-Dioxane (10 mL), *N*-((6-chloropyridin-3-yl)methyl)-*N*-methyl-7-((2-(trimethylsilyl)ethoxy)methyl)-7*H*-pyrrolo[2,3-*d*]pyrimidin-4-amine (**2**), or 6-(6-chloropyridin-3-yl)-*N*-methyl-*N*-(3-methylbenzyl)-7-((2-(trimethylsilyl)ethoxy)methyl)-7*H*-pyrrolo[2,3-*d*]pyrimidin-4-amine (**10**) (1 equiv.) was added to a round bottom flask (RB), followed by the corresponding amine (1.5 equiv.) and cesium carbonate (3 equiv.). The reaction mixture was degassed for 10 min before the addition of Pd(OAc)_2_ (0.1 equiv.) and BINAP (0.1 equiv.). The mixture was then heated at 100 °C for 0.33–6 h. Upon completion, the solvent was evaporated using a rotary evaporator, and the residue was diluted with water (30 mL) and extracted with EtOAc (3 × 20 mL). The combined organic layers were washed with brine (30 mL), dried over anhydrous Na_2_SO_4_, and concentrated under reduced pressure. The crude product was purified by silica gel column chromatography, as specified for each compound.

#### 3.2.3. *N*-((6-(Benzylamino)pyridin-3-yl)methyl)-*N*-methyl-7-((2-(trimethylsilyl)ethoxy)methyl)-7*H*-pyrrolo[2,3-*d*]pyrimidin-4-amine (**3a**)

The compound was synthesized following general procedure A, using *N*-((6-chloropyridin-3-yl)methyl)-*N*-methyl-7-((2-(trimethylsilyl)ethoxy)methyl)-7*H*-pyrrolo[2,3-*d*]pyrimidin-4-amine (0.20 g, 0.50 mmol) and benzylamine (0.082 mL, 0.74 mmol). Purification by silica gel flash column chromatography (EtOAc/*n*-pentane, 8:2, Rf = 0.40) yielded the desired product as a brown viscous liquid (128 mg, 0.269 mmol, 54% yield). ^1^H NMR (400 MHz, DMSO-*d*_6_) *δ*: 8.25 (s, 1H), 7.97 (d, *J* = 2.3 Hz, 1H), 7.36–7.31 (m, 6H), 7.26–7.22 (m, 1H), 7.06 (t, *J* = 6.0 Hz, 1H), 6.68 (d, *J* = 3.7 Hz, 1H), 6.50 (d, *J* = 8.6 Hz, 1H), 5.55 (s, 2H), 4.85 (s, 2H), 4.48 (d, *J* = 6.0 Hz, 2H), 3.54 (t, *J* = 7.9 Hz, 2H), 3.30 (s, 3H), 0.86 (t, *J* = 8.0 Hz, 2H), −0.04 (s, 9H); ^13^C NMR (101 MHz, DMSO-*d*_6_) *δ*: 158.0, 156.6, 151.2, 151.0, 146.7, 140.6, 136.3, 128.1 (2C), 127.1 (2C), 126.4, 124.1, 120.8, 108.0, 102.1, 102.0, 72.2, 65.3, 49.8, 44.1, 36.6, 17.1, −1.4 (3C).

#### 3.2.4. *N*-Methyl-*N*-((6-((pyridin-2-ylmethyl)amino)pyridin-3-yl)methyl)-7-((2-(trimethylsilyl)ethoxy)methyl)-7*H*-pyrrolo[2,3-*d*]pyrimidin-4-amine (**3b**)

The compound was synthesized following general procedure A, using *N*-((6-chloropyridin-3-yl)methyl)-*N*-methyl-7-((2-(trimethylsilyl)ethoxy)methyl)-7*H*-pyrrolo[2,3-*d*]pyrimidin-4-amine (0.25 g, 0.61 mmol) and pyridin-2-ylmethanamine (0.1 mL, 0.93 mmol. Upon completion, the solvent was evaporated using a rotary evaporator, and the residue was partitioned between water (50 mL) and EtOAc (50 mL). The combined organic layers were washed with brine (30 mL), dried over anhydrous Na_2_SO_4_, and concentrated. The crude product was used directly for SEM deprotection without purification.

#### 3.2.5. *N*-Methyl-*N*-((6-(((6-(trifluoromethyl)pyridin-3-yl)methyl)amino)pyridin-3-yl)methyl)-7-((2-(trimethylsilyl)ethoxy)methyl)-7*H*-pyrrolo[2,3-*d*]pyrimidin-4-amine (**3c**)

The compound was synthesized following general procedure A, using *N*-((6-chloropyridin-3-yl)methyl)-*N*-methyl-7-((2-(trimethylsilyl)ethoxy)methyl)-7*H*-pyrrolo[2,3-*d*]pyrimidin-4-amine (0.25 g, 0.61 mmol) and (6-(trifluoromethyl)pyridin-3-yl)methanamine (0.13 mL, 0.93 mmol). Purification by silica-gel flash column chromatography (EtoAc/*n*-pentane, 8:2, Rf = 0.56) gave the desired product with 62% yield (210 mg, 0.386 mmol) as brown viscous liquid. ^1^H NMR (400 MHz, DMSO-*d*_6_) *δ*: 8.70 (s, 1H), 8.19 (s, 1H), 7.95 (dd, *J* = 8.1, 2.1 Hz, 1H), 7.91 (d, *J* = 2.3 Hz, 1H), 7.82 (d, *J* = 8.1 Hz, 1H), 7.33 (dd, *J* = 8.6, 2.4 Hz, 1H), 7.30 (d, *J* = 3.7 Hz, 1H), 7.21 (t, *J* = 6.0 Hz, 1H), 6.63 (d, *J* = 3.7 Hz, 1H), 6.51 (d, *J* = 8.5 Hz, 1H), 5.50 (s, 2H), 4.80 (s, 2H), 4.56 (d, *J* = 5.9 Hz, 2H), 3.49 (t, *J* = 7.8 Hz, 2H), 3.25 (s, 3H), 3.49 (t, *J* = 8.2 Hz, 2H), −0.09 (s, 9H); ^13^C NMR (101 MHz, DMSO-*d*_6_) *δ*: 157.5, 156.6, 151.2, 151.0, 149.2, 146.6, 144.6 (q, *J* = 33.7 Hz)*, 140.5, 136.7, 136.6, 124.2, 121.8 (q, *J* = 274.7 Hz) *, 121.5, 120.3 (q, *J* = 2.9 Hz)*, 108.5, 102.1, 102.0, 72.2, 65.3, 49.8, 41.5, 36.7, 17.1, −1.4 (3C). * The low intensity signals are not seen

#### 3.2.6. General Procedure B: Synthesis of Final Compounds **4a**–**c**, **8a** and **14a**,**b** via SEM-Deprotection

To the SEM-protected intermediates (**3a**–**c**, **7**, and **13a**–**b**; 60–120 mg, 1 equiv.), DCM (10 mL) and TFA (2 mL) were added, and the mixture was stirred at 50 °C for 2 h. The reaction mixture was then concentrated in vacuo before being taken up in THF (10 mL) and a 25% aqueous solution of NaHCO_3_ (20 mL), then stirred at room temperature overnight. The solvent was evaporated using a rotary evaporator, and the resulting precipitate was filtered, washed with water (3 × 10 mL) and *n*-pentane (3 × 10 mL), and purified by silica gel column chromatography, yielding the pure products in 60–72%.

#### 3.2.7. *N*-((6-(Benzylamino)pyridin-3-yl)methyl)-*N*-methyl-7*H*-pyrrolo[2,3-*d*]pyrimidin-4-amine (**4a**)

The reaction was run as described in general procedure B using **3a** (100 mg, 0.21 mmol). Purification by silica-gel flash column chromatography (acetone/DCM, 1:1, Rf = 0.49) gave the product (43 mg, 0.124 mmol) with 60% yield; mp. 222–224 °C; HPLC purity: 99.7%; ^1^H NMR (600 MHz, DMSO-*d*_6_) *δ*: 11.64 (s, 1H), 8.13 (s, 1H), 7.92 (d, *J* = 2.3 Hz, 1H), 7.31–7.27 (m, 5H), 7.21–7.18 (m, 1H), 7.11 (m, 1H), 7.00 (t, *J* = 6.0 Hz, 1H), 6.54 (dd, *J* = 3.6, 1.7 Hz, 1H), 6.46 (d, *J* = 8.6 Hz, 1H), 4.79 (s, 2H), 4.44 (d, *J* = 6.0 Hz, 2H), 3.24 (s, 3H); ^13^C NMR (151 MHz, DMSO-*d*_6_) *δ*: 158.0, 156.6, 151.6, 150.7, 146.7, 140.6, 136.4, 128.1 (2C), 127.1 (2C), 126.4, 121.1, 120.7, 108.0, 101.9, 101.4, 49.8, 44.1, 36.6; HRMS (ES+, *m*/*z*): found 345.1828, calculated for C_20_H_21_N_6_, [M + H]^+^, 345.1828.

#### 3.2.8. *N*-Methyl-*N*-((6-((pyridin-2-ylmethyl)amino)pyridin-3-yl)methyl)-7*H*-pyrrolo[2,3-*d*]pyrimidin-4-amine (**4b**)

The reaction was run as described in general procedure B using **3b** (170 mg, 0.36 mmol). Purification by silica-gel flash column chromatography (acetone/DCM, 1:1, Rf = 0.34) gave the desired product (75 mg, 0.217 mmol) with 61% yield; mp. 132–134 °C; HPLC purity: 90.7%; ^1^H NMR (400 MHz, DMSO-*d*_6_) *δ*: 11.68 (s, 1H), 8.54 (dd, *J* = 4.8, 0.9 Hz, 1H), 8.18 (s, 1H), 7.95 (d, *J* = 2.3 Hz, 1H), 7.75 (td, *J* = 7.6, 1.8 Hz, 1H), 7.39–7.33 (m, 1H), 7.34 (d, *J* = 7.8 Hz, 1H), 7.29–7.24 (m, 1H), 7.17–7.13 (m, 2H), 6.60–6.56 (m, 2H), 4.85 (s, 2H), 4.57 (d, *J* = 6.0 Hz, 2H), 3.38 (s, 3H); ^13^C NMR (101 MHz, DMSO-*d*_6_) *δ*: 159.9, 157.8, 156.6, 151.6, 150.7, 148.7, 146.7, 136.5, 136.5, 121.7, 121.3, 120.8, 120.7, 108.2, 101.9, 101.4, 49.8, 46.2, 36.7; HRMS (ES+, *m*/*z*): found 346.1775, calculated for C_19_H_20_N_7_, [M + H]^+^, 346.1780.

#### 3.2.9. *N*-Methyl-*N*-((6-(((6-(trifluoromethyl)pyridin-3-yl)methyl)amino)pyridin-3-yl)methyl)-7*H*-pyrrolo[2,3-*d*]pyrimidin-4-amine (**4c**)

The reaction was run as described in general procedure B using **3c** (200 mg, 0.37 mmol). Purification by silica-gel flash column chromatography (acetone/DCM, 1:1, Rf = 0.56) gave the desired product (110 mg, 0.266 mmol) with 72% yield; mp. 205–207 °C; HPLC purity: 99.5%; ^1^H NMR (400 MHz, DMSO-*d*_6_) *δ*: 11.64 (s, 1H), 8.70 (s, 1H), 8.12 (s, 1H), 7.95 (dd, *J* = 8.2, 2.1 Hz, 1H), 7.91 (d, *J* = 2.3 Hz, 1H), 7.82 (d, *J* = 8.1 Hz, 1H), 7.34 (dd, *J* = 8.6, 2.4 Hz, 1H), 7.22 (t, *J* = 6.0 Hz, 1H), 7.12–7.10 (m, 1H), 6.54–6.51 (m, 2H), 4.79 (s, 2H), 4.56 (d, *J* = 5.9 Hz, 2H), 3.24 (s, 3H); NMR (151 MHz, DMSO-*d*_6_) *δ*: 157.5, 156.6, 151.6, 150.7, 149.3, 146.6, 144.7 (q, *J* = 33.7 Hz), 140.5, 136.7, 136.6, 121.8 (q, *J* = 273.3 Hz), 121.7, 120.8, 120.3 (q, *J* = 2.8 Hz), 108.5, 101.9, 101.4, 49.7, 41.5, 36.7; HRMS (ES+, *m*/*z*): found 414.1655, calculated for C_20_H_19_F_3_N_7_, [M + H]^+^, 414.1654.

#### 3.2.10. 5-Iodo-*N*-methyl-*N*-(3-methylbenzyl)-7-((2-(trimethylsilyl)ethoxy)methyl)-7*H*-pyrrolo[2,3-*d*]pyrimidin-4-amine (**6**)

4-chloro-5-iodo-7-((2-(trimethylsilyl)ethoxy)methyl)-7*H*-pyrrolo[2,3-*d*]pyrimidine (100 mg, 0.24 mmol) and DIPEA (0.12 mL, 0.73 mmol) was added to a round-bottom flask containing *n*-BuOH (5 mL). The mixture was stirred at room temperature for 10 min before the addition of *N*-methyl-1-(*m*-tolyl)methanamine (0.04 mL, 0.32 mmol) via syringe. Stirring was continued at 120 °C for 2 h. Upon completion, the solvent was evaporated, and the residue was diluted with water (30 mL) and extracted with EtOAc (3 × 20 mL). The combined organic layers were dried over anhydrous Na_2_SO_4_ and concentrated. Purification by silica gel flash column chromatography (EtOAc/*n*-pentane, 2:8, Rf = 0.55) afforded the product as a pale-yellow viscous liquid (100 mg, 0.196 mmol, 80% yield). ^1^H NMR (400 MHz, DMSO-*d*_6_) *δ*: 8.29 (s, 1H), 7.78 (s, 1H), 7.19 (t, *J* = 7.8 Hz, 1H), 7.05 (d, *J* = 6.3 Hz, 2H), 7.00 (d, *J* = 7.6 Hz, 1H), 5.52 (s, 2H), 4.82 (s, 2H), 3.52 (t, *J* = 7.9 Hz, 2H), 3.11 (s, 3H), 2.26 (s, 3H), 0.81 (t, *J* = 8.0 Hz, 2H), −0.09 (s, 9H); ^13^C NMR (101 MHz, DMSO-*d*_6_) *δ*: 159.8, 152.3, 150.5, 137.6, 137.4, 132.5, 128.4, 128.2, 127.7, 124.7, 106.0, 72.2, 65.6, 56.6, 53.1, 41.5, 21.0, 17.0, −1.4 (3C).

#### 3.2.11. 5-(6-Chloropyridin-3-yl)-*N*-methyl-*N*-(3-methylbenzyl)-7-((2-(trimethylsilyl)ethoxy)methyl)-7*H*-pyrrolo[2,3-*d*]pyrimidin-4-amine (**7**)

A mixture of EtOH/H_2_O (4:1 by vol, 5 mL) was added to a round-bottom flask, followed by **6** (90 mg, 0.18 mmol), (6-chloropyridin-3-yl)boronic acid (33 mg, 0.21 mmol), and K_2_CO_3_ (73 mg, 0.53 mmol). The reaction mixture was degassed for 45 min before adding Pd(dppf)Cl_2_ (13 mg, 0.018 mmol). The flask was then placed in an oil bath at 90 °C and stirred for 10 min. Upon completion, the solvent was evaporated, and the residue was added to water (25 mL) and extracted with EtOAc (2 × 20 mL). The combined organic layers were dried over with anhydrous Na_2_SO_4_ and concentrated. Purification by silica gel flash column chromatography (EtOAc/*n*-pentane, 2:8, Rf = 0.38) afforded the product as a brown viscous liquid (56 mg, 0.113 mmol, 64% yield). ^1^H NMR (400 MHz, DMSO-*d*_6_) *δ*: 8.54 (d, *J* = 2.4 Hz, 1H), 8.39 (s, 1H), 7.94 (dd, *J* = 8.2, 2.5 Hz, 1H), 7.79 (s, 1H), 7.48 (d, *J* = 8.2 Hz, 1H), 7.15 (t, *J* = 7.5 Hz, 1H), 7.04 (d, *J* = 7.6 Hz, 1H), 6.94 (s, 1H), 6.88 (d, *J* = 7.6 Hz, 1H), 5.61 (s, 2H), 4.48 (s, 2H), 3.59 (t, *J* = 7.8 Hz, 2H), 2.57 (s, 3H), 2.24 (s, 3H), 0.85 (t, *J* = 8.0 Hz, 2H), −0.08 (s, 9H); ^13^C NMR (101 MHz, DMSO-*d*_6_) *δ*: 159.8, 152.8, 150.8, 148.4, 148.0, 138.9, 137.4, 137.2, 130.7, 128.4 (2C), 128.2, 127.7, 125.9, 124.9, 123.8, 111.5, 101.8, 72.5, 65.7, 54.9, 20.9, 17.1, −1.4 (3C).

#### 3.2.12. *N*-Methyl-*N*-(3-methylbenzyl)-5-(6-((pyridin-3-ylmethyl)amino)pyridin-3-yl)-7*H*-pyrrolo[2,3-*d*]pyrimidin-4-amine (**8a**)

Compound **7** (0.2 g, 0.41 mmol), pyridin-3-ylmethanamine (0.06 mL, 0.61 mmol), and cesium carbonate (0.4 g, 1.22 mmol) were added to a round-bottom flask containing 1,4-dioxane. The reaction mixture was degassed for 10 min before adding Pd(OAc)_2_ (9 mg, 0.04 mmol) and BINAP (25 mg, 0.04 mmol). The mixture was then heated at 110 °C for 90 min. Upon completion, the solvent was evaporated using a rotary evaporator, and the residue was added to water (25 mL) and extracted with EtOAc (2 × 20 mL). The combined organic layers were dried over anhydrous Na_2_SO_4_ and concentrated. The crude product (160 mg) was used directly for SEM deprotection without further purification, following general procedure B. Purification by silica gel flash column chromatography (MeOH/DCM, 0.5:9.5, Rf = 0.18) afforded the product as a white powder (88 mg, 0.203 mmol, 72% yield); mp. 101–103 °C; HPLC purity: 96.5%; ^1^H NMR (400 MHz, DMSO-*d*_6_) *δ*: 11.94 (s, 1H), 8.56 (d, *J* = 2.2 Hz, 1H), 8.43 (dd, *J* = 4.8, 1.7 Hz, 1H), 8.24 (s, 1H), 8.11 (d, *J* = 2.4 Hz, 1H), 7.71 (d, *J* = 7.8 Hz, 1H), 7.50 (dd, *J* = 8.6, 2.4 Hz, 1H), 7.32–7.29 (m, 2H), 7.16–7.11 (m, 2H), 7.02 (d, *J* = 7.5 Hz, 1H), 6.96 (s, 1H), 6.89 (d, *J* = 7.6 Hz, 1H), 6.53 (d, *J* = 8.5 Hz, 1H), 4.52 (s, 2H), 4.50 (s, 2H), 2.57 (s, 3H), 2.25 (s, 3H); ^13^C NMR (101 MHz, DMSO-*d*_6_) *δ*: 159.8, 156.9, 152.9, 149.9, 148.8, 147.8, 146.2, 137.7, 137.38, 137.30, 136.0, 135.0, 128.4, 128.1, 127.6, 124.9, 123.3, 120.6, 120.6, 113.3, 107.8, 102.1, 54.7, 45.6, 41.9, 21.0; HRMS (ES+, *m*/*z*): found 436.2245, calculated for C_26_H_26_N_7_, [M + H]^+^, 436.2250.

#### 3.2.13. 6-(6-Chloropyridin-3-yl)-*N*-methyl-*N*-(3-methylbenzyl)-7-((2-(trimethylsilyl) ethoxy)methyl)-7*H*-pyrrolo[2,3-*d*]pyrimidin-4-amine (**10**)

Compound **9** (200 mg, 0.39 mmol), (6-chloropyridin-3-yl)boronic acid (74 mg, 0.47 mmol), and K_2_CO_3_ (163 mg, 1.18 mmol) were added to a round-bottom flask with EtOH/H_2_O (3:1 by vol, 8 mL). The reaction mixture was degassed for 45 min before PdCl_2_(dppf) (29 mg, 0.04 mmol) was added. The flask was immediately transferred to an oil bath set at 90 °C and stirred for 5 min. Upon completion, the reaction vessel was removed from the oil bath, allowed to cool for 5 min, and the solvent was evaporated using a rotary evaporator. The residue was partitioned between water (30 mL) and DCM (30 mL), and the organic layer was separated. The water phase was extracted with more DCM (2×30 mL). The combined organic layers were washed with brine (20 mL), dried over anhydrous Na_2_SO_4_, and concentrated. Purification by silica gel flash column chromatography (EtOAc/*n*-pentane, 3:7, Rf = 0.56) afforded the product as a brown viscous liquid (147 mg, 0.297 mmol, 76% yield). ^1^H NMR (400 MHz, DMSO-*d*_6_) *δ*: 8.75 (d, *J* = 2.5 Hz, 1H), 8.27 (s, 1H), 8.21 (dd, *J* = 8.4, 2.6 Hz, 1H), 7.65 (d, *J* = 9.0 Hz, 1H), 7.20 (t, *J* = 7.5 Hz, 1H), 7.08–7.03 (m, 4H), 5.59 (s, 2H), 5.02 (s, 2H), 3.60 (t, *J* = 7.8 Hz, 2H), 3.36 (s, 3H), 2.26 (s, 3H), 0.84 (t, *J* = 8.1 Hz, 2H), −0.10 (s, 9H); ^13^C NMR (101 MHz, DMSO-*d*_6_) *δ*: 156.5, 153.3, 151.7, 149.4, 148.9, 138.8, 138.0, 137.6, 131.4, 128.4, 127.6, 127.5, 127.0, 124.1, 124.0, 104.2, 102.0, 70.2, 65.6, 52.6, 37.4, 21.0, 17.2, −1.5 (3C).

#### 3.2.14. *N*-Methyl-*N*-(3-methylbenzyl)-6-(6-((pyridin-3-ylmethyl)amino)pyridin-3-yl)-7*H*-pyrrolo[2,3-*d*]pyrimidin-4-amine (**12a**)

The compound was prepared following general procedure A, starting with compound **10** (350 mg, 0.71 mmol) and pyridin-3-ylmethanamine (0.1 mL, 1.06 mmol). The reaction mixture was stirred at 110 °C for 6 h. Upon completion, the solvent was evaporated, and the reaction was worked up as previously described. The resulting crude product (**11a**; 180 mg, 0.318 mmol) was used directly for SEM deprotection without further purification, following general procedure B. Purification by silica gel flash column chromatography (MeOH/DCM, 1:9, Rf = 0.28) afforded the desired product **12a** as a white powder (100 mg, 0.229 mmol, 33% yield); mp. 248–250 °C; HPLC purity: 98.1%; ^1^H NMR (400 MHz, DMSO-*d*_6_) *δ*: 11.99 (s, 1H), 8.56 (d, *J* = 2.2 Hz, 1H), 8.47 (d, *J* = 2.4 Hz, 1H), 8.43 (dd, *J* = 4.7, 1.7 Hz, 1H), 8.11 (s, 1H), 7.84 (dd, *J* = 8.8, 2.5 Hz, 1H), 7.72 (d, *J* = 7.9 Hz, 1H), 7.35–7.31 (m, 2H), 7.20 (t, *J* = 7.5 Hz, 1H), 7.07–7.03 (m, 3H), 6.82 (d, *J* = 2.1 Hz, 1H), 6.58 (d, *J* = 8.8 Hz, 1H), 4.98 (s, 2H), 4.53 (d, *J* = 6.0 Hz, 2H), 3.32 (s, 3H), 2.26 (s, 3H); ^13^C NMR (101 MHz, DMSO-*d*_6_) *δ*: 157.5, 156.0, 152.7, 150.5, 148.8, 147.8, 144.2, 138.5, 137.5, 135.9, 135.0, 133.6, 131.8, 128.4, 127.57, 127.54, 124.1, 123.3, 116.4, 108.3, 103.2, 96.3, 52.5, 41.8, 37.2, 21.0; HRMS (ES+, *m*/*z*): found 436.2254, calculated for C_26_H_26_N_7_, [M + H]^+^, 436.2250.

#### 3.2.15. 6-(6-((2,3-Dimethylbenzyl)amino)pyridin-3-yl)-*N*-methyl-*N*-(3-methylbenzyl)-7*H*-pyrrolo[2,3-*d*]pyrimidin-4-amine (**12b**)

The compound was prepared following general procedure A, starting with compound **10** (350 mg, 0.71 mmol) and 2,3-dimethylphenyl)methanamine (100 mg, 0.75 mmol). The reaction mixture was stirred at 110 °C for 6 h. Upon completion, the solvent was evaporated, and the reaction was worked up as previously described. The resulting crude product (**11b**; 60 mg, 0.101 mmol) was used directly for SEM deprotection without further purification, following general procedure B. Purification by silica gel flash column chromatography (MeOH/DCM, 1:9, Rf = 0.50) afforded the desired product **12b** as a white powder (24 mg, 0.051 mmol, 10% yield); mp. 268–270 °C (decomp.); ^1^H NMR (600 MHz, DMSO-*d*_6_) *δ*: 11.21 (s, 1H), 8.75 (d, *J* = 2.5 Hz, 1H), 8.15 (s, 1H), 8.09 (dd, *J* = 8.8, 2.5 Hz, 1H), 7.33 (d, *J* = 0.8 Hz, 1H), 7.20 (t, *J* = 7.5 Hz, 1H), 7.08 (s, 1H), 7.06–7.04 (m, 4H), 6.99–6.97 (m, 1H), 6.93–6.89 (m, 2H), 5.45 (s, 2H), 5.00 (s, 2H), 3.34 (s, 3H), 3.32 (s, 3H), 2.26 (s, 3H), 2.25 (s, 3H); ^13^C NMR (151 MHz, DMSO-*d*_6_) *δ*: 156.3, 155.0, 153.0, 151.1, 144.1, 138.3, 137.5, 136.2, 136.1, 133.7, 133.3, 130.5, 128.4, 127.8 (2C), 127.5, 125.0, 124.1, 123.3, 121.3, 114.2, 103.2, 98.5, 52.5, 37.3, 21.0 (2C), 19.9, 14.2; HRMS (ES+, *m*/*z*): found 463.2613, calculated for C_29_H_31_N_6_, [M + H]^+^, 463.2610.

#### 3.2.16. 6-(6-((4-Methoxybenzyl)amino)pyridin-3-yl)-*N*-methyl-*N*-(3-methylbenzyl)-7*H*-pyrrolo[2,3-*d*]pyrimidin-4-amine (**12c**)

The compound was prepared following general procedure A, starting with compound **10** (250 mg, 0.50 mmol) and (4-methoxyphenyl)methanamine (0.1 mL, 0.75 mmol). The reaction mixture was stirred at 110 °C for 4 h. Upon completion, the solvent was evaporated, and the reaction was worked up as previously described. The resulting crude product (**11c**; 185 mg, 0.311 mmol) was used directly for SEM deprotection without further purification, following general procedure B. Purification by silica gel flash column chromatography (MeOH/DCM, 1:9, Rf = 0.56) afforded the desired product **12c** as a white powder (103 mg, 0.221 mmol, 46% yield); mp. 224–226 °C; HPLC purity: 98.0%; ^1^H NMR (600 MHz, DMSO-*d*_6_) *δ*: 11.98 (s, 1H), 8.46 (d, *J* = 2.5 Hz, 1H), 8.10 (s, 1H), 7.80 (dd, *J* = 8.8, 2.5 Hz, 1H), 7.26 (d, *J* = 8.6 Hz, 2H), 7.21–7.16 (m, 2H), 7.08 (s, 1H), 7.05 (t, *J* = 6.9 Hz, 2H), 6.87 (d, *J* = 8.6 Hz, 2H), 6.81 (d, *J* = 2.2 Hz, 1H), 6.53 (d, *J* = 8.8 Hz, 1H), 4.98 (s, 2H), 4.42 (d, *J* = 5.9 Hz, 2H), 3.72 (s, 3H), 3.32 (s, 3H), 2.26 (s, 3H); ^13^C NMR (151 MHz, DMSO-*d*_6_) *δ*: 158.0, 157.7, 156.0, 152.7, 150.4, 144.3, 138.5, 137.5, 133.5, 132.2, 132.0, 128.5 (2C), 128.4, 127.57, 127.54, 124.1, 116.0, 113.6 (2C), 108.0, 103.2, 96.1, 55.0, 52.5, 43.6, 37.2, 21.0; HRMS (ES+, *m*/*z*): found 465.2405, calculated for C_28_H_29_N_6_O, [M + H]^+^, 465.2403.

#### 3.2.17. 6-(6-(Benzylamino)pyridin-3-yl)-*N*-methyl-*N*-(3-methylbenzyl)-7-((2-(trimethylsilyl)ethoxy)methyl)-7*H*-pyrrolo[2,3-*d*]pyrimidin-4-amine (**13a**)

Compound **9** (200 mg, 0.39 mmol), *N*-benzyl-5-(4,4,5,5-tetramethyl-1,3,2-dioxaborolan-2-yl)pyridin-2-amine (146 mg, 0.47 mmol), and K_2_CO_3_ (163 mg, 1.12 mmol) were added to a round-bottom flask containing EtOH/H_2_O (4:1 by vol, 10 mL). The reaction mixture was degassed for 45 min before adding PdCl_2_(dppf) (29 mg, 0.04 mmol). The flask was immediately transferred to an oil bath set at 80 °C and stirred for 5 min. Upon completion, the reaction vessel was removed from the oil bath, allowed to cool for 5 min, and the solvent was evaporated using a rotary evaporator. The residue was partitioned between water and CH_2_Cl_2_ (3 × 20 mL), and the organic layer was separated. The combined organic layers were washed with brine (20 mL), dried over anhydrous Na_2_SO_4_, and concentrated. Purification by silica gel flash column chromatography (EtOAc/*n*-pentane, 2:3, Rf = 0.44) afforded the product as a brown viscous liquid (158 mg, 0.279 mmol, 71% yield). ^1^H NMR (400 MHz, DMSO-*d*_6_) *δ*: 8.25 (d, *J* = 2.4 Hz, 1H), 8.20 (s, 1H), 7.71 (dd, *J* = 8.8, 2.4 Hz, 1H), 7.38–7.29 (m, 5H), 7.24–7.18 (m, 2H), 7.04 (t, *J* = 9.6 Hz, 3H), 6.66 (s, 1H), 6.60 (d, *J* = 8.7 Hz, 1H), 5.49 (s, 2H), 4.99 (s, 2H), 4.52 (d, *J* = 6.0 Hz, 2H), 3.60 (t, *J* = 8.1 Hz, 2H), 3.32 (s, 3H), 2.26 (s, 3H), 0.84 (t, *J* = 8.2 Hz, 2H), −0.09 (s, 9H); ^13^C NMR (101 MHz, DMSO-*d*_6_) *δ*: 158.2, 156.2, 152.7, 150.8, 147.6, 140.3, 138.2, 137.6, 136.8, 134.6, 128.4, 128.2 (2C), 127.59, 127.51, 127.2 (2C), 126.5, 124.0, 115.5, 107.7, 102.1, 100.6, 70.1, 65.6, 52.6, 44.0, 37.2, 21.0, 17.3, −1.4 (3C).

#### 3.2.18. *N*-Methyl-*N*-(3-methylbenzyl)-6-(6-(((6-(trifluoromethyl)pyridin-3-yl)methyl)amino)pyridin-3-yl)-7-((2-(trimethylsilyl)ethoxy)methyl)-7*H*-pyrrolo[2,3-*d*]pyrimidin-4-amine (**13b**)

Compound **9** (200 mg, 0.39 mmol), 5-(4,4,5,5-tetramethyl-1,3,2-dioxaborolan-2-yl)-*N*-((6-(trifluoromethyl)pyridin-3-yl)methyl)pyridine-2-amine (179 mg, 0.47 mmol), and K_2_CO_3_ (163 mg, 1.12 mmol) were added to a round-bottom flask containing a 4:1 mixture of ethanol/water (10 mL). The reaction mixture was degassed for 45 min before PdCl_2_(dppf) (29 mg, 0.04 mmol) was added. The flask was immediately transferred to an oil bath set at 80 °C and stirred for 10 min. Upon completion, the reaction vessel was removed from the oil bath, allowed to cool for 5 min, and the solvent was evaporated using a rotary evaporator. The residue was partitioned between water and DCM (3 × 20 mL), and the organic layer was separated. The combined organic layers were washed with brine, dried over anhydrous Na_2_SO_4_, and concentrated. Purification by silica gel flash column chromatography (EtOAc/*n*-pentane, 1:1, R_f_ = 0.30) afforded the product as a brown viscous liquid (140 mg, 0.220 mmol, 56% yield). ^1^H NMR (600 MHz, DMSO-*d*_6_) *δ*: 8.75 (s, 1H), 8.25 (d, *J* = 2.5 Hz, 1H), 8.21 (s, 1H), 7.99 (d, *J* = 7.1 Hz, 1H), 7.86 (d, *J* = 0.8 Hz, 1H), 7.75 (dd, *J* = 8.7, 2.4 Hz, 1H), 7.55 (t, *J* = 6.1 Hz, 1H), 7.19 (t, *J* = 7.5 Hz, 1H), 7.07–7.02 (m, 3H), 6.68 (s, 1H), 6.66 (d, *J* = 8.4 Hz, 1H), 5.48 (s, 2H), 4.99 (s, 2H), 4.65 (d, *J* = 6.0 Hz, 2H), 3.58 (t, *J* = 8.1 Hz, 2H), 3.32 (s, 3H), 2.25 (s, 3H), 0.82 (t, *J* = 8.1 Hz, 2H), −0.11 (s, 9H); ^13^C NMR (151 MHz, DMSO-*d*_6_) *δ*: 157.7, 156.2, 152.8, 150.8, 149.3, 147.5, 144.7 (q, *J* = 33.7 Hz), 140.3, 138.2, 137.6, 137.0, 136.7 (2C), 134.4, 128.4, 127.5 (q, *J* = 11.9 Hz) *, 124.0, 121.8 (q, *J* = 273.7 Hz), 120.4 (q, *J* = 2.7 Hz), 116.1, 108.1, 102.1, 100.9, 70.1, 65.6, 52.6, 41.5, 37.2, 21.0, 17.3, −1.4 (3C).* The low intensity signals are not seen.

#### 3.2.19. 6-(6-(Benzylamino)pyridin-3-yl)-*N*-methyl-*N*-(3-methylbenzyl)-7*H*-pyrrolo[2,3-*d*]pyrimidin-4-amine (**14a**)

The reaction was carried out as described in general procedure B using **13a** (140 mg, 0.25 mmol), and the product was isolated as a white powder (98 mg, 0.225 mmol, 91% yield); mp. 240–242 °C; HPLC purity: 99.2%; ^1^H NMR (400 MHz, DMSO-*d*_6_) *δ*: 11.99 (s, 1H), 8.46 (d, *J* = 2.4 Hz, 1H), 8.11 (s, 1H), 7.82 (dd, *J* = 8.8, 2.5 Hz, 1H), 7.34–7.18 (m, 7H), 7.08–7.04 (m, 3H), 6.81 (s, 1H), 6.56 (d, *J* = 8.8 Hz, 1H), 4.98 (s, 2H), 4.51 (d, *J* = 6.0 Hz, 2H), 3.32 (s, 3H), 2.26 (s, 3H); ^13^C NMR (101 MHz, DMSO-*d*_6_) δ: 157.7, 156.0, 152.7, 150.4, 144.3, 140.4, 138.4, 137.5, 133.5, 132.0, 128.3, 128.1 (2C), 127.5, 127.5, 127.1 (2C), 126.5, 124.1, 116.1, 108.0, 103.2, 96.1, 52.5, 44.1, 37.2, 21.0; HRMS (ES+, *m*/*z*): found 435.2298, calculated for C_27_H_27_N_6_, [M + H]^+^, 435.2298.

#### 3.2.20. *N*-Methyl-*N*-(3-methylbenzyl)-6-(6-(((6-(trifluoromethyl)pyridin-3-yl)methyl) amino)pyridin-3-yl)-7*H*-pyrrolo[2,3-*d*]pyrimidin-4-amine (**14b**)

The reaction was carried out as described in general procedure B using **13b** (110 mg, 0.17 mmol). The solvent was evaporated using a rotary evaporator and the resulting precipitate was filtered and washed multiple times with water and *n*-pentane. Purification by silica gel flash column chromatography (MeOH/DCM, 1:9, Rf = 0.60) afforded the product as a white powder (53 mg, 0.105 mmol, 61% yield); mp. 244–246 °C; HPLC purity: 99.7%; ^1^H NMR (600 MHz, DMSO-*d*_6_) *δ*: 12.01 (s, 1H), 8.74 (s, 1H), 8.46 (d, *J* = 2.4 Hz, 1H), 8.11 (s, 1H), 7.98 (d, *J* = 7.7 Hz, 1H), 7.87–7.84 (m, 2H), 7.45 (t, *J* = 6.1 Hz, 1H), 7.20 (t, *J* = 7.5 Hz, 1H), 7.07 (s, 1H), 7.04 (t, *J* = 7.0 Hz, 2H), 6.84 (s, 1H), 6.62 (d, *J* = 8.7 Hz, 1H), 4.98 (s, 2H), 4.64 (d, *J* = 6.0 Hz, 2H), 3.32 (s, 3H), 2.26 (s, 3H); ^13^C NMR (151 MHz, DMSO-*d*_6_) δ: 157.2, 156.0, 152.7, 150.5, 149.3, 144.7 (q, *J* = 33.7 Hz), 144.2, 140.4, 138.4, 137.5, 136.7 (2C), 133.7, 131.7, 128.4, 127.5 (q, *J* = 5.0 Hz) *, 124.1, 121.8 (q, *J* = 273.7 Hz), 120.4 (q, *J* = 2.8 Hz), 116.7, 108.5, 103.2, 96.5, 52.5, 41.5, 37.2, 21.0. * The low intensity signals are not seen.

### 3.3. Bio-Chemical Assays

#### 3.3.1. CSF1R Enzymatic Inhibitory Assay (LANCE)

The TR-FRET-based LANCE Ultra assay (Perkin Elmer, Waltham, MA, USA) was used to determine IC_50_ values for various CSF1R inhibitors. Kinase activity and inhibition in this assay was measured as previously reported [26]. Scigilian Analyze software version 5.8.5.15 (Scigilian, Montreal, QC, Canada) was used.

#### 3.3.2. Kinase Panel

The compounds were provided as 10 mM solutions in DMSO, and their enzymatic kinase inhibition potency was assessed as previously reported [26].

#### 3.3.3. KIT-WT Lantha Assay (Kd)

The TR-FRET-based LanthaScreen Binding Assay was performed to determine Kd values of synthesized derivatives against cKIT.

For each sample, 2 µL of assay buffer (50 mM HEPES pH 7.5, 10 mM MgCl_2_, 1 mM EGTA, 0.01% Brij35) was dispensed into an assay plate (e.g., Corning #4513; Corning, NY, USA). Compounds were added at concentrations ranging from 1 µM to 0.00025 µM using an acoustic dispenser (Echo520, Beckman Coulter, Brea, CA, USA) with Echo Dose Response software (software version 2.4.15). Subsequently, 8 µL of kinase anti-GST antibody mixture was added, followed by incubation at 4 °C for 30 min in a pre-cooled, humidified box to minimize edge effects. The reaction was initiated by adding 5 µL of tracer working solution and mixed on a Bioshake 5000 microplate shaker (Q Instruments, Jena, Germany). After another incubation period of 60 min at 4 °C, FRET signals were measured (excitation at 340 nm; emission at 665 nm and 615 nm) using a Pherastar FSX microplate reader (BMG Labtech, Ortenberg, Germany) with 60 µs delay and 200 µs integration. Kd values were calculated from sigmoidal dose-response curves using Scigilian Analyze software version 5.8.5.15 (Scigilian, Montreal, QC, Canada).

#### 3.3.4. Cell Viability Assay with Ba/F3-hCSF1R Cells

Performed as reported previously [23].

#### 3.3.5. ADME Properties

##### Kinetic Solubility and Microsomal Stability Phase I

The aqueous solubility of the compounds and metabolic stability under oxidative conditions was performed as described previously [23].

## 4. Conclusions

The pyrrolo[2,3-*d*]pyrimidine derivative **12b** demonstrated potent low-nanomolar inhibition of CSF1R with acceptable ADME properties. These findings validate molecular hybridization with known inhibitors such as Pexidartinib as a promising approach for developing selective and potent kinase inhibitors.

## Figures and Tables

**Table 1:** Enzymatic IC_50_ values and profiling in Ba/F3 cells.

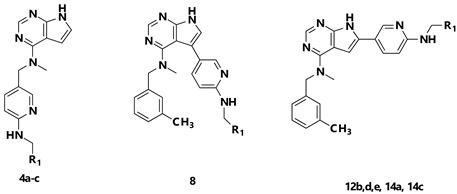					
Compound	R_1_	Enzymatic Assay		Cellular Assay	
		CSF1R (IC_50_, nM) ^1^	KIT (Kd, nM) ^2^	IC_50_ (μM), Ba/F3 CSF1R ^3^	IC_50_ (μM), Ba/F3 IL-3 ^4^
**4a**	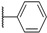	857 (0.96)	>1000 (0.94)	1.16 ± 0.22	1.39 ± 0.40
**4b**	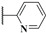	>1000 (0.86)	>1000 (0.97)	>10	>10
**4c**	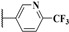	>1000 (0.81)	>1000 (0.99)	9.96 ± 0.02	>10
**8**	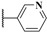	>1000 (0.81)	397 (0.98)	8.46 ± 0.89	>10
**12b**	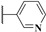	1.54 (0.97)	375 (0.98)	1.15 ± 0.36	>10
**12d**	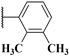	>1000 (0.47)	>1000 (-)	ND	ND ^5^
**12e**	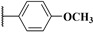	10.50 (0.91)	>1000 (-)	6.24 ^5^	9.25 ^5^
**14a**	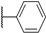	6.00 (0.92)	>1000 (0.84)	ND	ND
**14c**	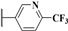	7.18 (0.97)	>1000 (0.82)	>10	>10
Pexidartinib		18.10 (0.97)	0.036 (1.0)	0.064 ± 0.04	6.54 ± 0.61

^1^ Enzymatic IC_50_ values obtained by single eight-point titrations using TR-FRET-based LANCE Ultra assay (PerkinElmer), ATP concentration: 25 μM. Goodness of fit (R^2^) for the regression is shown in parentheses. ^2^ Enzymatic Kd values obtained by single eight-point titrations using TR-FRET-based LanthaScreen Binding Assay (ThermoFisher). ^3^ Cellular assays of Ba/F3 cells engineered to be dependent on CSF1R with standard deviation. ^4^ Cellular assays of Ba/F3 cells engineered to be dependent on CSF1R but added additional IL-3. ^5^ One only data point.

**Table 2:** In vitro ADME profiling of selected compounds.

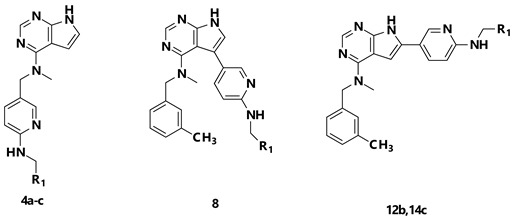							
Compound		Solubility ^3^	MLM ^1^	HLM ^2^	P_app_, A-B ^4^	P_app_, B-A ^4^	MDCK Ratio ^5^
**4a**	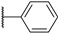	20.9	277.3	20.9	34.0	24.7	0.72
**4b**	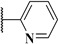	440.8	101.9	440.8	42.0	28.8	0.69
**4c**	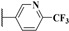	20.9	150.7	20.9	18.3	10.0	0.55
**8**	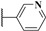	70.4	1540.3	70.4	15.9	4.4	0.27
**12b**	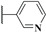	4.4	128.4	115.5	0.3	0.1	0.19
**14c**	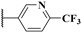	0.7	60.5	77	5.4	0.8	0.14
Pexidartinib		20.5	41.4	11.8	4.5	1.5	0.33

^1^ MLM: in vitro intrinsic clearing of compounds in mouse liver microsome (μL/min/mg). ^2^ HLM: in vitro intrinsic clearing of compounds in human liver microsome (μL/min/mg) ^3^ Kinetic solubility determined spectrophotometrically (μM) at pH 7.4 in HEPES buffer. ^4^ Apparent permeability coefficient (P_app_) measured by cell monolayer of Madin–Darby canine kidney (MDCK) cells in the A–B and B–A directions ([10^−6^ cm/s). ^5^ P_app_ ratio (B–A/A–B).

## Data Availability

Data is contained within the article or Supplementary Materials.

## Supplementary Materials

The following supporting information can be downloaded at: https://www.mdpi.com/article/10.3390/ph18060814/s1, Supporting information on inhibitor selectivity, ^1^H, ^13^C and HRMS spectra of the synthesized compounds, HPLC-trace of the final compounds and enzymatic assay curves are available as supplementary material.

## References

[1] Kumari A., Silakari O., Singh R.K. (2018). Recent advances in colony stimulating factor-1 receptor/c-FMS as an emerging target for various therapeutic implications. Biomed. Pharmacother..

[2] Xiang C.G., Li H., Tang W. (2023). Targeting CSF-1R represents an effective strategy in modulating inflammatory diseases. Pharmacol. Res..

[3] Barca C., Foray C., Hermann S., Herrlinger U., Remory I., Laoui D., Schaefers M., Grauer O.M., Zinnhardt B., Jacobs A.H. (2021). The Colony Stimulating Factor-1 Receptor (CSF-1R)-Mediated Regulation of Microglia/Macrophages as a Target for Neurological Disorders (Glioma, Stroke). Front. Immunol..

[4] Abu-Duhier F.M., Goodeve A.C., Care R.S., Gari M., Wilson G.A., Peake I.R., Reilly J.T. (2003). Mutational analysis of class III receptor tyrosine kinases (C-KIT, C-FMS, FLT3) in idiopathic myelofibrosis. Br. J. Haematol..

[5] Tarale P., Alam M.M. (2022). Colony-stimulating factor 1 receptor signaling in the central nervous system and the potential of its pharmacological inhibitors to halt the progression of neurological disorders. Inflammopharmacology.

[6] Denny W.A., Flanagan J.U. (2021). Small-molecule CSF1R kinase inhibitors; review of patents 2015-present. Expert Opin. Ther. Pat..

[7] Tap W.D., Wainberg Z.A., Anthony S.P., Ibrahim P.N., Zhang C., Healey J.H., Chmielowski B., Staddon A.P., Cohn A.L., Shapiro G.I. (2015). Structure-Guided Blockade of CSF1R Kinase in Tenosynovial Giant-Cell Tumor. N. Engl. J. Med..

[8] Smith B.D., Kaufman M.D., Wise S.C., Ahn Y.M., Caldwell T.M., Leary C.B., Lu W.P., Tan G.G., Vogeti L., Vogeti S. (2021). Vimseltinib: A Precision CSF1R Therapy for Tenosynovial Giant Cell Tumors and Diseases Promoted by Macrophages. Mol. Cancer Ther..

[9] Xu J.M., Shen L., Zhou Z.W., Li J., Bai C.M., Chi Y.H.B.L., Li Z.P., Xu N., Li E.X., Liu T.S. (2020). Surufatinib in advanced extrapancreatic neuroendocrine tumours (SANET-ep): A randomised, double-blind, placebo-controlled, phase 3 study. Lancet Oncol..

[10] Wen J.C., Wang S.Y., Guo R.X., Liu D. (2023). CSF1R inhibitors are emerging immunotherapeutic drugs for cancer treatment. Eur. J. Med. Chem..

[11] Benner B., Good L., Quiroga D., Schultz T.E., Kassem M., Carson W.E., Cherian M.A., Sardesai S., Wesolowski R. (2020). Pexidartinib, a Novel Small Molecule CSF-1R Inhibitor in Use for Tenosynovial Giant Cell Tumor: A Systematic Review of Pre-Clinical and Clinical Development. Drug Des. Dev. Ther..

[12] Wesolowski R., Sharma N., Reebel L., Rodal M.B., Peck A., West B.L., Marimuthu A., Severson P., Karlin D.A., Dowlati A. (2019). Phase Ib study of the combination of pexidartinib (PLX3397), a CSF-1R inhibitor, and paclitaxel in patients with advanced solid tumors. Ther. Adv. Med. Oncol..

[13] Rosenbaum E., Kelly C., D’Angelo S.P., Dickson M.A., Gounder M., Keohan M.L., Movva S., Condy M., Adamson T., Mcfadyen C.R. (2019). A Phase I Study of Binimetinib (MEK162) Combined with Pexidartinib (PLX3397) in Patients with Advanced Gastrointestinal Stromal Tumor. Oncologist.

[14] Xu J.M., Shen L., Bai C.M., Wang W., Li J., Yu X.J., Li Z.P., Li E.X., Yuan X.L., Chi Y.H.B.L. (2020). Surufatinib in advanced pancreatic neuroendocrine tumours (SANET-p): A randomised, double-blind, placebo-controlled, phase 3 study. Lancet Oncol..

[15] Movva S., Spira A.I., Hamilton E.P., Wang J.S., Cohn A.L., Strauss J.F., Stacchiotti S., Tucci C., Kauh J., Nanda S. (2022). Surufatinib in US patients with soft tissue sarcoma. J. Clin. Oncol..

[16] Son Y., Jeong Y.J., Shin N.R., Oh S.J., Nam K.R., Choi H.D., Choi J.Y., Lee H.J. (2020). Inhibition of Colony-Stimulating Factor 1 Receptor by PLX3397 Prevents Amyloid Beta Pathology and Rescues Dopaminergic Signaling in Aging 5xFAD Mice. Int. J. Mol. Sci..

[17] Neal M.L., Fleming S.M., Budge K.M., Boyle A.M., Kim C., Alam G., Beier E.E., Wu L.J., Richardson J.R. (2020). Pharmacological inhibition of CSF1R by GW2580 reduces microglial proliferation and is protective against neuroinflammation and dopaminergic neurodegeneration. FASEB J..

[18] Mancuso R., Fryatt G., Cleal M., Obst J., Pipi E., Monzon-Sandoval J., Ribe E., Winchester L., Webber C., Nevado A. (2019). CSF1R inhibitor JNJ-40346527 attenuates microglial proliferation and neurodegeneration in P301S mice. Brain.

[19] Spiteri A.G., Ni D., Ling Z.L., Macia L., Campbell I.L., Hofer M.J., King N.J.C. (2022). PLX5622 Reduces Disease Severity in Lethal CNS Infection by Off-Target Inhibition of Peripheral Inflammatory Monocyte Production. Front. Immunol..

[20] Albert D.H., Tapang P., Magoc T.J., Pease L.J., Reuter D.R., Wei R.-Q., Li J., Guo J., Bousquet P.F., Ghoreishi-Haack N.S. (2006). Preclinical activity of ABT-869, a multitargeted receptor tyrosine kinase inhibitor. Mol. Cancer Ther..

[21] Zhou Y., Shan S., Li Z.B., Xin L.J., Pan D.S., Yang Q.J., Liu Y.P., Yue X.P., Liu X.R., Gao J.Z. (2017). CS2164, a novel multi-target inhibitor against tumor angiogenesis, mitosis and chronic inflammation with anti-tumor potency. Cancer Sci..

[22] Yan X.Q., Sheng X.N., Tang B.X., Chi Z.H., Cui C.L., Si L., Mao L.L., Lian B., Li S.M., Zhou L. (2017). Anti-VEGFR, PDGFR, and CSF1R tyrosine kinase inhibitor CM082 (X-82) in combination with everolimus for treatment of metastatic renal cell carcinoma: A phase 1 clinical trial. Lancet Oncol..

[23] Aarhus T.I., Bjørnstad F., Wolowczyk C., Larsen K.U., Rognstad L., Leithaug T., Unger A., Habenberger P., Wolf A., Bjørkøy G. (2023). Synthesis and Development of Highly Selective Pyrrolo[2,3- d]pyrimidine CSF1R Inhibitors Targeting the Autoinhibited Form. J. Med. Chem..

[24] Aarhus T.I., Eickhoff J., Klebl B., Unger A., Boros J., Choidas A., Zischinsky M.L., Wolowczyk C., Bjørkøy G., Sundby E. (2023). A highly selective purine-based inhibitor of CSF1R potently inhibits osteoclast differentiation. Eur. J. Med. Chem..

[25] Aarhus T.I., Teksum V., Unger A., Habenberger P., Wolf A., Eickhoff J., Klebl B., Wolowczyk C., Bjørkøy G., Sundby E. (2023). Negishi Cross-Coupling in the Preparation of Benzyl Substituted Pyrrolo[2,3-d]pyrimidine Based CSF1R Inhibitors. Eur. J. Org. Chem..

[26] Bjørnstad F., Havik S., Aarhus T.I., Mahdi I., Unger A., Habenberger P., Degenhart C., Eickhoff J., Klebl B.M., Sundby E. (2024). Pyrrolopyrimidine based CSF1R inhibitors: Attempted departure from Flatland. Eur. J. Med. Chem..

[27] Bérubé G. (2016). An overview of molecular hybrids in drug discovery. Expert Opin. Drug Discov..

[28] Soltan O.M., Shoman M.E., Abdel-Aziz S.A., Narumi A., Konno H., Abdel-Aziz M. (2021). Molecular hybrids: A five-year survey on structures of multiple targeted hybrids of protein kinase inhibitors for cancer therapy. Eur. J. Med. Chem..

[29] Hu Y., Stumpfe D., Bajorath J. (2017). Recent Advances in Scaffold Hopping. J. Med. Chem..

[30] Azhar Z., Grose R.P., Raza A., Raza Z. (2023). In silico targeting of colony-stimulating factor-1 receptor: Delineating immunotherapy in cancer. Explor. Target. Anti-Tumor Ther..

[31] Schrödinger_Release_2025-1 (2025). Glide.

[32] Reiersølmoen A.C., Aarhus T.I., Eckelt S., Nørsett K.G., Sundby E., Hoff B.H. (2019). Potent and selective EGFR inhibitors based on 5-aryl-7H-pyrrolopyrimidin-4-amines. Bioorg. Chem..

[33] Karaman M.W., Herrgard S., Treiber D.K., Gallant P., Atteridge C.E., Campbell B.T., Chan K.W., Ciceri P., Davis M.I., Edeen P.T. (2008). A quantitative analysis of kinase inhibitor selectivity. Nat. Biotechnol..

