# Supplementary material for: Design and Synthesis of Pyridine-Based Pyrrolo[2,3-d]pyrimidine Analogs as CSF1R Inhibitors: Molecular Hybridization and Scaffold Hopping Approach
Source: Pharmaceuticals (Basel). 2025 May 28;18(6):814. doi: 10.3390/ph18060814 (PMC12196264; doi:10.3390/ph18060814)
Supplement: Supplementary file 1 [file pharmaceuticals-18-00814-s001.zip › pharmaceuticals-3644988-supplementary.pdf]

Supporting Information File

Design and Synthesis of Pyridine-Based Pyrrolo[2,3-*d*]pyrimidine Analogs as CSF1R Inhibitors: Molecular Hybridization and Scaffold Hopping Approach

Srinivasulu Cherukupalli [1], Carsten Degenhart [2], Peter Habenberger [2], Anke Unger [2], Jan Eickhoff [2], Bård Helge Hoff [1,*] and Eirik Sundby [3,*]

1. Department of Chemistry, Norwegian University of Science and Technology (NTNU), Høgskoleringen 5, NO-7491 Trondheim, Norway
2. Lead Discovery Center GmbH (LDC), Otto-Hahn-Strasse 15, 44227 Dortmund, Germany
3. Department of Material Science, Norwegian University of Science and Technology (NTNU), NO-7491 Trondheim, Norway

*Corresponding authors E. Sundby, E-mail: eirik.sundby@ntnu.no and B.H. Hoff: bard.h.hoff@ntnu.no

Table of Contents

Profiling of inhibitor 14c towards a panel of 50 kinases 2

1H, 13C and some HRMS spectra of synthesized compounds 4

HPLC trace key compounds 35

Single 8-point titrations curves for enzymatic CSF1R and KIT activity, including the goodness of fit (R^2) for the regression. 37

Reported ^{1}H and ^{13}C NMR data and ChemDraw predicted ^{1}H and ^{13}C NMR data of the final compounds 39

Profiling of inhibitor 14c towards a panel of 50 kinases

Table S1. Inhibition (%) by compound **14c** towards different kinases. Values are average of two measurements at 1000 nM test concentration. The ATP concentration was equal to KM.

Kinase	ATP conc.	% inhibition
CSF1R	Km app	95
ABL2	Km app	75
ABL1	Km app	71
MINK1	Km app	69
YES1	Km app	65
EGFR	Km app	57
SRC	Km app	43
Aurora B	Km app	34
FLT3	Km app	26
LYN B	Km app	26
EPHA2	Km app	23
BLK	Km app	19
MUSK	Km app	19
LYN A	Km app	18
KIT	Km app	18
FYN	Km app	16
NEK1	Km app	16
HCK	Km app	16
LTK	Km app	15
RET	Km app	14
BTK	Km app	13
CDK1	Km app	12
CHEK1	Km app	11
ALK	Km app	11
GSK3B	Km app	11
MET	Km app	10

ERBB2	Km app	9
PDGFRB	Km app	9
PAK1	Km app	9
TEK	Km app	8
ITK	Km app	8
CAMK1D	Km app	8
PLK1	Km app	7
Aurora A	Km app	7
FER	Km app	6
FLT1	Km app	5
AXL	Km app	4
DYRK1A	Km app	4
JAK2	Km app	4
FGFR1	Km app	3
CLK1	Km app	3
FLT4	Km app	2
AKT1	Km app	2
ERBB4	Km app	2
MKNK1	Km app	1
ROCK1	Km app	-1
FRAP1	Km app	-5
JAK1	Km app	-5

1H, 13C and some HRMS spectra of synthesized compounds

N-((6-chloropyridin-3-yl)methyl)-N-methyl-7-((2-(trimethylsilyl)ethoxy)methyl)-7H-pyrrolo[2,3-d]pyrimidin-4-amine (2)

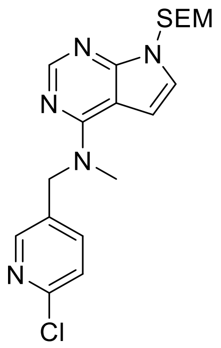

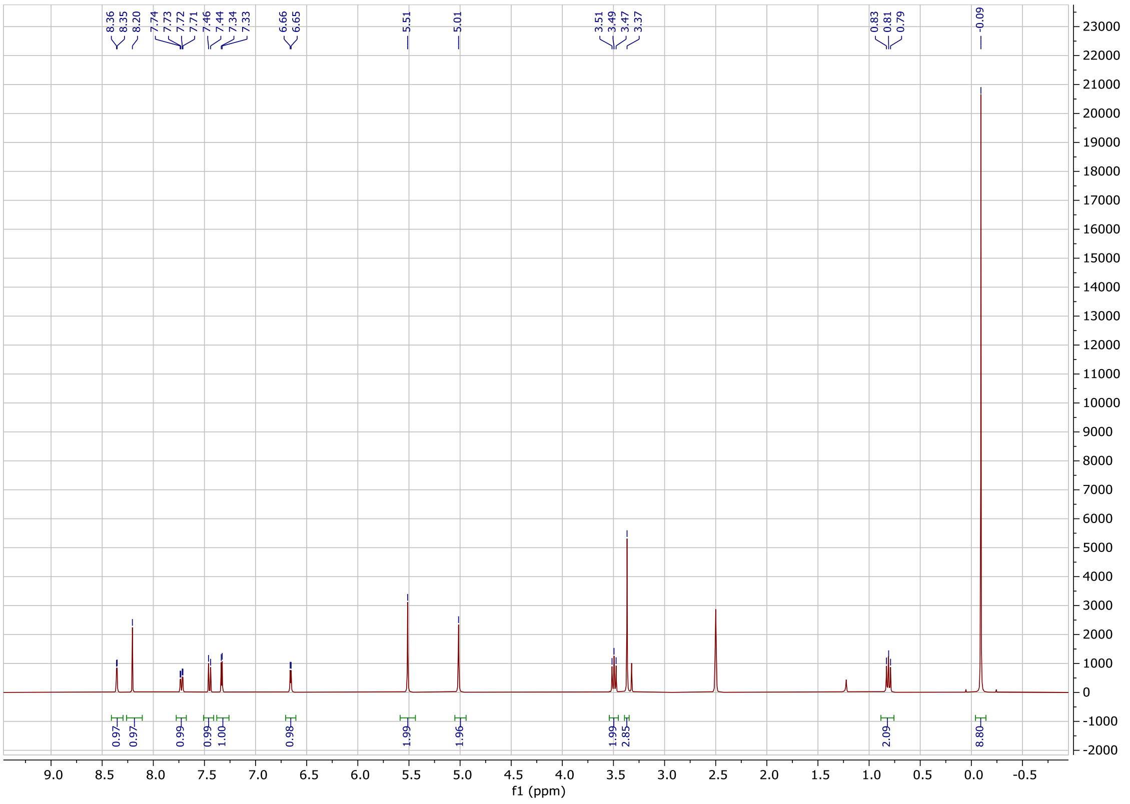

Figure S1: ^{1}H NMR (400 MHz, DMSO-d_6) spectrum of compound **2**.

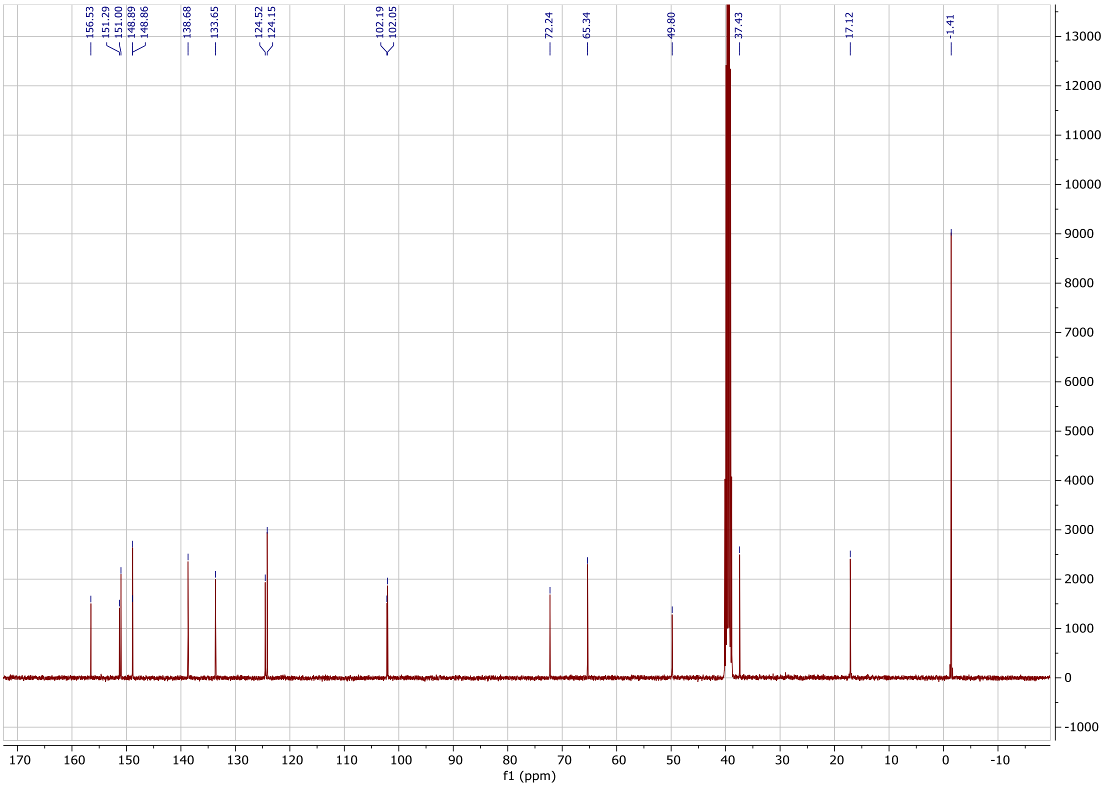

Figure S2: ^{13}C NMR (101 MHz, DMSO-d_6) spectrum of compound **2**.

Elemental Composition Report **Page 1**

Single Mass Analysis
Tolerance = 3.0 PPM / DBE: min = -1.5, max = 50.0
Element prediction: Off
Number of isotope peaks used for i-FIT = 3

Monoisotopic Mass, Even Electron Ions
4605 formula(e) evaluated with 7 results within limits (all results (up to 1000) for each mass)
Elements Used:
C: 0-60 H: 1-1000 N: 0-10 O: 0-16 Na: 0-1 Si: 0-1 Cl: 0-1
REQID2370 53 (0.507) AM2 (Ar,35000.0,0.00,0.00); Cm (48:53)
1: TOF MS ES+

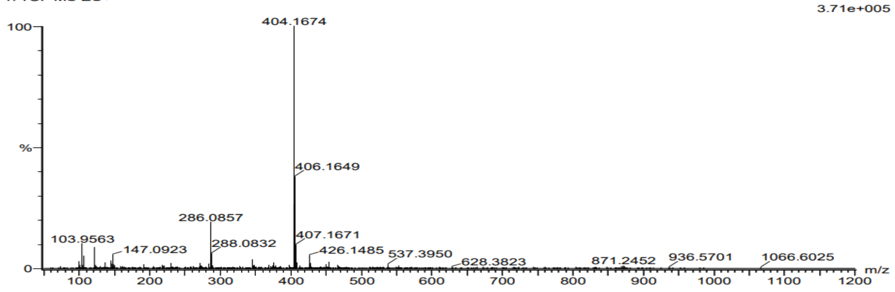

Mass	Calc. Mass	mDa	PPM	DBE	i-FIT	Norm	Conf(%)	Formula
Minimum:				-1.5				
Maximum:		3000.0	3.0	50.0				
404.1674	404.1674	0.0	0.0	1.5	943.4	18.657	0.00	C8 H26 N9 O8 Si
	404.1673	0.1	0.2	9.5	924.7	0.000	99.98	C19 H27 N5 O Si Cl
	404.1677	-0.3	-0.7	2.5	933.4	8.655	0.02	C14 H28 N5 O5 Na Cl
	404.1669	0.5	1.2	5.5	947.3	22.541	0.00	C16 H26 N3 O9
	404.1682	-0.8	-2.0	13.5	944.9	20.200	0.00	C24 H26 N O3 Si
	404.1682	-0.8	-2.0	10.5	947.4	22.704	0.00	C17 H22 N7 O5
	404.1685	-1.1	-2.7	6.5	947.7	23.015	0.00	C19 H27 N O7 Na

Figure S3: HRMS (ES+, m/z) data of compound **2**.

N-((6-(benzylamino)pyridin-3-yl)methyl)-N-methyl-7-((2-(trimethylsilyl)ethoxy)methyl)-7H-pyrrolo[2,3-d]pyrimidin-4-amine (3a)

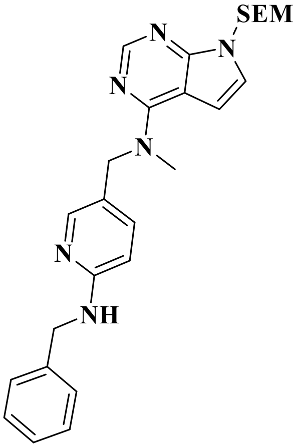

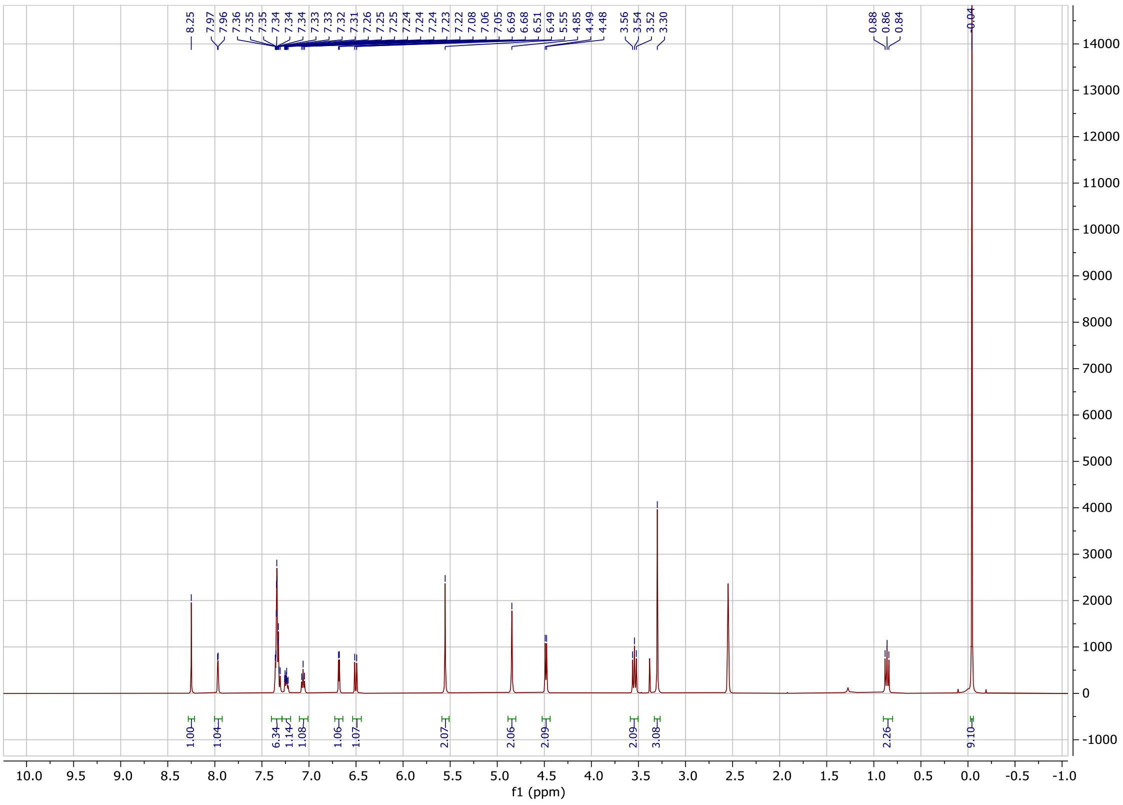

Figure S4: ^{1}H NMR (400 MHz, DMSO-d_6) spectrum of compound **3a**.

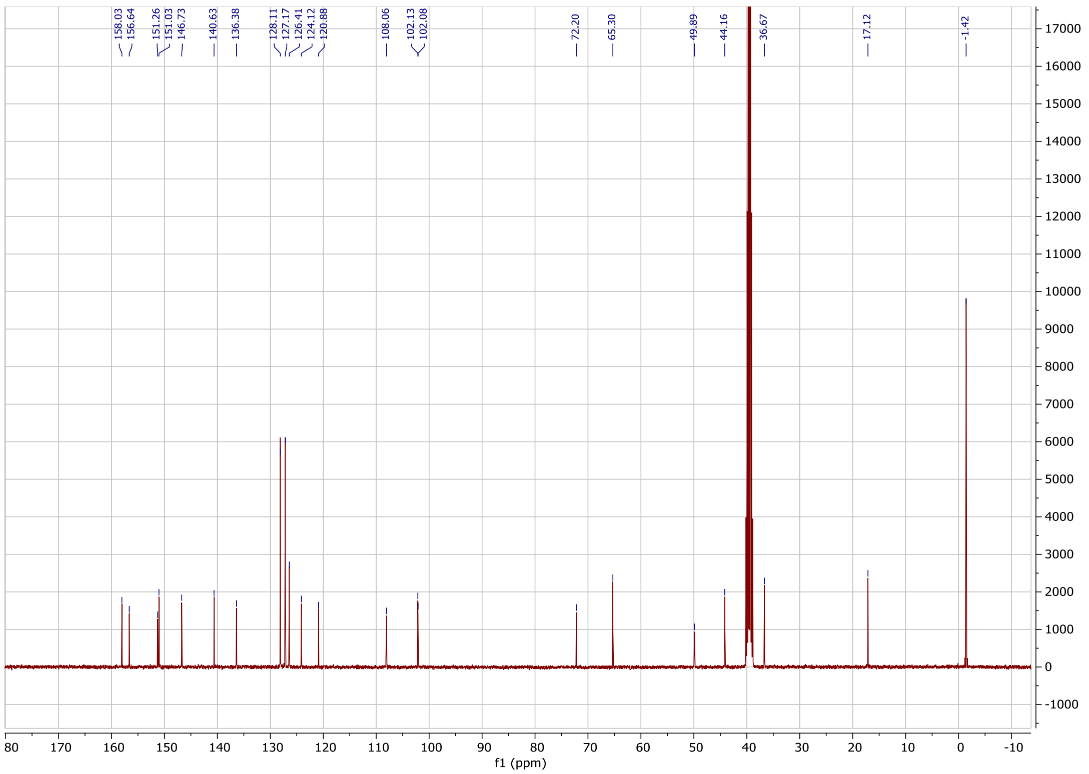

Figure S5: ^{13}C NMR (101 MHz, DMSO-d_6) spectrum of compound **3a**.

N-methyl-N-((6-(((6-(trifluoromethyl)pyridin-3-yl)methyl)amino)pyridin-3-yl)methyl)-7-((2-(trimethylsilyl)ethoxy)methyl)-7H-pyrrolo[2,3-d]pyrimidin-4-amine (3c)

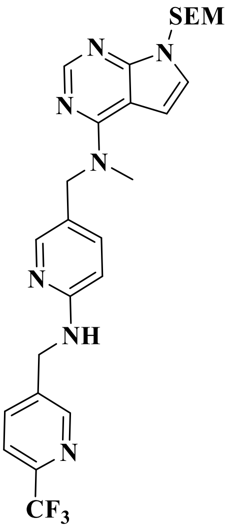

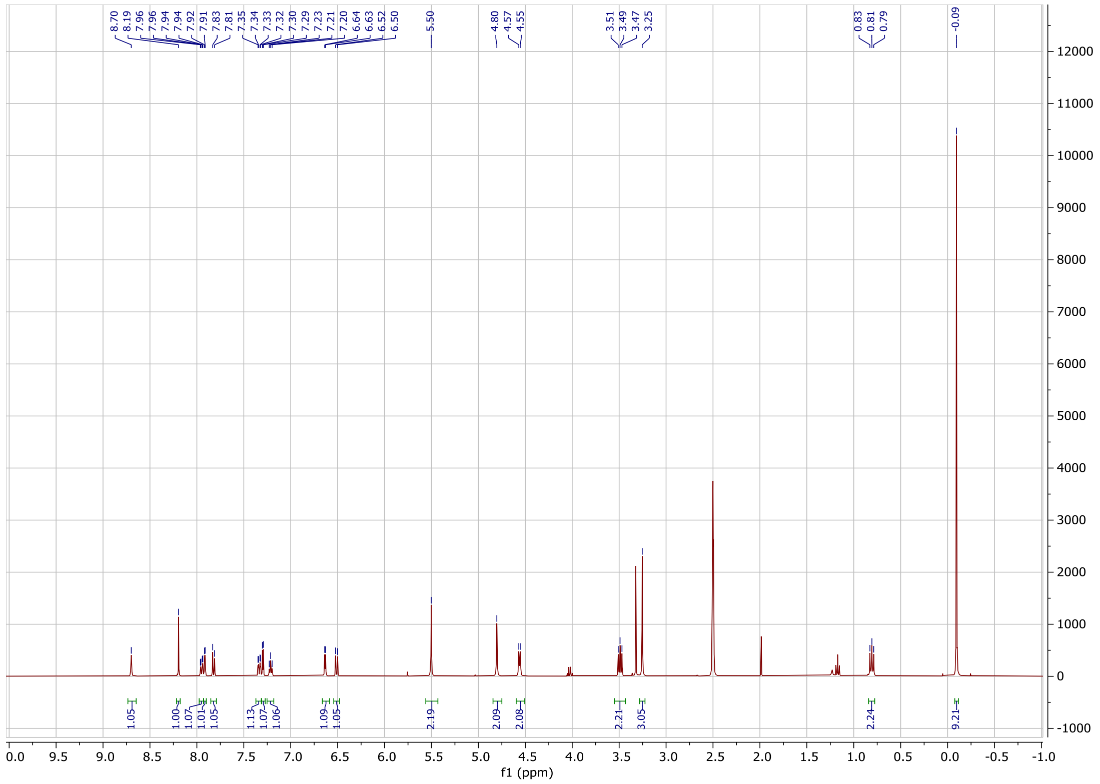

Figure S6: ^{1}H NMR (400 MHz, DMSO-d_6) spectrum of compound **3c**.

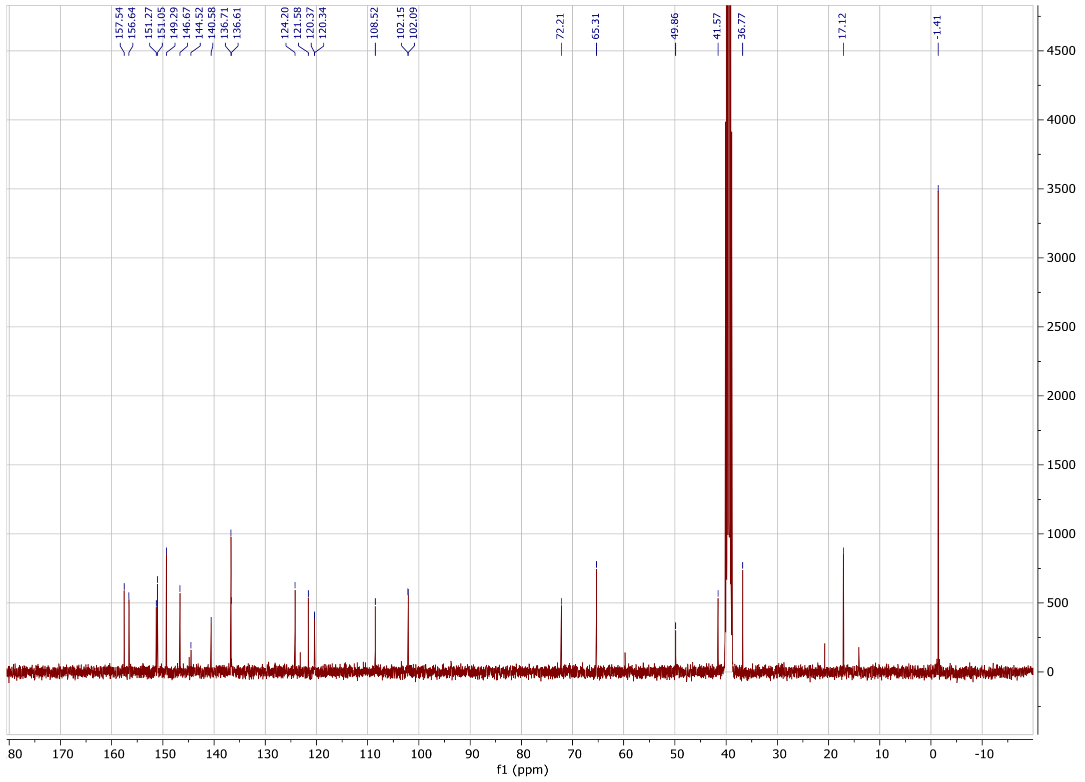

Figure S7: ^{13}C NMR (101 MHz, DMSO-d_6) spectrum of compound **3c**.

N-((6-(benzylamino)pyridin-3-yl)methyl)-N-methyl-7H-pyrrolo[2,3-d]pyrimidin-4-amine (4a)

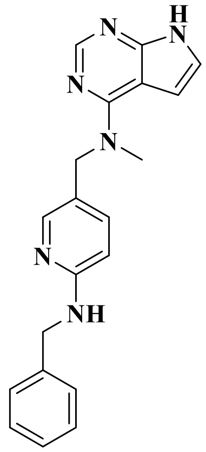

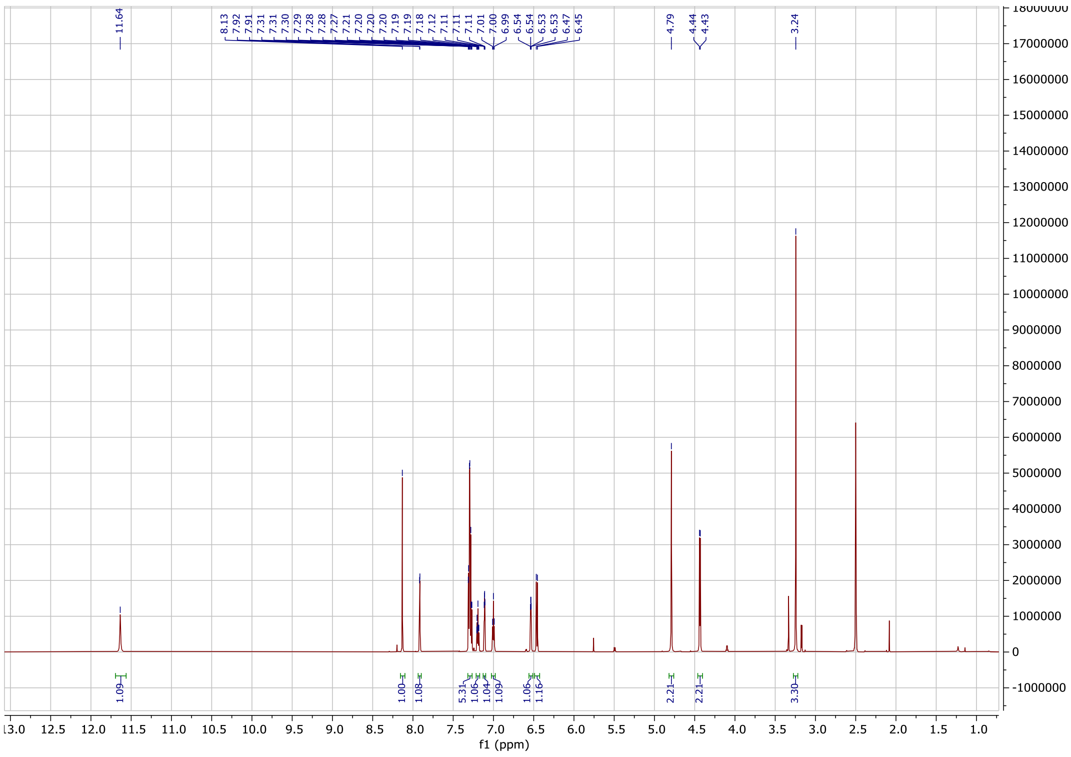

Figure S8: ^{1}H NMR (600 MHz, DMSO-d_6) spectrum of compound **4a**.

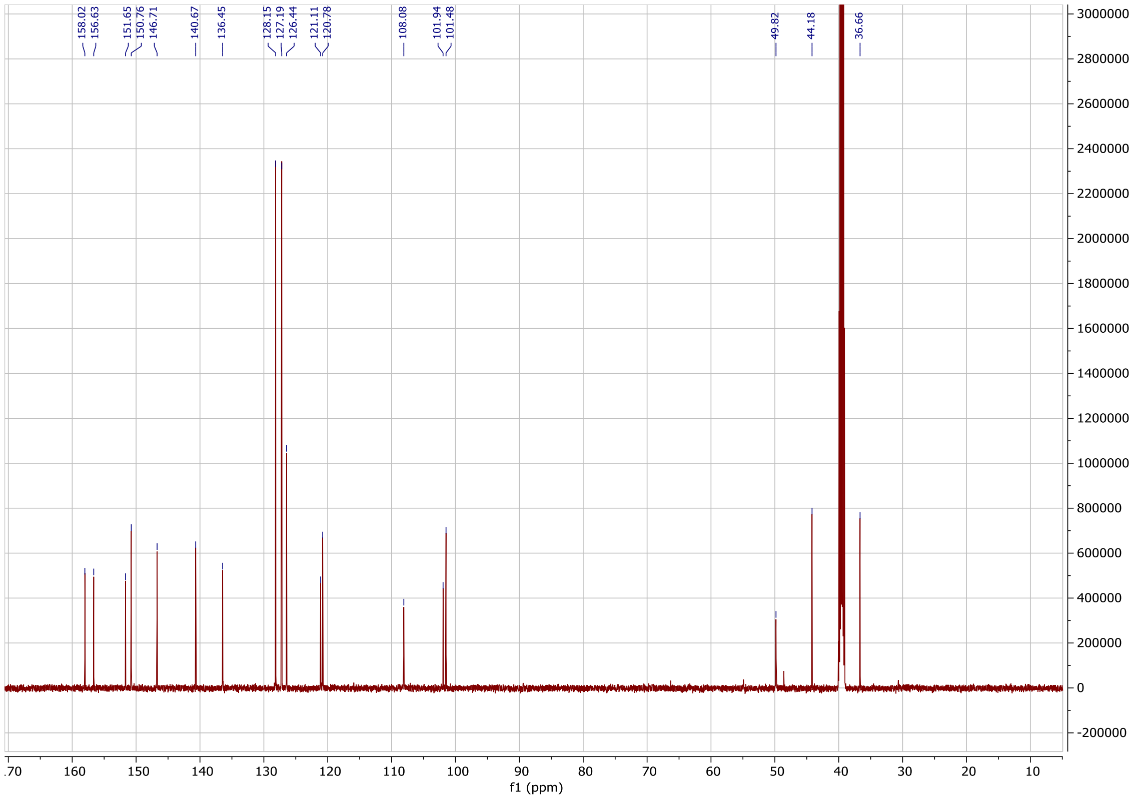

Figure S9: ^{13}C NMR (151 MHz, DMSO-d_6) spectrum of compound **4a**.

Elemental Composition Report **Page 1**

Single Mass Analysis
Tolerance = 2.0 PPM / DBE: min = -1.5, max = 50.0
Element prediction: Off
Number of isotope peaks used for i-FIT = 3

Monoisotopic Mass, Even Electron Ions
1785 formula(e) evaluated with 5 results within limits (all results (up to 1000) for each mass)
Elements Used:
C: 0-60 H: 1-1000 N: 0-10 O: 0-5 Na: 0-1 F: 0-3

REQID2421b 54 (0.516) AM2 (Ar,35000.0,0.00,0.00); Cm (45:54)
1: TOF MS ES+

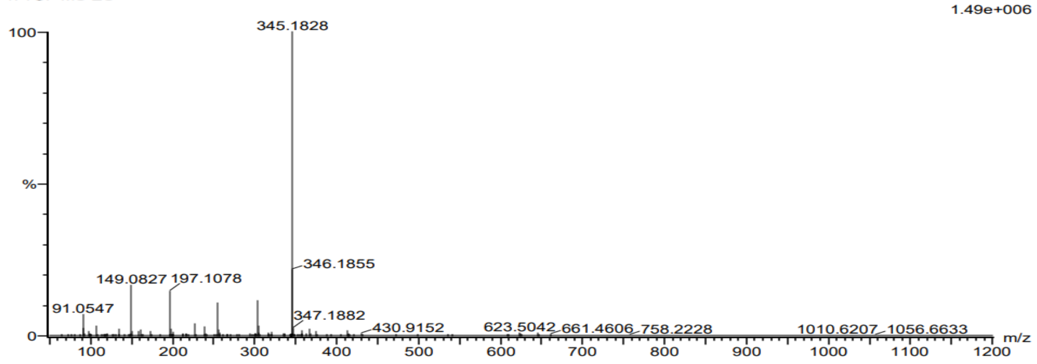

Mass	Calc. Mass	mDa	PPM	DBE	i-FIT	Norm	Conf(%)	Formula
Minimum:				-1.5				
Maximum:		3000.0	2.0	50.0				
345.1828	345.1828	0.0	0.0	13.5	1057.7	0.241	78.58	C20 H21 N6
	345.1827	0.1	0.3	2.5	1062.9	5.520	0.40	C12 H24 N6 O2 Na F2
	345.1830	-0.2	-0.6	9.5	1062.7	5.319	0.49	C22 H26 O2 Na
	345.1830	-0.2	-0.6	9.5	1062.3	4.849	0.78	C22 H24 F3
	345.1826	0.2	0.6	4.5	1059.1	1.622	19.74	C16 H26 N2 O5 F

Figure S10: HRMS (ES+, m/z) data of compound **4a**.

N-methyl-N-((6-((pyridin-2-ylmethyl)amino)pyridin-3-yl)methyl)-7H-pyrrolo[2,3-d]pyrimidin-4-amine (4b)

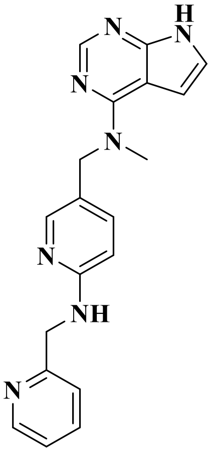

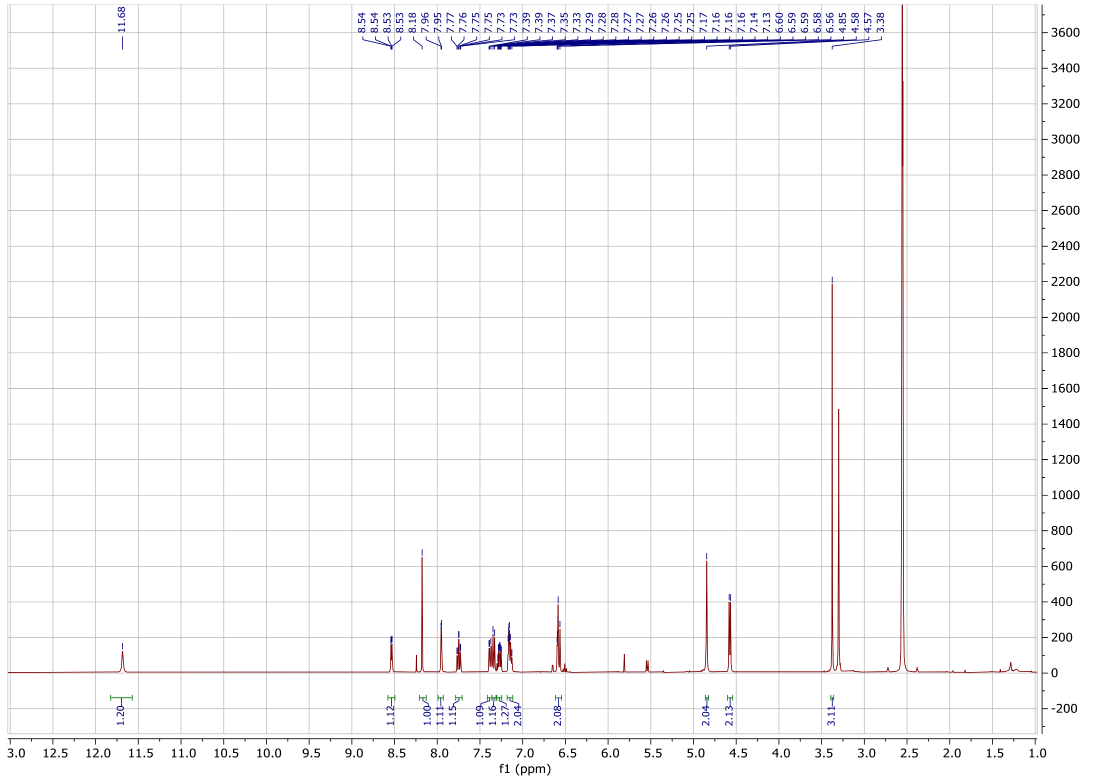

Figure S11: ^{1}H NMR (400 MHz, DMSO-d_6) spectrum of compound **4b**.

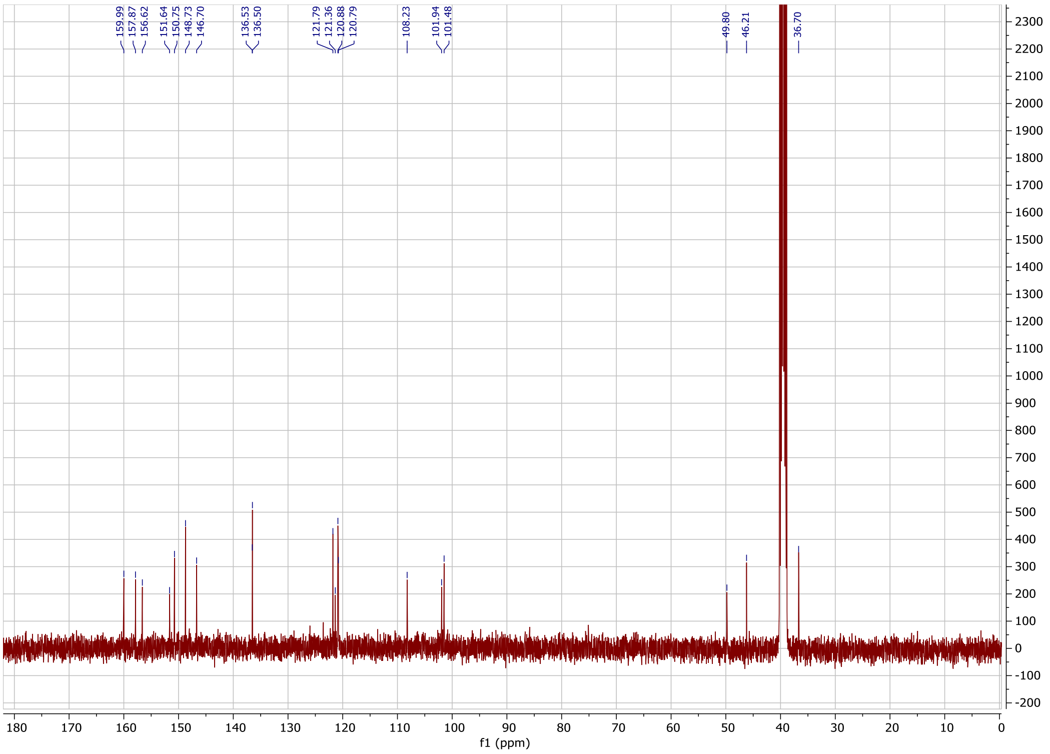

Figure S12: ^{13}C NMR (101 MHz, DMSO-d_6) spectrum of compound **4b**.

Elemental Composition Report **Page 1**

Single Mass Analysis
Tolerance = 3.0 PPM / DBE: min = 0.0, max = 50.0
Element prediction: Off
Number of isotope peaks used for i-FIT = 6

Monoisotopic Mass, Even Electron Ions
283 formula(e) evaluated with 2 results within limits (all results (up to 1000) for each mass)
Elements Used:
C: 0-59 H: 1-200 N: 0-10 O: 0-6
ReqID3186 189 (3.687) AM2 (Ar,35000.0,0.00,0.00)
1: TOF MS ASAP+

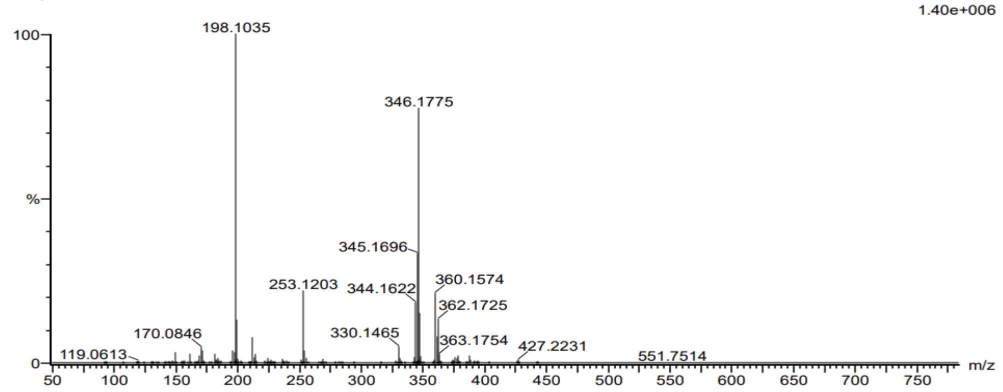

Mass	Calc. Mass	mDa	PPM	DBE	i-FIT	Norm	Conf(%)	Formula
Minimum:				0.0				
Maximum:		1000.0	3.0	50.0				
346.1775	346.1780	-0.5	-1.4	13.5	2327.3	1.791	16.68	C19 H20 N7
	346.1767	0.8	2.3	8.5	2325.7	0.182	83.32	C18 H24 N3 O4

Figure S13: HRMS (ES+, m/z) data of compound **4b**.

N-methyl-N-((6-(((6-(trifluoromethyl)pyridin-3-yl)methyl)amino)pyridin-3-yl)methyl)-7H-pyrrolo[2,3-d]pyrimidin-4-amine (4c)

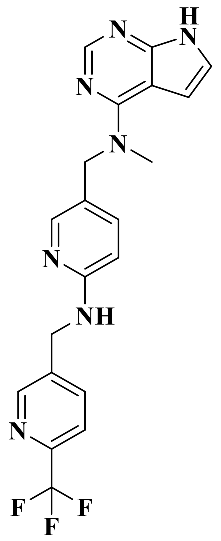

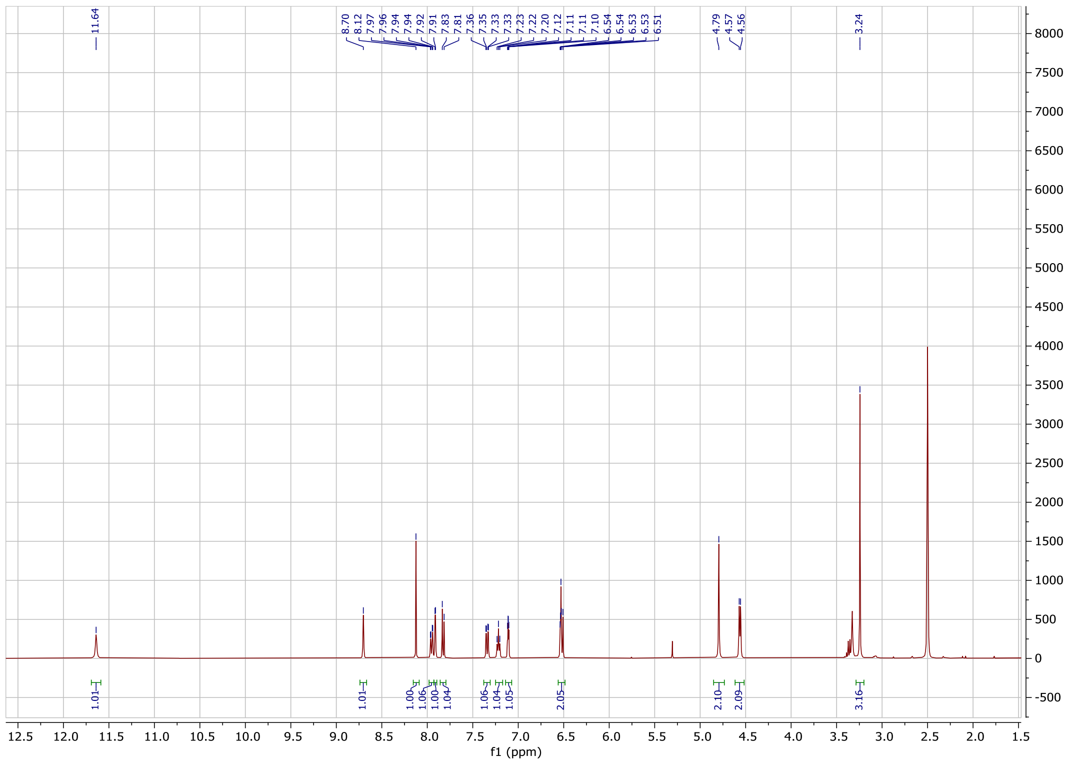

Figure S14: ^{1}H NMR (400 MHz, DMSO-d_6) spectrum of compound **4c**.

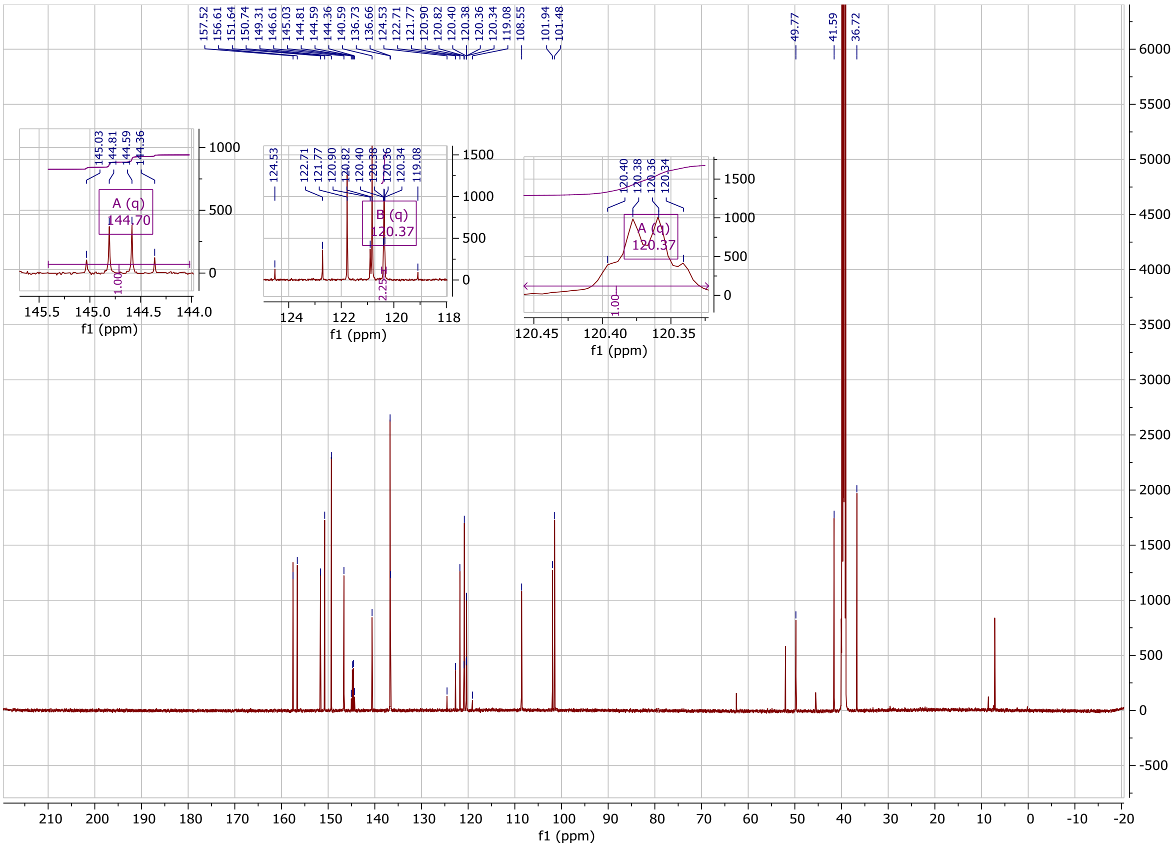

Figure S15: ^{13}C NMR (101 MHz, DMSO-d_6) spectrum of compound **4c**.

Elemental Composition Report **Page 1**

Single Mass Analysis
Tolerance = 2.0 PPM / DBE: min = -1.5, max = 50.0
Element prediction: Off
Number of isotope peaks used for i-FIT = 3

Monoisotopic Mass, Even Electron Ions
2223 formula(e) evaluated with 5 results within limits (all results (up to 1000) for each mass)
Elements Used:
C: 0-60 H: 1-1000 N: 0-10 O: 0-5 Na: 0-1 F: 0-3

REQID2422b 62 (0.594) AM2 (Ar,35000.0,0.00,0.00); Cm (55:62)
1: TOF MS ES+

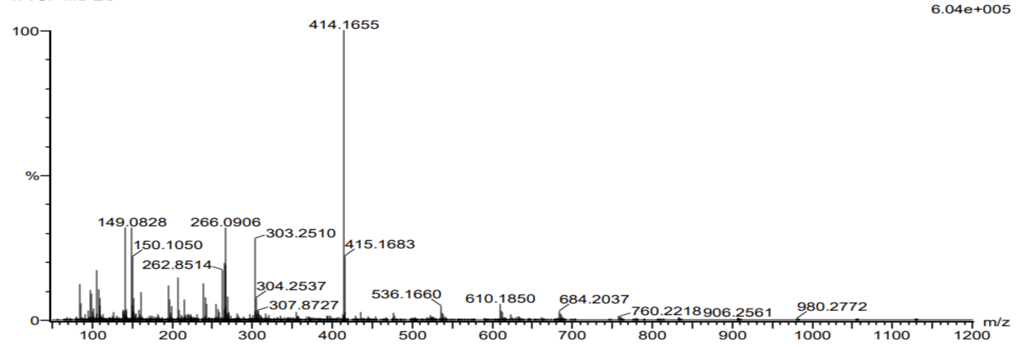

Minimum:				-1.5				
Maximum:		3000.0	2.0	50.0				
Mass	Calc. Mass	mDa	PPM	DBE	i-FIT	Norm	Conf(%)	Formula
414.1655	414.1654	0.1	0.2	13.5	908.8	0.803	44.79	C20 H19 N7 F3
	414.1657	-0.2	-0.5	9.5	908.8	0.860	42.31	C22 H24 N O2 Na F3
	414.1654	0.1	0.2	13.5	910.1	2.137	11.80	C20 H21 N7 O2 Na
	414.1650	0.5	1.2	8.5	912.6	4.629	0.98	C14 H21 N9 O5 F
	414.1658	-0.3	-0.7	20.5	914.6	6.628	0.13	C30 H21 N F

Figure S16: HRMS (ES+, m/z) data of compound **4c**.

5-Iodo-N-methyl-N-(3-methylbenzyl)-7-((2-(trimethylsilyl)ethoxy)methyl)-7H-pyrrolo[2,3-d]pyrimidin-4-amine (6)

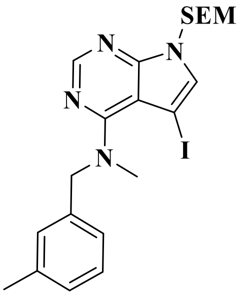

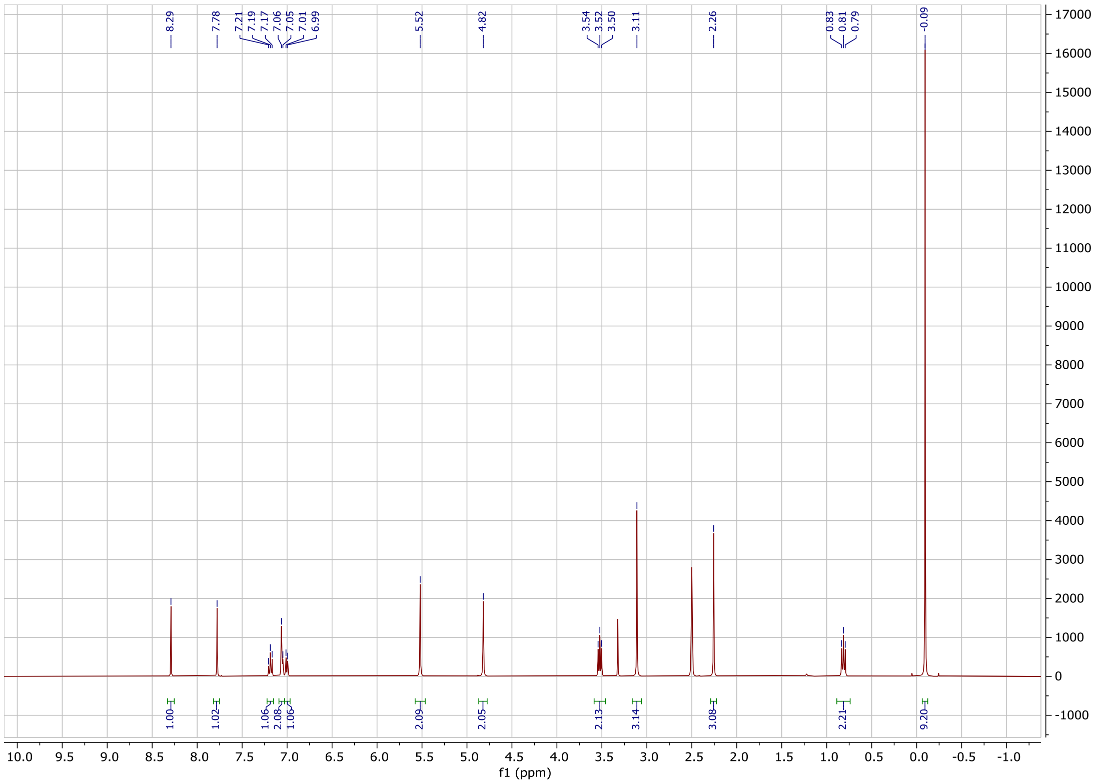

Figure S17: ^{1}H NMR (400 MHz, DMSO-d_6) spectrum of compound **6**.

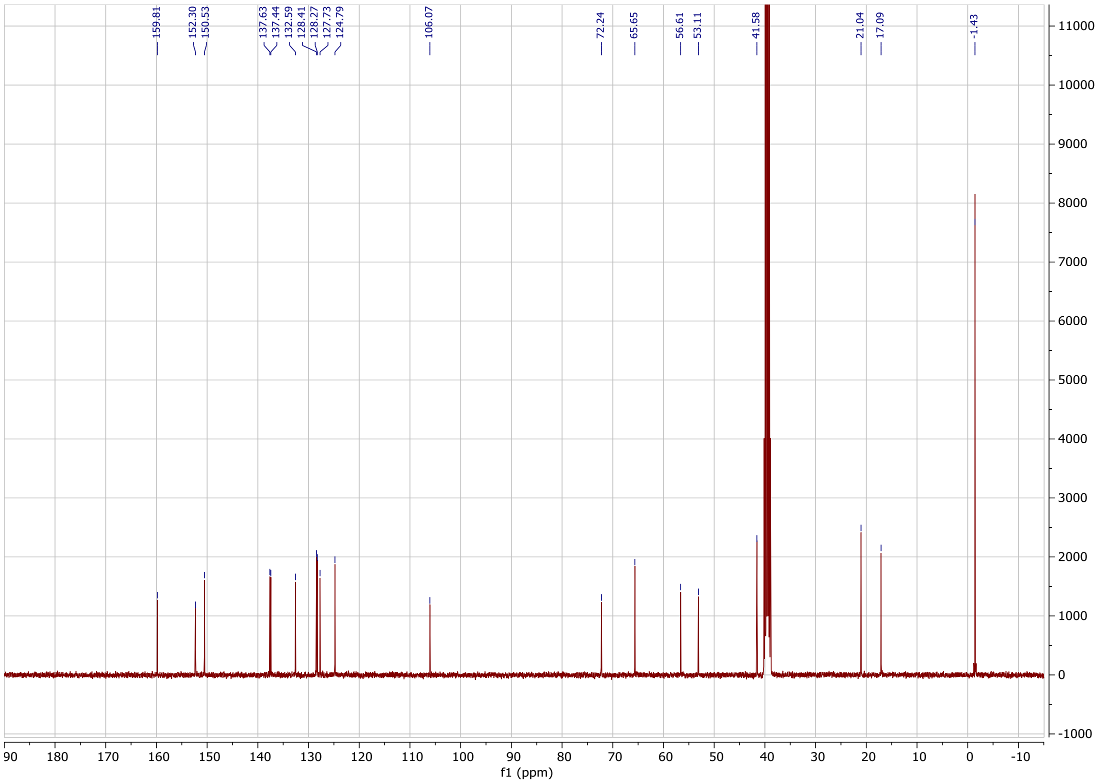

Figure S18: ^{13}C NMR (101 MHz, DMSO-d_6) spectrum of compound **6**.

5-(6-chloropyridin-3-yl)-N-methyl-N-(3-methylbenzyl)-7-((2-(trimethylsilyl)ethoxy)methyl)-7H-pyrrolo[2,3-d]pyrimidin-4-amine (7)

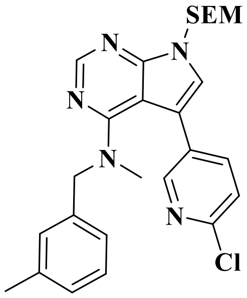

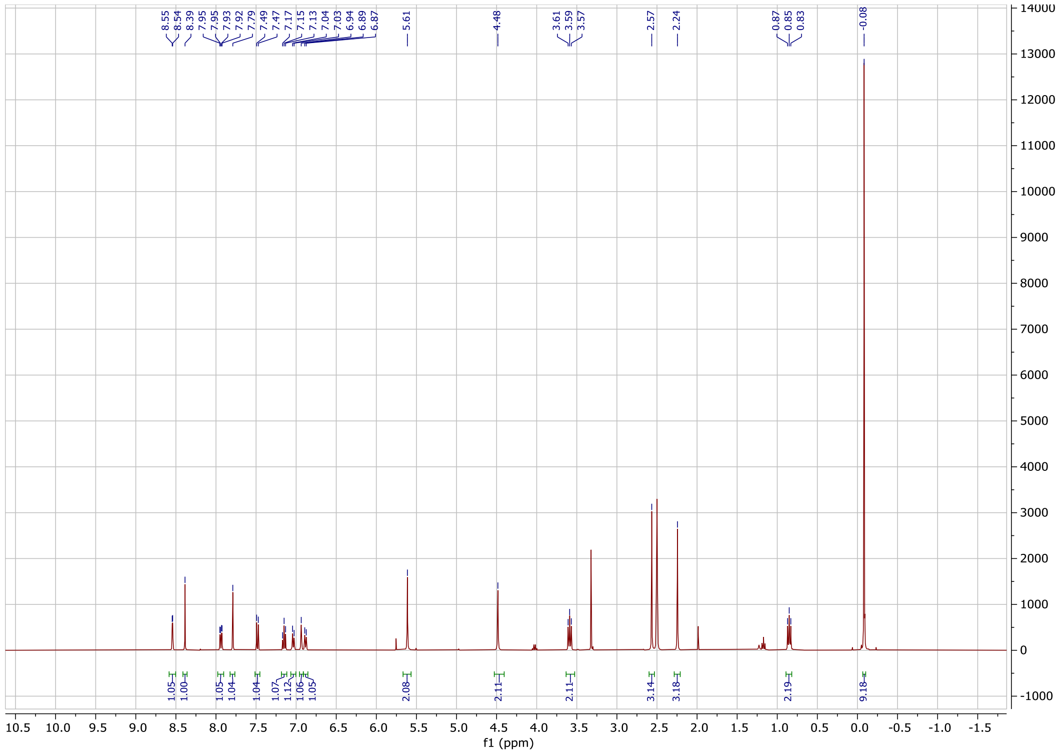

Figure S19: ^{1}H NMR (400 MHz, DMSO-d_6) spectrum of compound **7**.

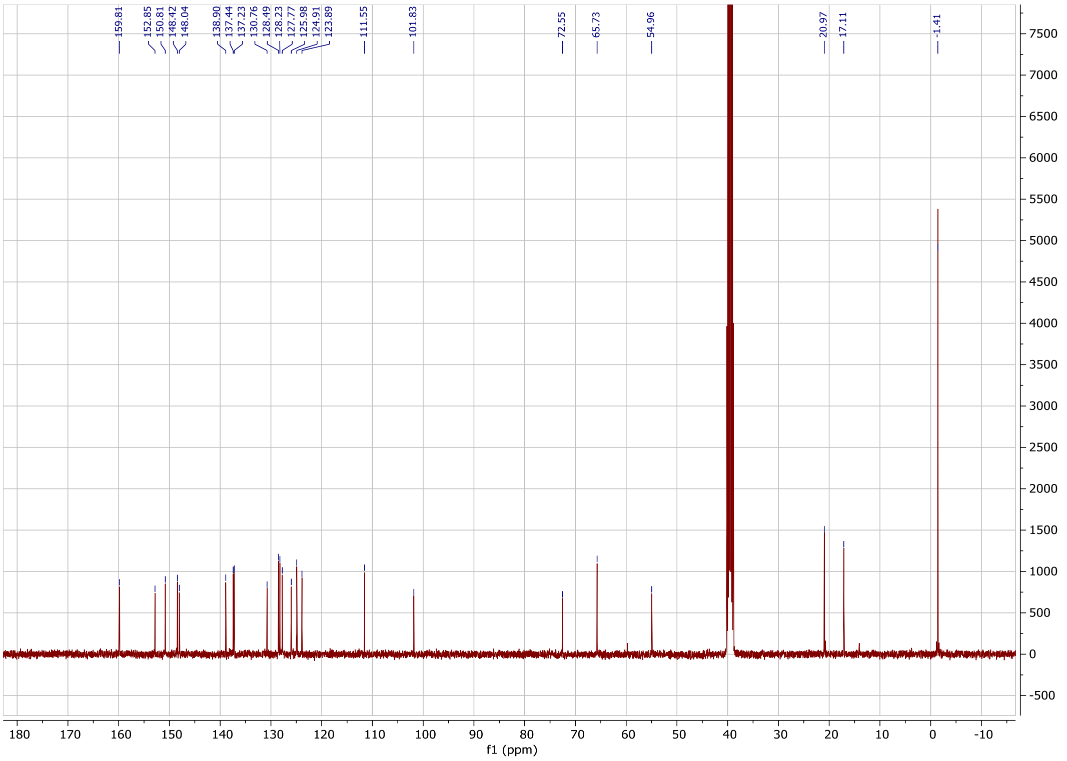

Figure S20: ^{13}C NMR (101 MHz, DMSO-d_6) spectrum of compound **7**.

N-methyl-N-(3-methylbenzyl)-5-(6-((pyridin-3-ylmethyl)amino)pyridin-3-yl)-7H-pyrrolo[2,3-d]pyrimidin-4-amine (8)

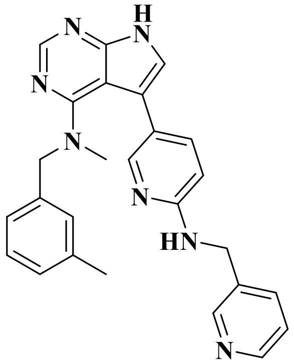

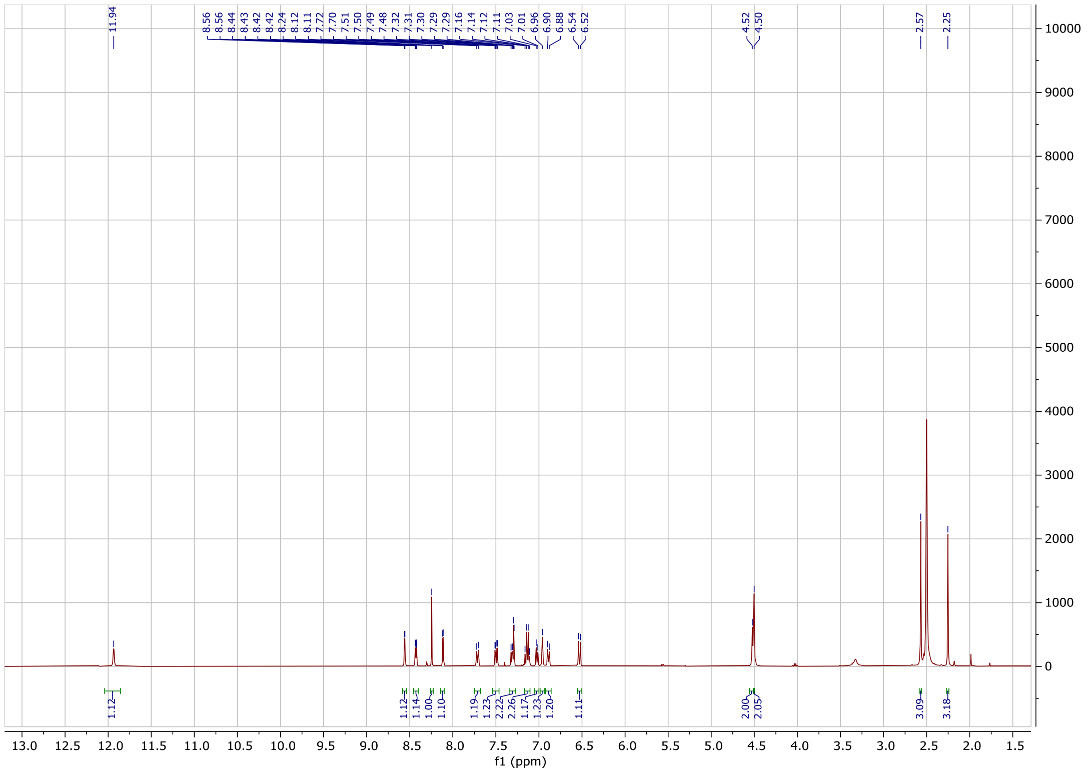

Figure S21: ^{1}H NMR (400 MHz, DMSO-d_6) spectrum of compound **8a**.

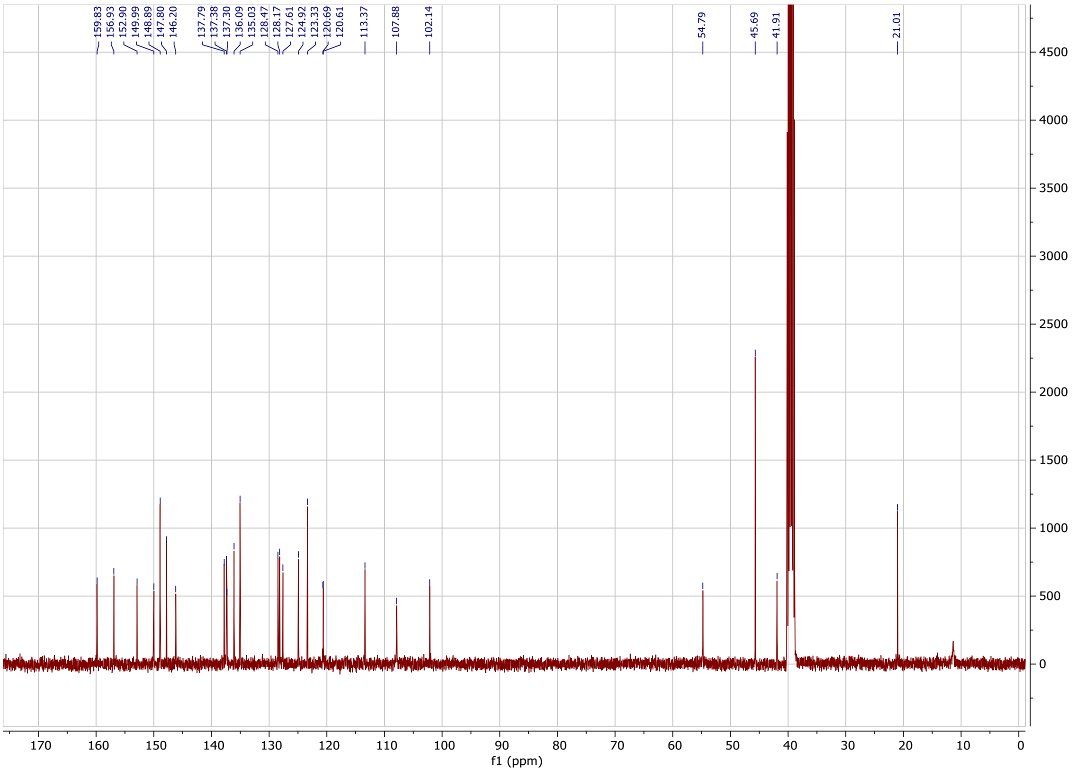

Figure S22: ^{13}C NMR (101 MHz, DMSO-d_6) spectrum of compound **8a**.

Elemental Composition Report **Page 1**

Single Mass Analysis
Tolerance = 2.0 PPM / DBE: min = -1.5, max = 50.0
Element prediction: Off
Number of isotope peaks used for i-FIT = 3

Monoisotopic Mass, Even Electron Ions
169 formula(e) evaluated with 1 results within limits (all results (up to 1000) for each mass)
Elements Used:
C: 0-100 H: 1-1000 N: 0-7 Na: 0-1 K: 0-1
REQID3115methanol 64 (0.612) AM2 (Ar,35000.0,0.00,0.00); Cm (62:70)
1: TOF MS ES+

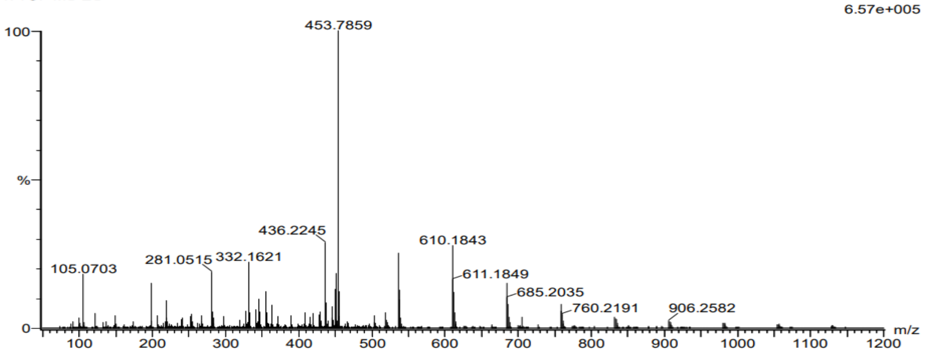

Minimum:				-1.5				
Maximum:		3000.0	2.0	50.0				
Mass	Calc. Mass	mDa	PPM	DBE	i-FIT	Norm	Conf(%)	Formula
436.2245	436.2250	-0.5	-1.1	17.5	823.4	n/a	n/a	C26 H26 N7

Figure S23: HRMS (ES+, m/z) data of compound **8a**.

6-(6-chloropyridin-3-yl)-N-methyl-N-(3-methylbenzyl)-7-((2-(trimethylsilyl) ethoxy)methyl)-7H-pyrrolo[2,3-d]pyrimidin-4-amine (10)

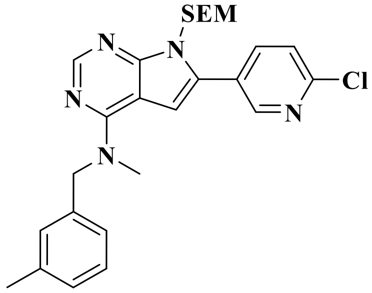

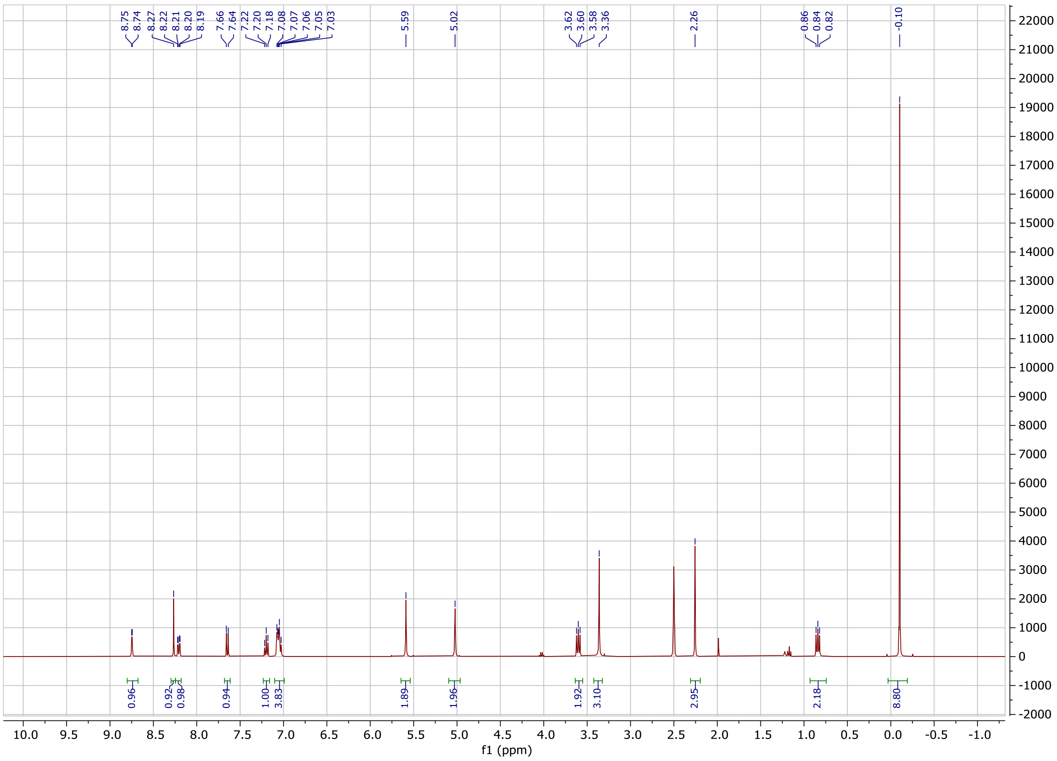

Figure S24: ^{1}H NMR (400 MHz, DMSO-d_6) spectrum of compound **10**.

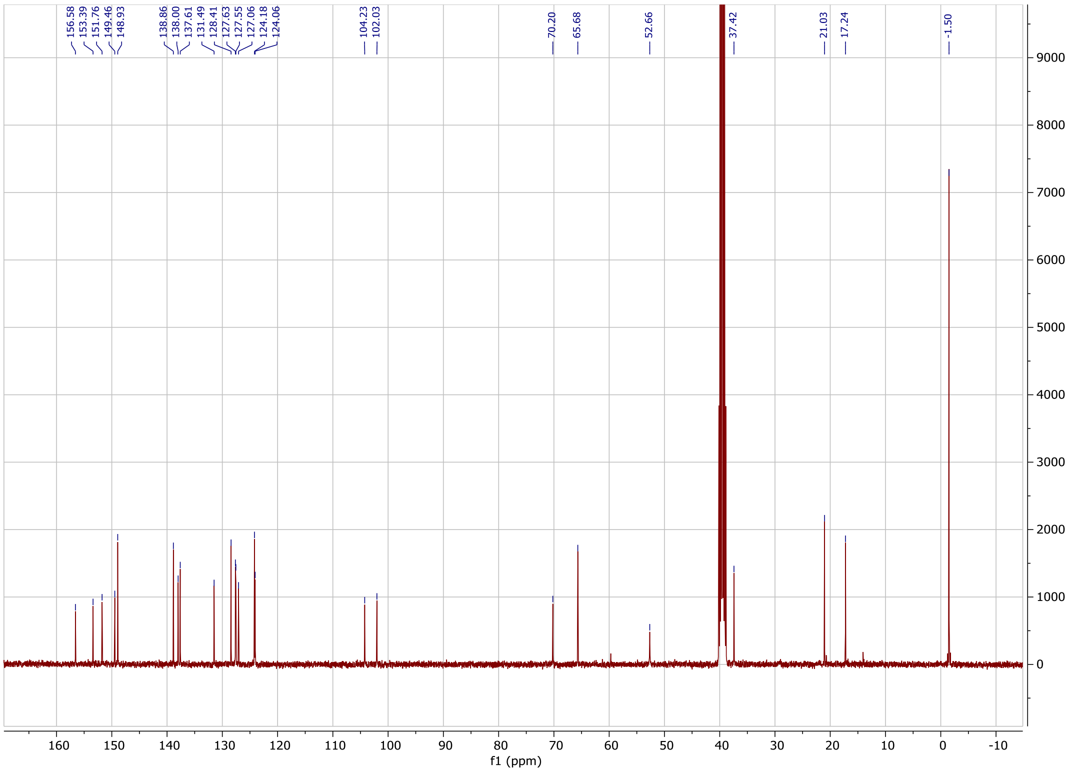

Figure 25: ^{13}C NMR (101 MHz, DMSO-d_6) spectrum of compound **10**.

N-methyl-N-(3-methylbenzyl)-6-(6-((pyridin-3-ylmethyl)amino)pyridin-3-yl)-7H-pyrrolo[2,3-d]pyrimidin-4-amine (12b)

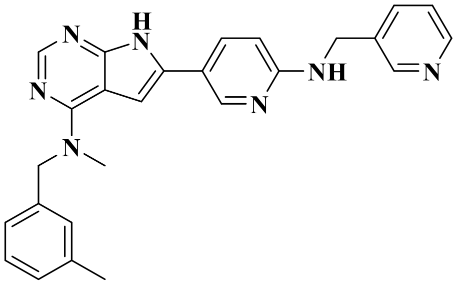

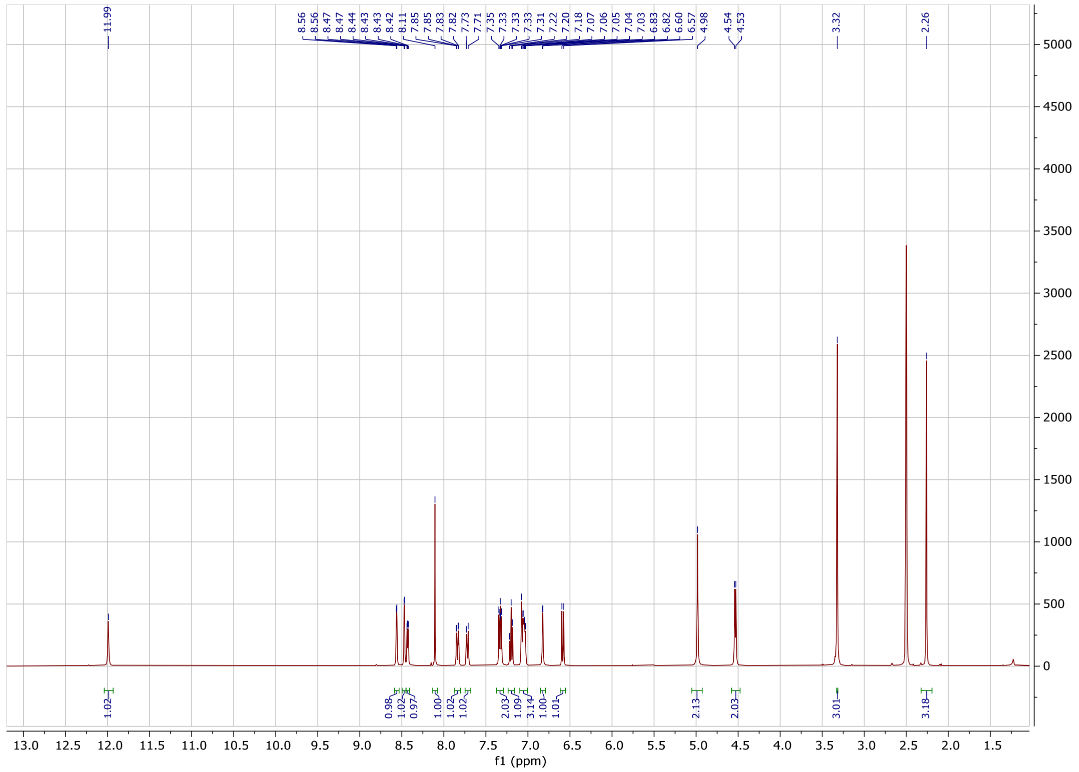

Figure S26: ^{1}H NMR (400 MHz, DMSO-d_6) spectrum of compound **12b**.

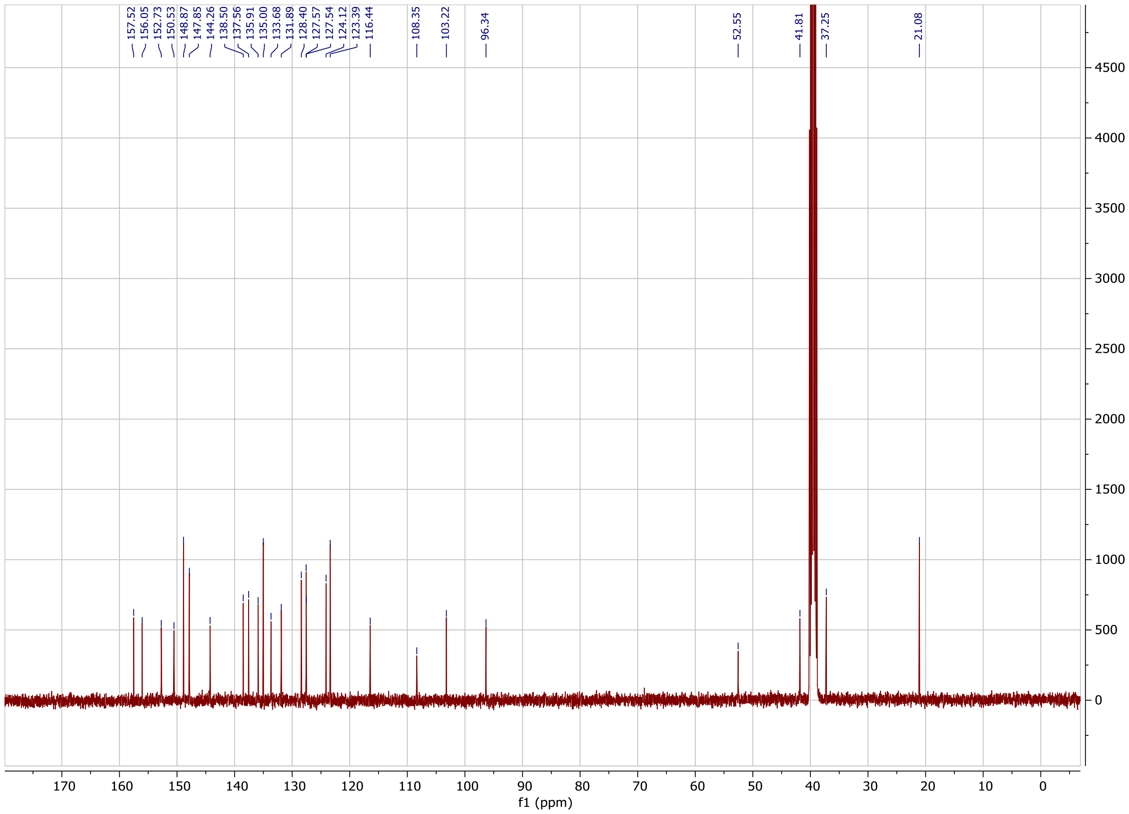

Figure S27: ^{13}C NMR (101 MHz, DMSO-d_6) spectrum of compound **12b**.

Elemental Composition Report | **Page 1**

Single Mass Analysis
Tolerance = 2.0 PPM / DBE: min = -1.5, max = 50.0
Element prediction: Off
Number of isotope peaks used for i-FIT = 3

Monoisotopic Mass, Even Electron Ions
3145 formula(e) evaluated with 3 results within limits (all results (up to 1000) for each mass)
Elements Used:
C: 1-100 H: 1-150 N: 0-8 O: 0-12 Si: 0-1 S: 0-1 I: 0-1

ReqID3693 148 (1.395) AM2 (Ar,35000.0,0.00,0.00); Cm (148:150)
1: TOF MS ES+

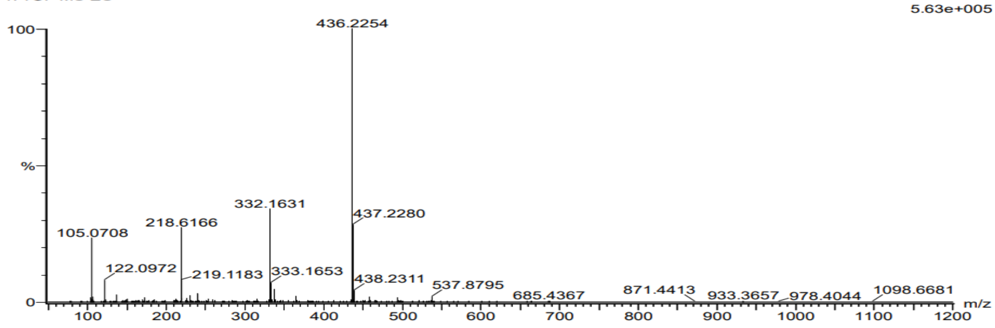

	Calc. Mass	mDa	PPM	DBE	i-FIT	Norm	Conf(%)	Formula
Minimum:				-1.5				
Maximum:		3000.0	2.0	50.0				

Mass	Calc. Mass	mDa	PPM	DBE	i-FIT	Norm	Conf(%)	Formula
436.2254	436.2250	0.4	0.9	17.5	789.3	0.001	99.95	C26 H26 N7
	436.2255	-0.1	-0.2	-0.5	796.9	7.574	0.05	C13 H34 N5 O11
	436.2261	-0.7	-1.6	-1.5	806.4	17.107	0.00	C13 H38 N5 O7 Si S

Figure S28: HRMS (ES+, m/z) data of compound **12b**.

6-(6-((2,3-dimethylbenzyl)amino)pyridin-3-yl)-N-methyl-N-(3-methylbenzyl)-7H-pyrrolo[2,3-d]pyrimidin-4-amine (12d)

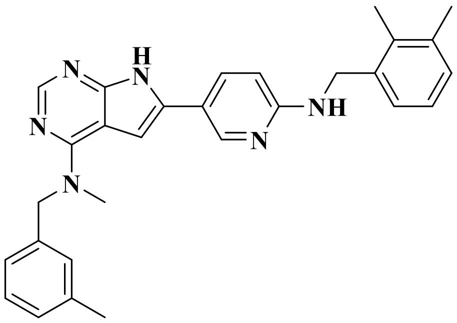

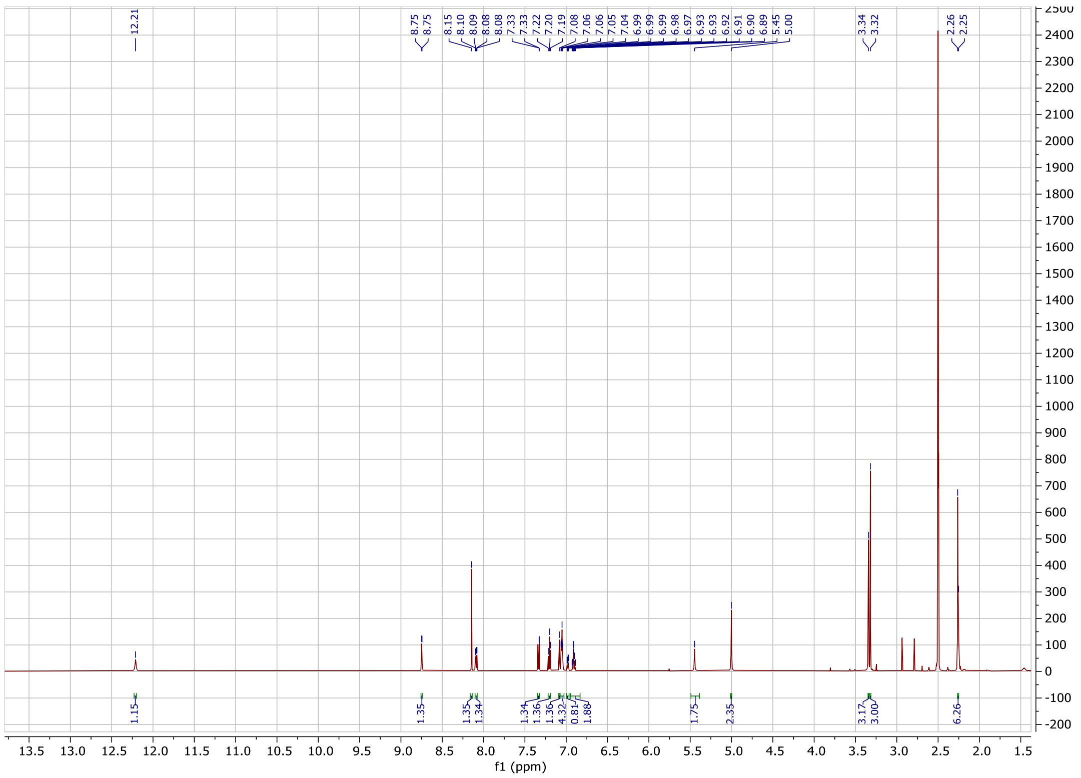

Figure S29: ^{1}H NMR (600 MHz, DMSO-d_6) spectrum of compound **12d**.

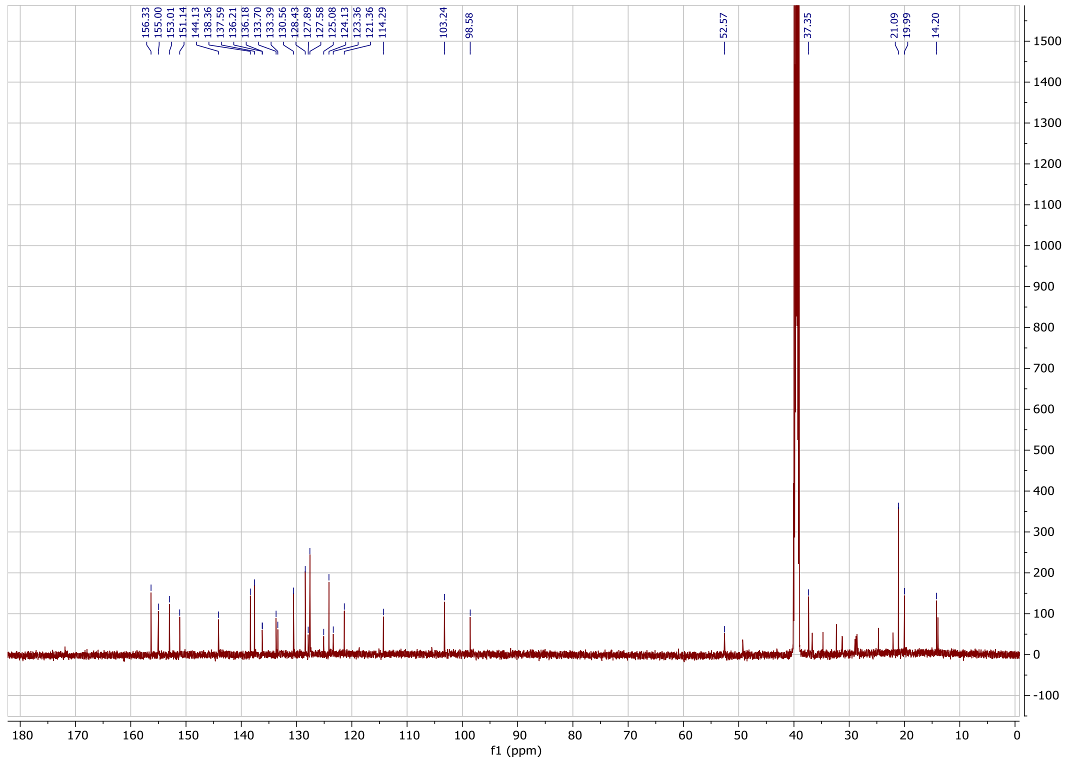

Figure S30: ^{13}C NMR (151 MHz, DMSO-d_6) spectrum of compound **12d**.

Elemental Composition Report **Page 1**

Single Mass Analysis
Tolerance = 2.0 PPM / DBE: min = -1.5, max = 50.0
Element prediction: Off
Number of isotope peaks used for i-FIT = 3

Monoisotopic Mass, Even Electron Ions
1793 formula(e) evaluated with 4 results within limits (all results (up to 1000) for each mass)
Elements Used:
C: 1-100 H: 1-150 N: 0-8 O: 0-12 Na: 0-1 I: 0-1

ReqID3695 107 (1.013) AM2 (Ar,35000.0,0.00,0.00); Cm (105:107)
1: TOF MS ES+

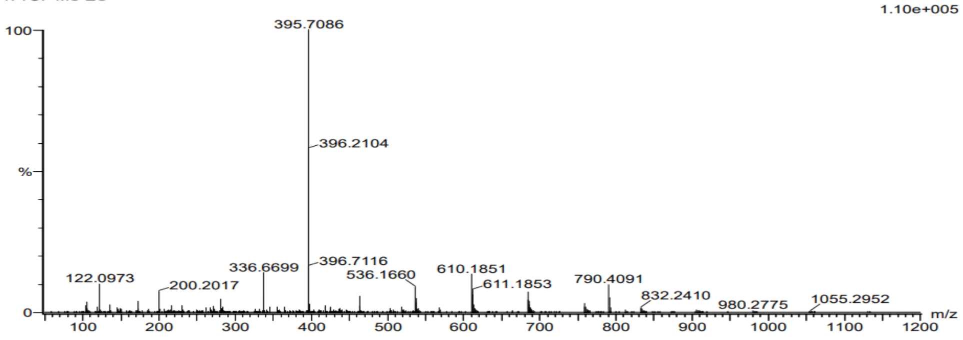

	Calc. Mass	mDa	PPM	DBE	i-FIT	Norm	Conf(%)	Formula
Minimum:				-1.5				
Maximum:		3000.0	2.0	50.0				
**Mass**								
463.2613	463.2610	0.3	0.6	17.5	363.4	0.554	57.44	C29 H31 N6
	463.2613	0.0	0.0	13.5	363.7	0.862	42.24	C31 H36 O2 Na
	463.2615	-0.2	-0.4	-0.5	369.2	6.374	0.17	C16 H39 N4 O11
	463.2605	0.8	1.7	1.5	369.4	6.545	0.14	C15 H36 N8 O7 Na

Figure S31: HRMS (ES+, m/z) data of compound **12d**.

6-(6-((4-methoxybenzyl)amino)pyridin-3-yl)-N-methyl-N-(3-methylbenzyl)-7H-pyrrolo[2,3-d]pyrimidin-4-amine (12e)

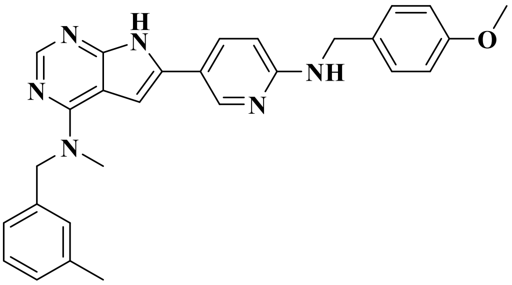

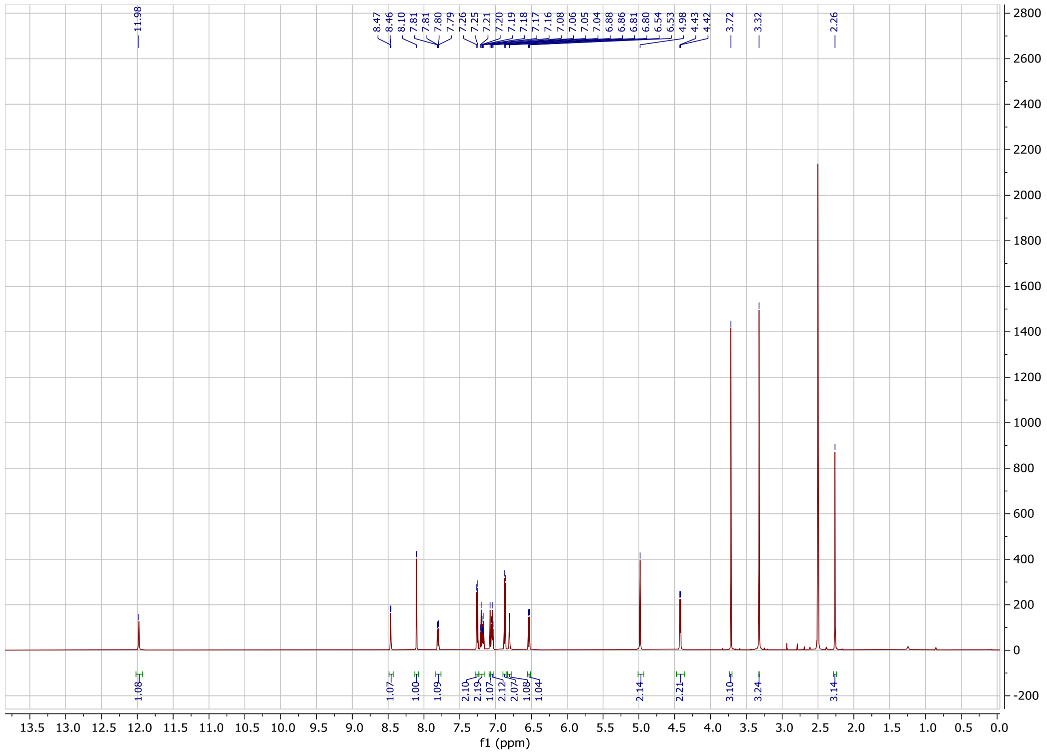

Figure S32: ^{1}H NMR (600 MHz, DMSO-d_6) spectrum of compound **12e**.

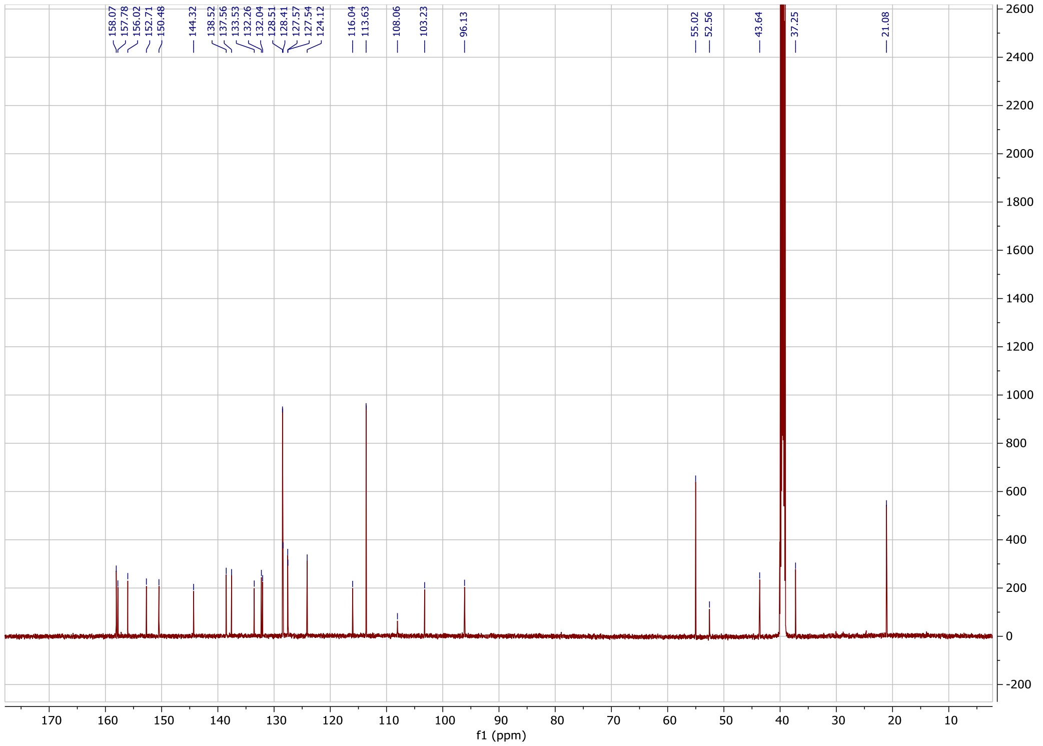

Figure S33: ^{13}C NMR (151 MHz, DMSO-d_6) spectrum of compound **12e**.

Elemental Composition Report **Page 1**

Single Mass Analysis
Tolerance = 2.0 PPM / DBE: min = -1.5, max = 50.0
Element prediction: Off
Number of isotope peaks used for i-FIT = 3

Monoisotopic Mass, Even Electron Ions
1809 formula(e) evaluated with 4 results within limits (all results (up to 1000) for each mass)
Elements Used:
C: 1-100 H: 1-150 N: 0-8 O: 0-12 Na: 0-1 I: 0-1

ReqID3696 100 (0.944) AM2 (Ar,35000.0,0.00,0.00); Cm (100:102)
1: TOF MS ES+

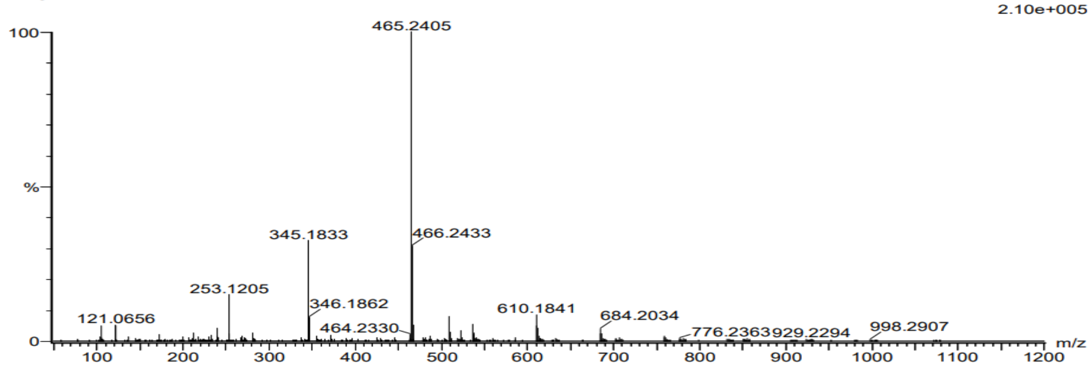

Mass	Calc. Mass	mDa	PPM	DBE	i-FIT	Norm	Conf(%)	Formula
Minimum:				-1.5				
Maximum:		3000.0	2.0	50.0				
465.2405	465.2403	0.2	0.4	17.5	631.2	0.179	83.63	C28 H29 N6 O
	465.2406	-0.1	-0.2	13.5	632.8	1.810	16.36	C30 H34 O3 Na
	465.2408	-0.3	-0.6	-0.5	640.6	9.585	0.01	C15 H37 N4 O12
	465.2397	0.8	1.7	1.5	642.6	11.547	0.00	C14 H34 N8 O8 Na

Figure S34: HRMS (ES+, m/z) data of compound **12e**.

6-(6-(benzylamino)pyridin-3-yl)-N-methyl-N-(3-methylbenzyl)-7-((2-(trimethylsilyl) ethoxy)methyl)-7H-pyrrolo[2,3-d]pyrimidin-4-amine (13a)

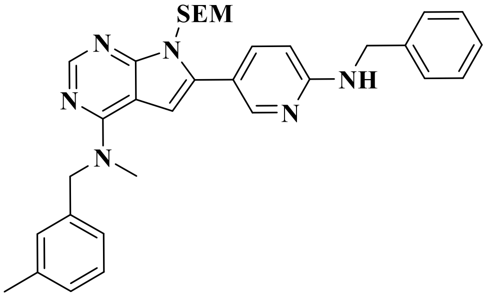

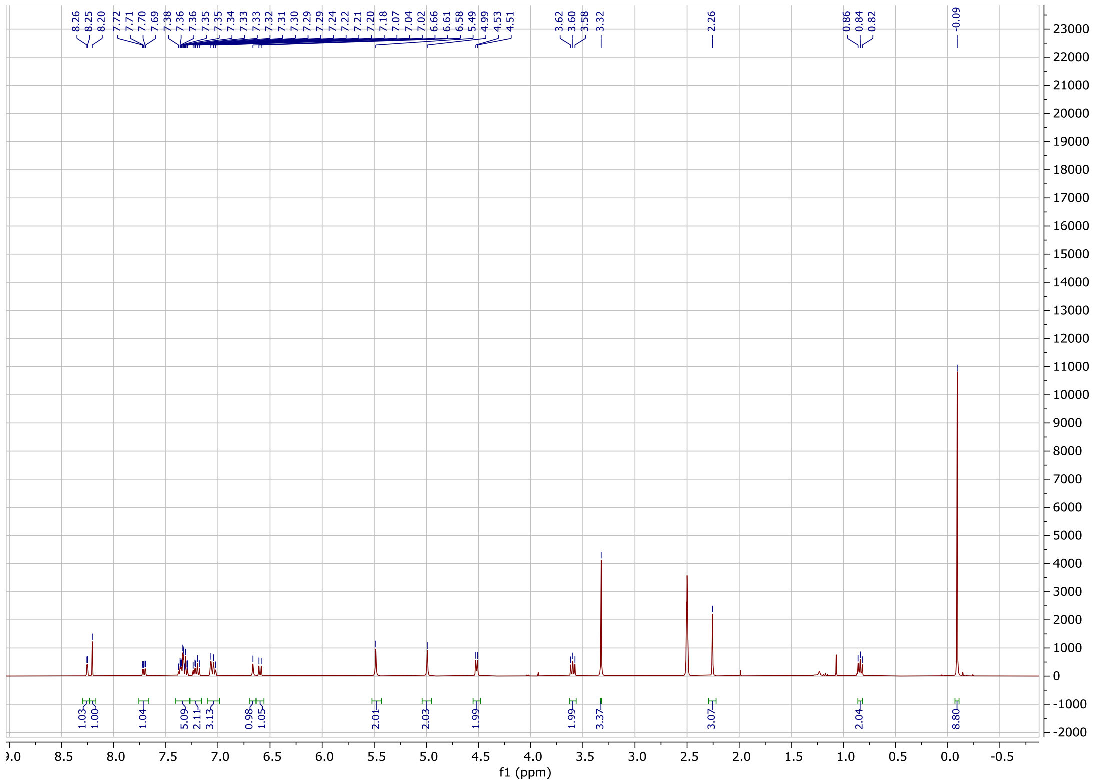

Figure S35: ^{1}H NMR (400 MHz, DMSO-d_6) spectrum of compound **13a**.

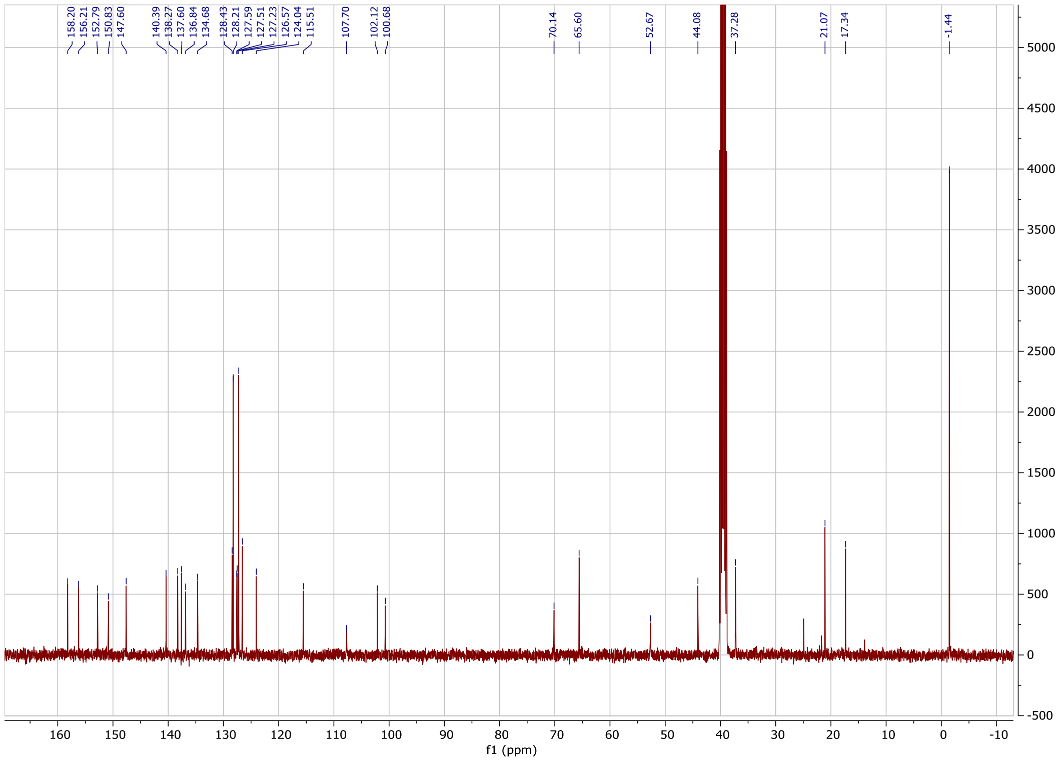

Figure S36: ^{13}C NMR (101 MHz, DMSO-d_6) spectrum of compound **13a**.

N-methyl-N-(3-methylbenzyl)-6-(6-(((6-(trifluoromethyl)pyridin-3-yl)methyl) amino)pyridin-3-yl)-7-((2-(trimethylsilyl)ethoxy)methyl)-7H-pyrrolo[2,3-d]pyrimidin-4-amine (13c)

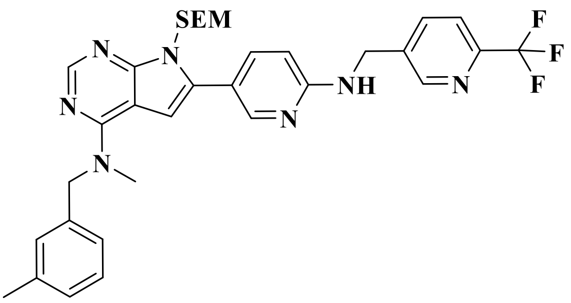

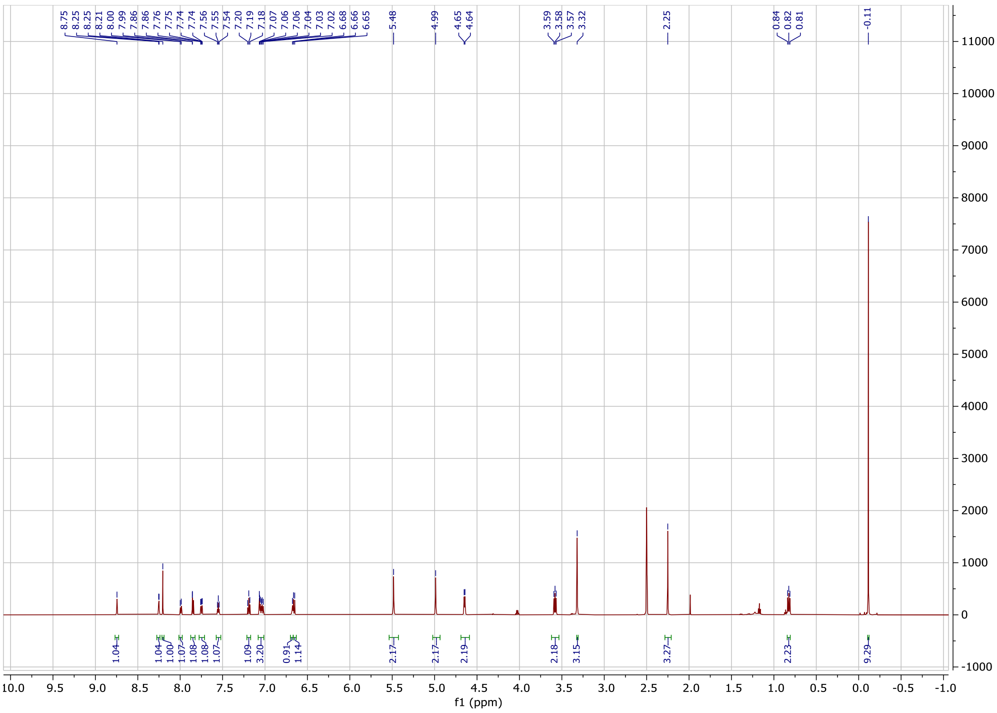

Figure S37: ^{1}H NMR (600 MHz, DMSO-d_6) spectrum of compound **13c**.

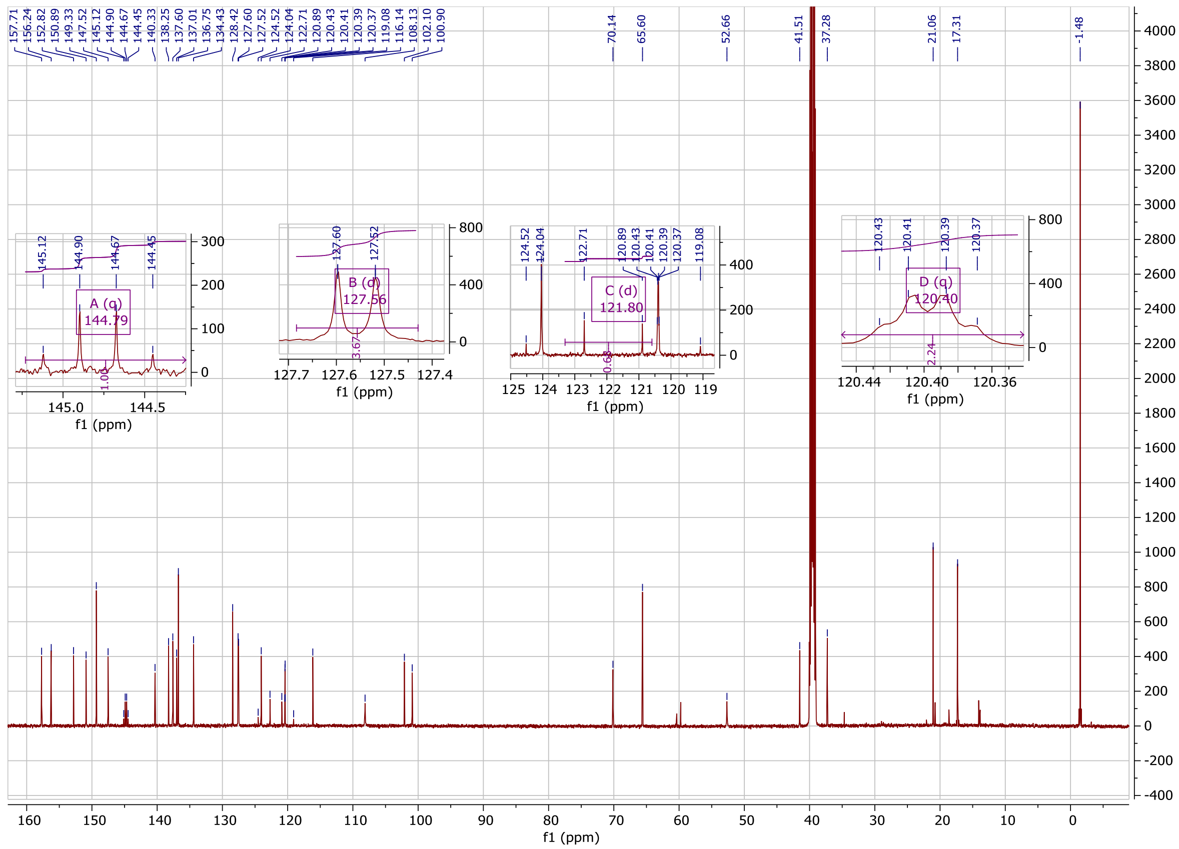

Figure S38: ^{13}C NMR (151 MHz, DMSO-d_6) spectrum of compound **13c**.

6-(6-(benzylamino)pyridin-3-yl)-N-methyl-N-(3-methylbenzyl)-7H-pyrrolo[2,3-d]pyrimidin-4-amine (14a)

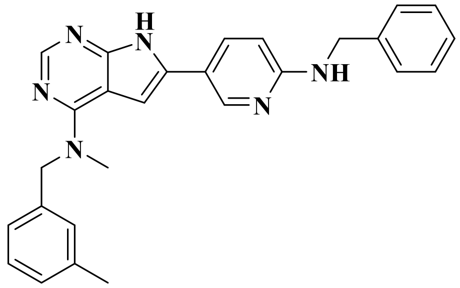

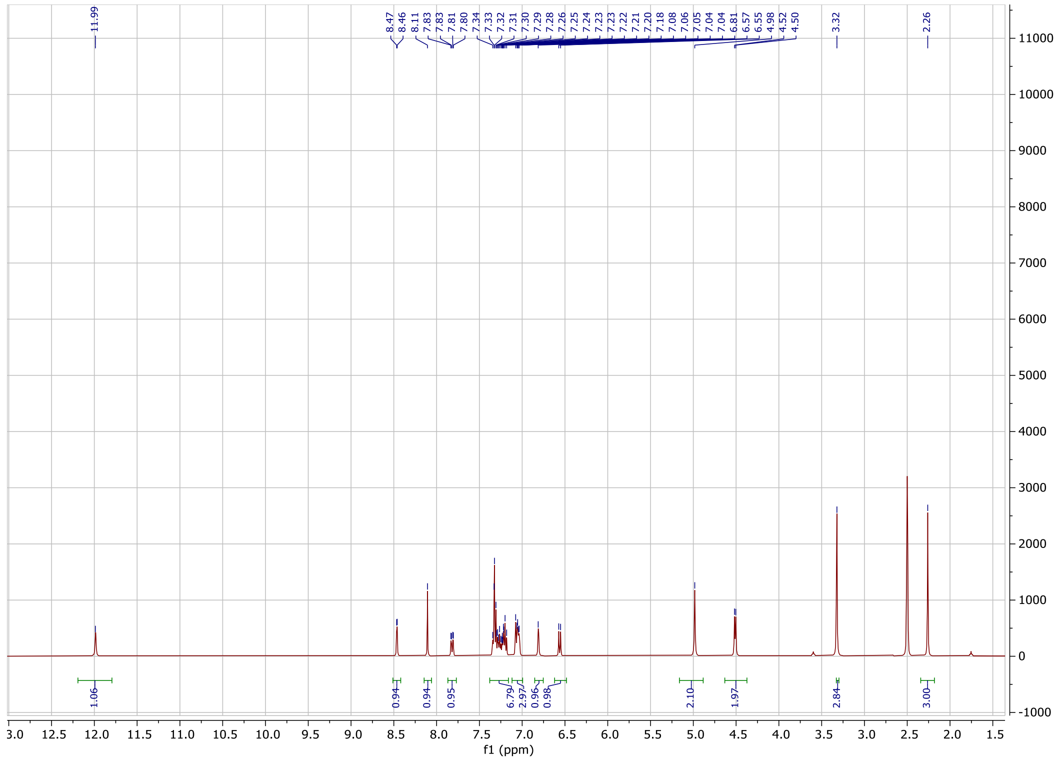

Figure S39: ^{1}H NMR (400 MHz, DMSO-d_6) spectrum of compound **14a**.

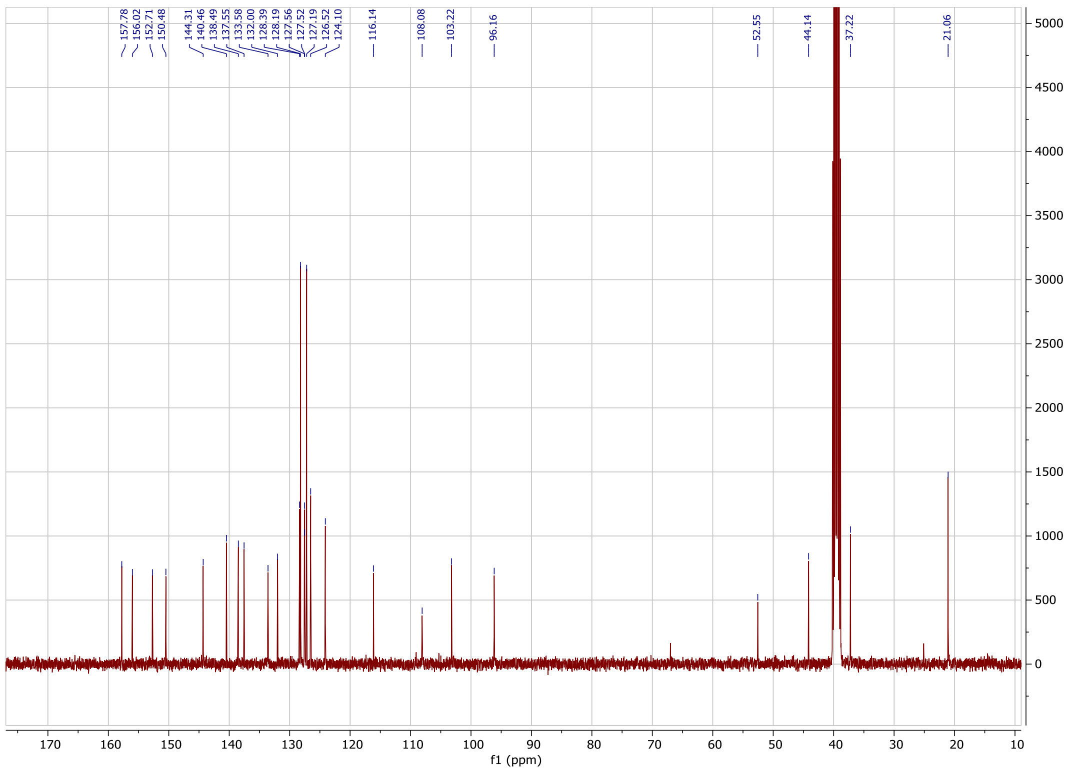

Figure S40: ^{13}C NMR (101 MHz, DMSO-d_6) spectrum of compound **14a**.

Elemental Composition Report **Page 1**

Single Mass Analysis
Tolerance = 2.0 PPM / DBE: min = -1.5, max = 50.0
Element prediction: Off
Number of isotope peaks used for i-FIT = 3

Monoisotopic Mass, Even Electron Ions
1886 formula(e) evaluated with 3 results within limits (all results (up to 1000) for each mass)
Elements Used:
C: 0-100 H: 1-1000 N: 0-7 O: 0-8 Na: 0-1 S: 0-2
REQID3116 123 (1.161) AM2 (Ar,35000.0,0.00,0.00); Cm (120:123)
1: TOF MS ES+

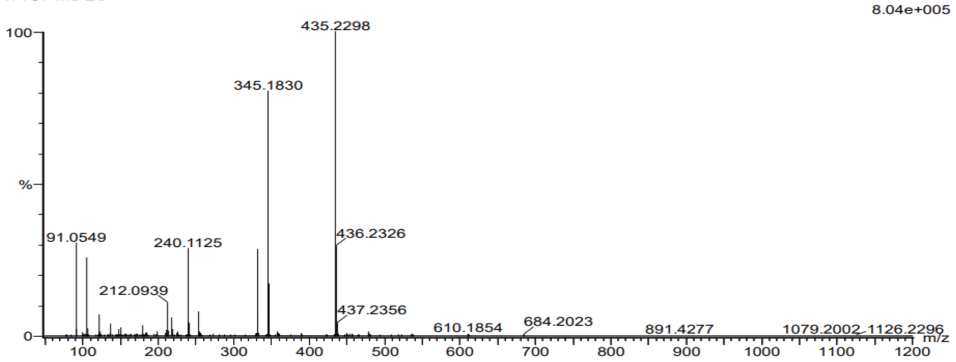

Minimum:				-1.5				
Maximum:		3000.0	2.0	50.0				
Mass	Calc. Mass	mDa	PPM	DBE	i-FIT	Norm	Conf(%)	Formula
435.2298	435.2297	0.1	0.2	17.5	902.7	0.039	96.21	C27 H27 N6
	435.2300	-0.2	-0.5	13.5	905.9	3.273	3.79	C29 H32 O2 Na
	435.2293	0.5	1.1	4.5	917.9	15.256	0.00	C21 H36 N2 O4 Na S

Figure S41: HRMS (ES+, m/z) data of compound **14a**.

N-methyl-N-(3-methylbenzyl)-6-(6-(((6-(trifluoromethyl)pyridin-3-yl)methyl) amino)pyridin-3-yl)-7H-pyrrolo[2,3-d]pyrimidin-4-amine (14c)

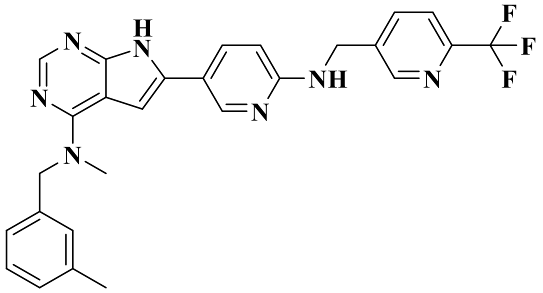

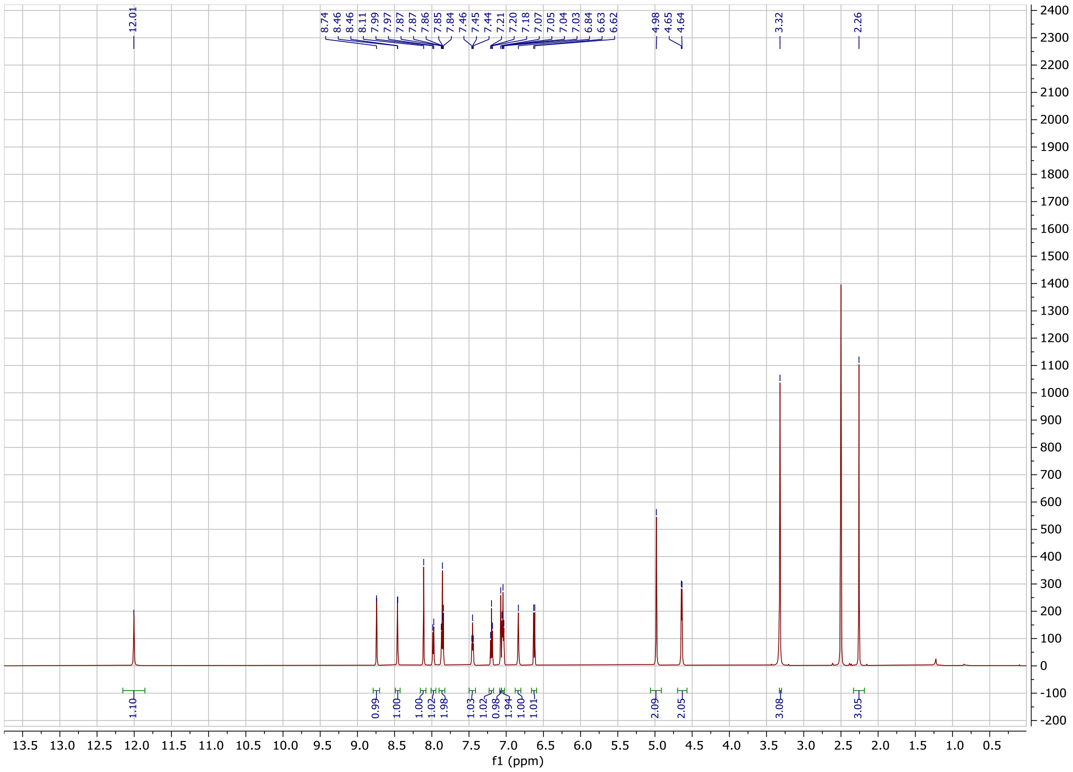

Figure S42: ^{1}H NMR (600 MHz, DMSO-d_6) spectrum of compound **14c**.

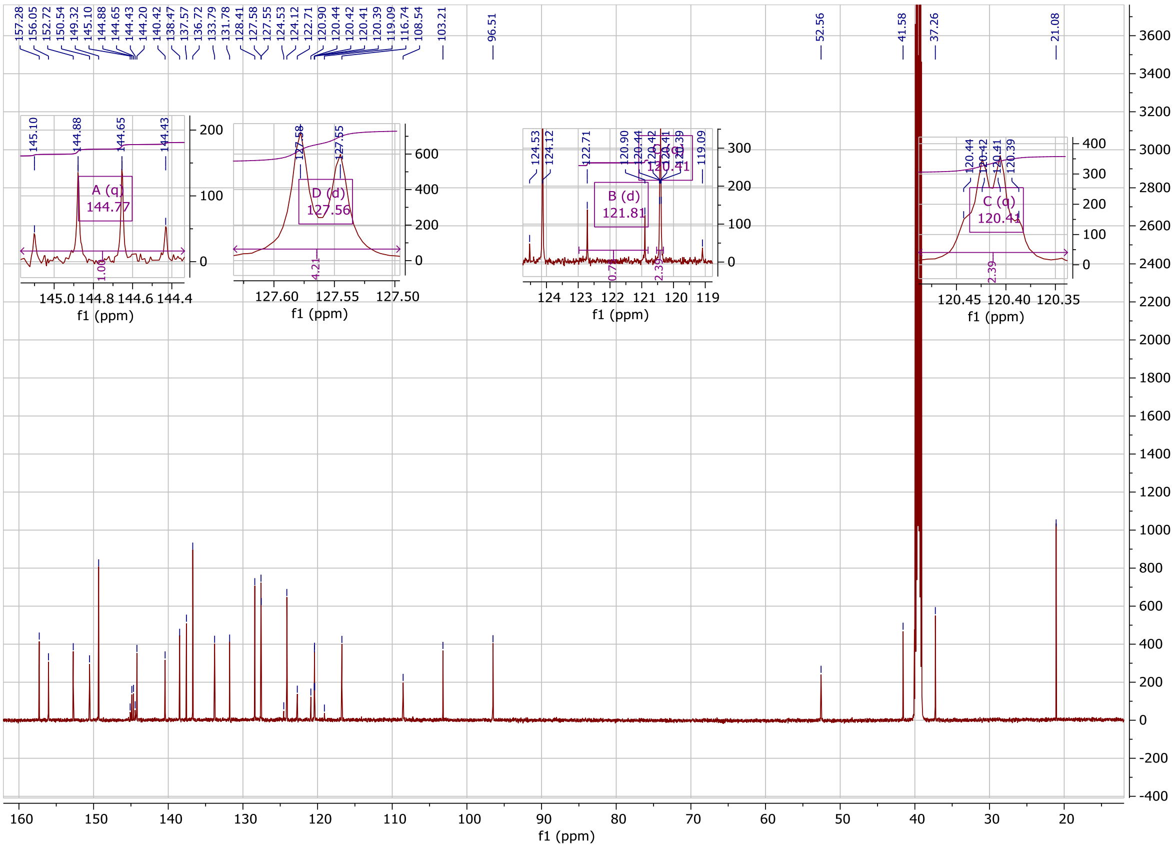

Figure S43: ^{13}C NMR (151 MHz, DMSO-d_6) spectrum of compound **14c**.

Elemental Composition Report

Single Mass Analysis
Tolerance = 2.0 PPM / DBE: min = -1.5, max = 50.0
Element prediction: Off
Number of isotope peaks used for i-FIT = 3

Monoisotopic Mass, Even Electron Ions
3806 formula(e) evaluated with 7 results within limits (all results (up to 1000) for each mass)
Elements Used:
C: 1-100 H: 1-150 N: 0-8 O: 0-12 F: 0-3 I: 0-1
ReqID3705 61 (0.586) AM2 (Ar,35000.0,0.00,0.00); Cm (59:61)
1: TOF MS ES+

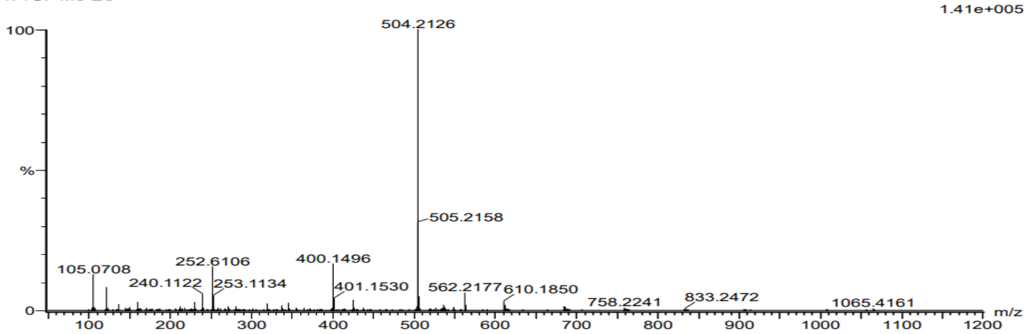

Minimum:				-1.5				
Maximum:		3000.0	2.0	50.0				

Mass	Calc. Mass	mDa	PPM	DBE	i-FIT	Norm	Conf(%)	Formula
504.2126	504.2124	0.2	0.4	17.5	523.2	0.006	99.39	C27 H25 N7 F3
	504.2127	-0.1	-0.2	8.5	528.3	5.196	0.55	C27 H39 N I
	504.2135	-0.9	-1.8	15.5	530.7	7.576	0.05	C28 H30 N3 O6
	504.2128	-0.2	-0.4	24.5	535.2	12.046	0.00	C37 H27 N F
	504.2123	0.3	0.6	1.5	535.8	12.616	0.00	C17 H37 N7 F2 I
	504.2129	-0.3	-0.6	-0.5	536.6	13.411	0.00	C14 H33 N5 O11 F3
	504.2117	0.9	1.8	3.5	537.0	13.828	0.00	C17 H32 N5 O10 F2

Figure S44: HRMS (ES+, m/z) data of compound **14c**.

HPLC trace key compounds

Compound 12b

Data File C:\Chem32\3\Data\Srini\JA_20250415_Srini_re 2025-04-15 08-08-43\1BA-0201.D
Sample Name: 12 SRI-2-05-1 re

===

Acq. Operator : SYSTEM Seq. Line : 2
Acq. Instrument : 1260 LC_DAD Location : P1-B1
Injection Date : 15.04.2025 08:40:15 Inj : 1
Inj Volume : 5.000 µl
Method : C:\Chem32\3\Data\Srini\JA_20250415_Srini_re 2025-04-15 08-08-43\KinetexC18_gradient20min.M (Sequence Method)
Last changed : 15.04.2025 08:08:43 by SYSTEM
Method Info : Standard gradient with C18 column,10:90 ACN +H2O to 100% ACN over 15 min. 5 min hold time at 100% ACN

===

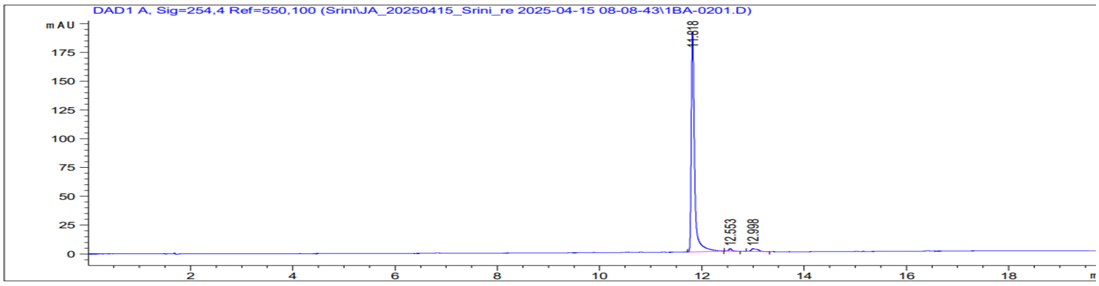

===

Area Percent Report

===

Sorted By : Signal
Multiplier : 1.0000
Dilution : 1.0000
Do not use Multiplier & Dilution Factor with ISTDs

Signal 1: DAD1 A, Sig=254,4 Ref=550,100

Peak #	RetTime [min]	Type	Width [min]	Area [mAU*s]	Height [mAU]	Area %
1	11.818	BB	0.0680	860.73285	191.22368	96.5672
2	12.553	BB	0.0641	9.29763	2.14433	1.0431
3	12.998	BB	0.1167	21.29995	2.40786	2.3897

Totals : 891.33043 195.77587

===

*** End of Report ***

Compound 14c

```
Data File C:\Chem32\3\Data\Srini\JA_20250415_Srini_re 2025-04-15 08-08-43\1BB-0301.D
Sample Name: 13 SRI-2-58-1 re
    =====================================================================
    Acq. Operator   : SYSTEM                              Seq. Line :    3
    Acq. Instrument : 1260 LC_DAD                          Location :   P1-B2
    Injection Date  : 15.04.2025 09:11:02                       Inj :    1
                                                         Inj Volume : 5.000 µl
    Method          : C:\Chem32\3\Data\Srini\JA_20250415_Srini_re 2025-04-15 08-08-43\KinetexC18_
                      gradient20min.M (Sequence Method)
    Last changed    : 15.04.2025 08:08:43 by SYSTEM
    Method Info     : Standard gradient with C18 column,10:90 ACN +H2O to 100% ACN over 15 min. 5
                      min hold time at 100% ACN
    =====================================================================
```

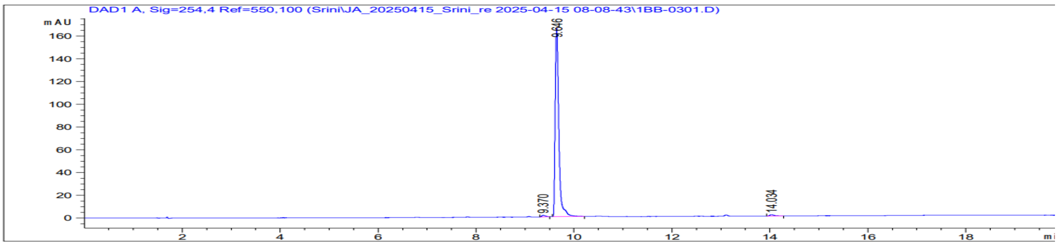


```
    =====================================================================
                             Area Percent Report
    =====================================================================

    Sorted By              :      Signal
    Multiplier             :      1.0000
    Dilution               :      1.0000
    Do not use Multiplier & Dilution Factor with ISTDs


    Signal 1: DAD1 A, Sig=254,4 Ref=550,100

    Peak RetTime Type  Width     Area      Height     Area
      #   [min]        [min]   [mAU*s]     [mAU]        %
    ----|-------|----|-------|----------|----------|--------|
       1   9.370 BB    0.0661    7.21999    1.42407   0.8264
       2   9.646 BB    0.0799  858.78217  165.90300  98.2949
       3  14.034 BB    0.0830    7.67708    1.11896   0.8787

    Totals :                   873.67923  168.44603

    =====================================================================
                              *** End of Report ***
```

Single 8-point titrations curves for enzymatic CSF1R and KIT activity, including the goodness of fit (R^2) for the regression.

CSF1R LANCE Chart	CSF1R LANCE IC50[µM]	CSF1R LANCE rSquare	KIT Lantha Assay Chart	KIT Lantha Assay Kd[µM]	KIT Lantha Assay rSquare
LDC389091:01 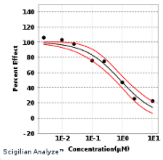	0,86	0,96	LDC389091:01 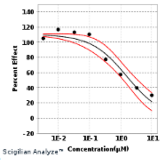	2,00	0,94
LDC389093:01 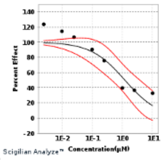	1,21	0,85	LDC389093:01 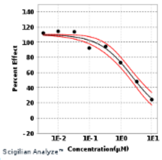	2,90	0,97
LDC389092:01 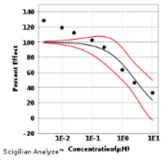	2,69	0,81	LDC389092:01 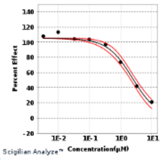	2,63	0,99
LDC389094:01 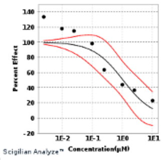	1,05	0,81	LDC389094:01 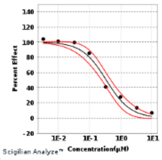	0,35	0,98
LDC392386:01 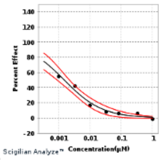	0,00154	0,97	LDC392386:01 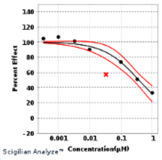	0,375	0,98
LDC392417:01 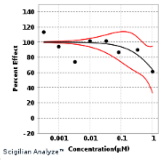	>1.0	0,47	LDC392417:01 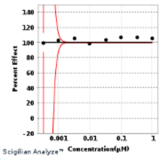	>1.0	-1,33
LDC392422:01 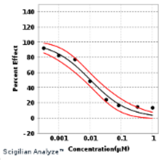	0,0105		LDC392422:01 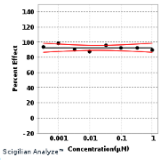	>1.0	0,00
LDC388230:01 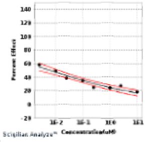	0,0061	0,92	LDC388230:01 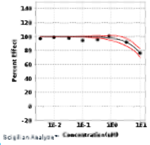	>10.0	0,84
LDC392393:01 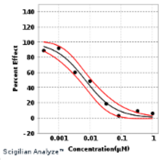	0,0072	0,97	LDC392393:01 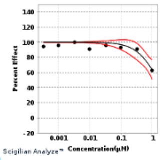	>1.0	0,82
LDC209923:03 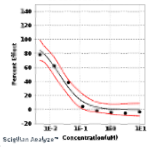	0,021	0,97	LDC209923:06 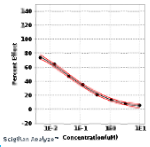	0,036	1,00

Reported ^{1}H and ^{13}C NMR data and ChemDraw predicted ^{1}H and ^{13}C NMR data of the final compounds

Compound: 4a

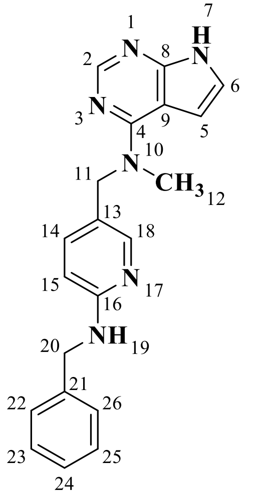

Compound: 4b

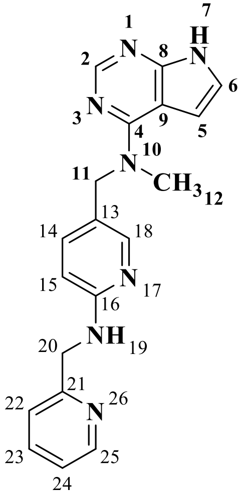

Compound: 4c

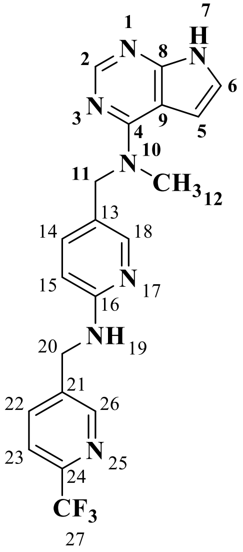

Atom number	Experimental NMR data						ChemDraw predicted values					
	^{1}H NMR			^{13}C NMR			^{1}H NMR			^{13}C NMR		
	**4a**	**4b**	**4c**	**4a**	**4b**	**4c**	**4a**	**4b**	**4c**	**4a**	**4b**	**4c**
1	-	-	-	-	-	-	-	-	-	-	-	-
2	8.13 (s)	8.18 (s)	8.12 (s)	150.7	150.7	150.7	8.27	8.27	8.27	153.8	153.8	153.8
3	-	-	-	-	-	-	-	-	-	-	-	-
4	-	-	-	156.6	156.6	156.6	-	-	-	167.7	167.7	167.7

5	6.46 (d)	6.60-6.56 (m)	6.54-6.51 (m)	101.4	101.4	101.4	6.72	6.72	6.72	99.2	99.2	99.2
6	7.92 (d)	7.95 (d)	7.91 (d)	120.7	120.7	120.8	7.92	7.92	7.92	121.5	121.5	121.5
7	11.64 (s)	11.68 (s)	11.64 (s)	-		-	11.12	11.12	11.12	-	-	-
8	-	-	-	151.6	151.6	151.6	-	-	-	150.0	150.0	150.0
9	-	-	-	101.9	101.9	101.9	-	-	-	102.3	102.3	102.3
10	-	-	-	-		-	-	-	-	-	-	-
11	4.79 (s)	4.85 (s)	4.79 (s)	49.8	49.8	49.7	4.71	4.71	4.71	58.4	58.4	58.4
12	3.24 (s)	3.38 (s)	3.24 (s)	36.6	36.7	36.7	3.03	3.03	3.03	39.1	39.1	39.1
13	-	-	-	121.1	121.3	120.3	-	-	-	122.0	122.0	122.0
14	6.54 (dd)	6.60-6.56 (m)	7.34 (dd)	136.4	136.5	136.7	7.45	7.45	7.45	137.4	137.4	137.4
15	7.21-7.18 (m)	7.29-7.24 (m)	7.82 (d)	108.0	108.2	108.5	6.62	6.62	6.62	106.9	106.9	106.9
16	-	-	-	158.0	157.8	157.5	-	-	-	156.2	156.2	156.2
17	-	-	-	-		-	-	-	-	-	-	-
18	7.11 (s)	7.17-7.13 (m)	6.54-6.51	146.7	146.7	146.6	8.03	8.03	8.03	148.9	148.9	148.9
19	7.00 (t)	7.17-7.13 (m)	7.22 (t)	-	-	-	6.83	6.81	6.83	-	-	-
20	4.44 (d)	4.57 (d)	4.56 (d)	44.1	46.2	41.5	4.35	4.72	4.35	46.4	47.8	42.1
21	-	-	-	140.6	159.9	149.3	-	-	-	139.9	158.7	129.6
22	7.31-7.27 (m)	7.34 (d)	7.95 (dd)	127.1	120.8	121.7	7.31-7.29	7.31	7.76	126.9	121.0	135.3
23	7.31-7.27 (m)	7.75 (td)	7.12-7.10 (m)	128.1	136.5	140.5	7.31-7.29	7.74	7.30	128.5	139.6	121.9
24	7.31-7.27 (m)	8.54 (dd)	-	126.4	121.7	144.7	7.31-7.29	7.24	-	126.7	120.9	141.4
25	7.31-7.27 (m)	7.39-7.33 (m)	-	128.1	136.5	-	7.31-7.29	8.51	-	128.5	148.6	-
26	7.31-7.27 (m)	-	8.70 (s)	127.1	-	136.7	7.31-7.29	-	8.51	126.9	-	149.4
27	-	-	-	-	-	121.8	-	-	-	-	-	119.7

Compound: 8

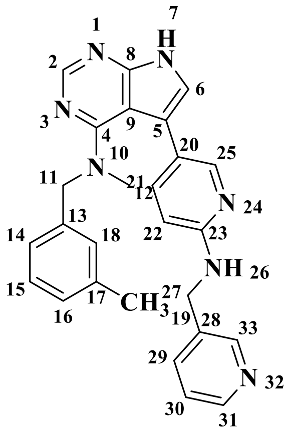

Atom number	Experimental NMR data		Chemdraw predicted values	
	^{1}H NMR	^{13}C NMR	^{1}H NMR	^{13}C NMR
	**8**	**8**	**8**	**8**
1	-	-	-	-
2	8.24 (s)	152.9	8.27	153.8
3	-	-	-	-
4	-	156.9	-	167.7
5	-	102.1	-	108.6
6	6.53 (d)	120.6	6.86	118.2
7	11.94 (s)	-	9.50	-
8	-	149.9	-	151.0
9	-	107.8	-	108.6
10	-	-	-	-
11	4.52 (s)	54.7	4.71	58.7
12	2.57 (s)	41.9	3.03	39.1

13	-	137.38	-	136.3
14	7.50 (dd)	123.3	7.21	124.9
15	7.32-7.29 (m)	128.1	7.51	128.4
16	7.16-7.11 (m)	127.6	7.05	127.3
17	-	137.7	-	138.2
18	6.96 (s)	128.4	7.19	129.8
19	2.25 (s)	21.0	2.31	21.6
20	-	124.9	-	125.9
21	7.02 (d)	136.0	7.78	134.8
22	7.16-7.11 (m)	113.3	6.82	110.1
23	-	159.8	-	156.8
24	-	-	-	-
25	8.11 (d)	146.2	8.45	142.3
26	6.89 (t)	-	6.83	-
27	4.50 (s)	45.6	4.35	42.1
28	-	135.0	-	132.9
29	7.71 (d)	137.30	7.86	135.5
30	7.32-7.29 (m)	120.6	7.37	123.0
31	8.43 (dd)	147.8	8.37	147.3
32	-	-	-	-
33	8.56 (d)	148.8	8.59	148.7

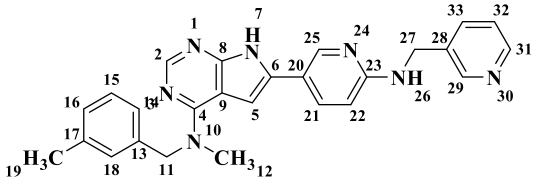

Compound: 12b

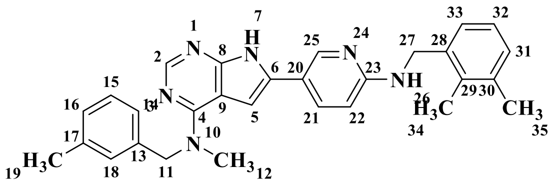

Compound: 12d

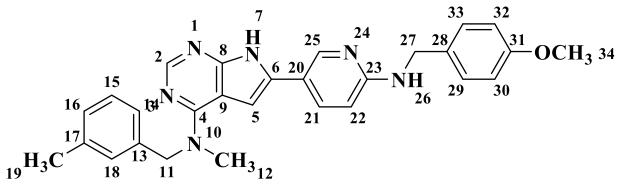

Compound: 12e

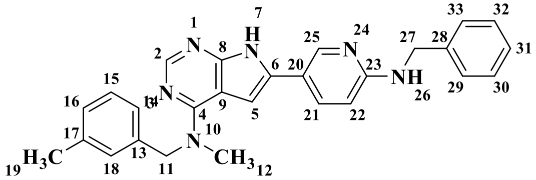

Compound: 14a

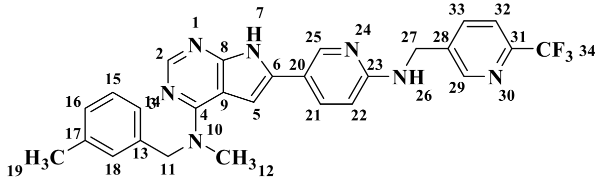

Compound: 14c

Atom number	**Experimental NMR data**										**Chemdraw predicted values**									
	**^{1}H NMR**					**^{13}C NMR**					**^{1}H NMR**					**^{13}C NMR**				
	**12b**	**12d**	**12e**	**14a**	**14c**	**12b**	**12d**	**12e**	**14a**	**14c**	**12b**	**12d**	**12e**	**14a**	**14c**	**12b**	**12d**	**12e**	**14a**	**14c**
1	-	-	-	-	-	-	-	-	-	-	-	-	-	-	-	-	-	-	-	-
2	8.11 (s)	8.15 (s)	8.10 (s)	8.11 (s)	8.11 (s)	152.7	153.0	152.7	152.7	152.7	8.27	8.27	8.27	8.27	8.27	153.8	153.8	153.8	153.8	153.8
3	-	-	-	-	-	-	-	-	-	-	-	-	-	-	-	-	-	-	-	-
4	-	-	-	-	-	156.0	155.0	156.0	156.0	156.0	-	-	-	-	-	167.7	167.7	167.7	167.7	167.7
5	6.58 (s)	7.08 (s)	7.08 (s)	6.81 (s)	6.84 (s)	103.2	103.2	103.2	103.2	103.2	6.48	6.48	6.48	6.48	6.48	108.1	108.1	108.1	108.1	108.1
6	-	-	-	-	-	131.8	127.8	132.2	128.3	133.7	-	-	-	-	-	129.9	129.9	129.9	129.9	129.9
7	11.99 (s)	11.21 (s)	11.98 (s)	11.99 (s)	12.01 (s)	-	-	-	-	-	11.94	11.94	11.94	11.94	11.94	-	-	-	-	-

8	-	-	-	-	-	150.5	151.1	150.4	150.4	150.5	-	-	-	-	-	149.6	149.6	149.6	149.6	149.6
9	-	-	-	-	-	96.3	98.5	96.1	96.1	96.5	-	-	-	-	-	100.4	100.4	100.4	100.4	100.4
10	-	-	-	-	-	-	-	-	-	-	-	-	-	-	-	-	-	-	-	-
11	4.98 (s)	5.45 (s)	4.98 (s)	4.98 (s)	4.98 (s)	52.5	52.5	52.5	52.5	52.5	4.71	4.71	4.71	4.71	4.71	58.7	58.7	58.7	58.7	58.7
12	3.32 (s)	3.34 (s)	3.32 (s)	3.32 (s)	3.32 (s)	37.2	21.0	37.2	37.2	37.2	3.03	3.03	3.03	3.03	3.03	39.1	39.1	39.1	39.1	39.1
13	-	-	-	-	-	137.5	133.7	133.5	133.5	136.7	-	-	-	-	-	136.3	136.3	136.3	136.3	136.3
14	7.07-7.03 (m)	7.06-7.04 (m)	7.21-7.16 (m)	7.08-7.04 (m)	7.07-7.04 (m)	124.1	125.0	124.1	124.1	124.1	7.21	7.21	7.21	7.21	7.21	124.9	124.9	124.9	124.9	124.9
15	7.07-7.03 (m)	7.06-7.04 (m)	7.05 (t)	7.34-7.18 (m)	7.45 (t)	127.54	127.5	127.54	127.5	127.5	7.51	7.51	7.51	7.51	7.51	128.4	128.4	128.4	128.4	128.4
16	7.07-7.03 (m)	7.06-7.04 (m)	7.21-7.16 (m)	7.08-7.04 (m)	7.07-7.04 (m)	127.57	125.0	127.57	127.5	128.4	7.05	7.05	7.05	7.05	7.05	127.3	127.3	127.3	127.3	127.3
17	-	-	-	-	-	138.5	136.1	137.5	138.4	137.5	-	-	-	-	-	138.2	138.2	138.2	138.2	138.2
18	7.35-7.31 (m)	7.33 (d)	7.21-7.16 (m)	7.08-7.04 (m)	7.07-7.04 (m)	128.4	127.8	128.4	127.5	131.7	7.19	7.19	7.19	7.19	7.19	129.8	129.8	129.8	129.8	129.8
19	2.26 (s)	3.32 (s)	2.26 (s)	2.26 (s)	2.26 (s)	21.0	21.0	21.0	21.0	21.0	2.31	2.31	2.31	2.31	2.31	21.6	21.6	21.6	21.6	21.6
20	-	-	-	-	-	123.3	123.3	116.0	116.1	116.7	-	-	-	-	-	122.8	122.8	122.8	122.8	122.8
21	7.84 (dd)	8.09 (dd)	7.80 (dd)	7.82 (dd)	7.87-7.84 (m)	135.0	130.5	132.2	132.0	136.7	7.78	7.78	7.78	7.78	7.78	134.8	134.8	134.8	134.8	134.8
22	6.82 (d)	6.93-6.89 (m)	6.53 (d)	6.56 (d)	6.62 (d)	108.3	114.2	108.0	108.0	108.5	6.82	6.82	6.82	6.82	6.82	110.1	110.1	110.1	110.1	110.1

23	-	-	-	-	-	157.5	156.3	157.7	157.7	157.2	-	-	-	-	-	156.8	156.8	156.8	156.8	156.8
24	-	-	-	-	-	-	-	-	-	-	-	-	-	-	-	-	-	-	-	-
25	8.47 (d)	8.75 (d)	8.46 (d)	8.46 (d)	8.46 (d)	144.2	144.1	144.3	144.3	144.2	8.45	8.45	8.45	8.45	8.45	142.3	142.3	142.3	142.3	142.3
26	7.20 (t)	7.20 (t)	7.05 (t)	7.08-7.04 (m)	7.20 (t)	-	-	-	-	-	6.83	6.83	6.83	6.83	6.83	-	-	-	-	-
27	4.53 (d)	5.00 (d)	4.42 (d)	4.51 (d)	4.64 (d)	41.8	37.3	43.6	44.1	41.5	4.35	4.35	4.35	4.35	4.35	42.1	43.9	46.4	46.4	42.1
28	-	-	-	-	-	133.6	136.2	132.0	137.5	138.4	-	-	-	-	-	132.9	140.5	132.2	139.9	129.6
29	8.56 (d)	-	7.26 (d)	7.34-7.18 (m)	8.74 (s)	148.8	138.3	128.5	127.1	127.5	8.59	-	7.12	7.31	8.51	148.7	133.7	130.5	126.9	149.4
30	-	-	6.87 (d)	7.34-7.18 (m)	-	-	133.3	113.6	128.1	-	-	-	6.89	7.31	-	-	136.6	114.1	128.5	-
31	7.72 (d)	6.99-6.97 (m)	-	7.34-7.18 (m)	-	147.8	128.4	158.0	126.5	144.7	8.37	7.21	-	7.29	-	147.3	128.3	158.6	126.7	141.4
32	7.35-7.31 (m)	6.93-6.89 (m)	6.81 (d)	7.34-7.18 (m)	7.98 (d)	116.4	124.1	113.6	128.1	140.4	7.37	7.41	6.89	7.31	7.30	123.0	125.4	114.1	128.5	121.9
33	8.43 (dd)	7.33 (d)	7.26 (d)	7.34-7.18 (m)	7.87-7.84 (m)	135.9	121.3	128.5	127.1	120.4	7.86	7.25	7.12	7.31	7.71	135.5	122.3	130.5	126.9	135.3
34	-	2.25 (s)	3.72 (s)	-	-		14.2	55.0	121.8	121.8		2.18	3.81		-		16.3	55.8		119.7
35		2.26 (s)					19.9					2.29					19.1			
